# The PIFA-initiated oxidative cyclization of 2-(3-butenyl)quinazolin-4(3*H*)-ones – an efficient approach to 1-(hydroxymethyl)-2,3-dihydropyrrolo[1,2-*a*]quinazolin-5(1*H*)-ones

**DOI:** 10.3762/bjoc.17.189

**Published:** 2021-11-25

**Authors:** Alla I Vaskevych, Nataliia O Savinchuk, Ruslan I Vaskevych, Eduard B Rusanov, Oleksandr O Grygorenko, Mykhailo V Vovk

**Affiliations:** 1 Institute of Organic Chemistry, National Academy of Sciences of Ukraine, Murmanska str. 5, Kyiv 02660, Ukraine; 2 Enamine Ltd, Chervonotkatska str. 78, Kyiv 02094, Ukraine; 3 Taras Shevchenko National University of Kyiv, Kyiv 01601, Ukraine

**Keywords:** [bis(trifluoroacetoxy)iodo]benzene PIFA, nitrogen heterocycles, oxidative cyclization, pyrrolo[1,2-*a*]quinazolines

## Abstract

A regioselective method for the synthesis of 1-(hydroxymethyl)-2,3-dihydropyrrolo[1,2-*a*]quinazolin-5(1*H*)-ones – close structural analogs of naturally occurring vasicinone alkaloids – is described. The procedure is based on PIFA-initiated oxidative 5-*exo-trig* cyclization of 2-(3-butenyl)quinazolin-4(3*Н*)-ones, in turn prepared by thermal cyclocondensation of the corresponding 2-(pent-4-enamido)benzamides. The products obtained have a good natural product likeness (NPL) score and therefore can be useful for the design of natural product-like compound libraries.

## Introduction

**Figure 1:**
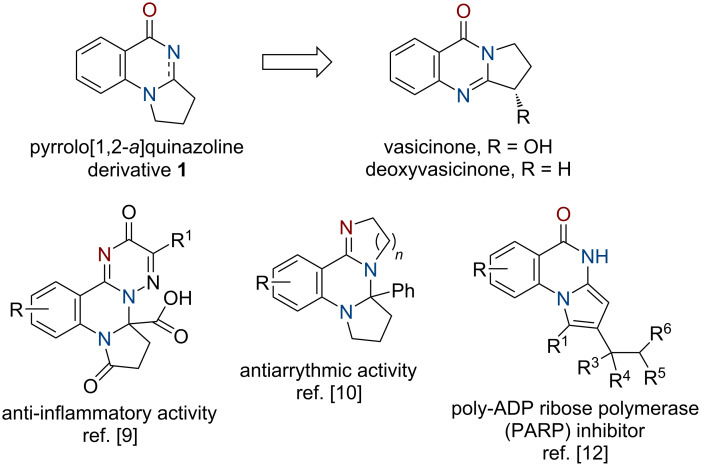
Pyrrolo[1,2-*a*]quinazoline derivatives – analogs of vasicinone alkaloids and their biological activity.

An important design concept in current drug discovery includes structural modifications of naturally occurring compounds to provide novel, sp^3^-enriched scaffolds with increased propensity to generate potent lead structures with favorable physicochemical properties [1–4]. In light of this, synthesis of compounds that are close analogs of natural compounds is an important task for the synthetic organic chemistry, and this approach has already provided successful results in the view of reaching biological activity. Thus, angular pyrrolo[1,2-*a*]quinazolines of type **1** – analogs of naturally occurring vasicinone alkaloids bearing an isomeric linear pyrrolo[2,1-*b*]quinazoline core [5–8] – demonstrated anti-inflammatory [9], antibacterial [10], antiarrythmic [11] activity; some representatives are CNS suppressors and poly-ADP ribose polymerase (PARP) inhibitors [12] (see Figure 1).

**Scheme 1:**
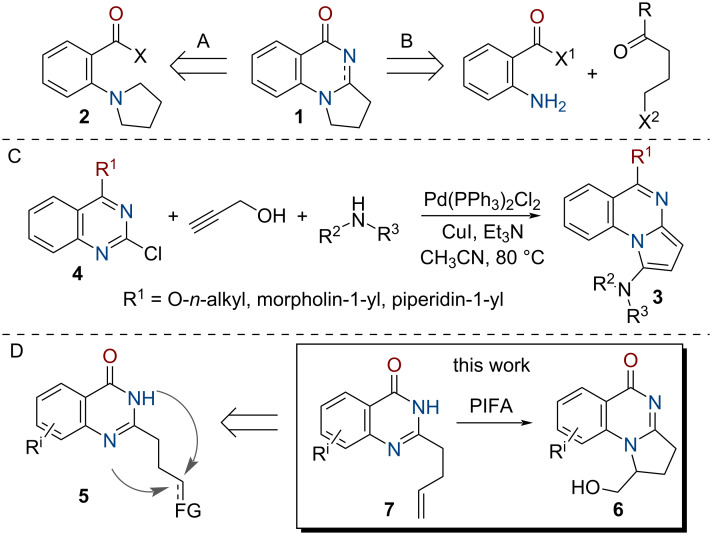
Synthetic approaches to 2,3-dihydropyrrolo[1,2-*a*]quinazolin-5(1*H*)-one derivatives.

Several approaches to obtain 2,3-dihydropyrrolo[1,2-*a*]quinazolin-5(1*H*)-one derivatives of type **1** are known in the literature to date. One of them is based on the construction of the central pyrimidine ring starting from *ortho*-disubstituted aromatic compounds **2** bearing a pyrrolidine moiety (see Scheme 1A). Examples include cyclocondensation of (2-pyrrolidin-1-yl)benzaldehyde and aniline occurring in the presence of TsOH [13], as well as Ir-catalyzed intramolecular dehydrative cross-coupling of 2-(pyrrolidine-1-yl)benzamide [14].

Another approach to compounds of type **1** that relies on a cascade formation of the pyrimidine and pyrrole rings have found much wider application (see Scheme 1B). One of its variations includes the reaction of readily available anthranilic amides or hydrazides with pent-4-yn-1-ols or -carboxylic acids promoted by PdCl_4_ [15], Au(І) [16–18] or Cu(ІІ) [19] salts. In another version, the target compounds were obtained by iodine-catalyzed reaction of the aforementioned anthranilic acid derivatives with 4-chloroketones or 4-oxocarboxylic acids in ionic liquids [20–23] or refluxing acetic acid [24–25]. An alternative approach involves cascade cyclization of anthranilamides with 4-chlorobutanoyl chloride [26–27]. Also, reductive cyclocondensation of 2-nitrobenzamides and halogenoketones or ketocarboxylic acids upon action of SnCl_2_·H_2_O, TiCl_4_/Zn, or Fe/CH_3_COOH can be mentioned [28–30].

1-Aryl-substituted tetrahydropyrrolo[1,2-*a*]quinazolin-5-ones can be also obtained by a Brønsted acid-catalyzed annulation of arylcyclopropane aldehydes and *N′*-anthranilic hydrazides [31], as well as by Sm(OTf)_3_-catalyzed stereoselective [3 + 2] cycloaddition of bis-silyldienediolate and imines, in turn synthesized from anthranylamides and benzaldehydes [32].

A promising approach to the synthesis of 2,3-dihydropyrrolo[1,2-*a*]quinazolin-5(1*H*)-one derivatives substituted at the pyrrolidine ring (especially with functional groups) is annulation of the latter moiety to the quinazoline ring. Thus, a series of 1,5-disubstituted pyrroloquinazolines **3** were obtained by a three-component Sonogashira-type coupling of 2-chloro-4-substituted quinazolines **4**, propargylic alcohol, and secondary amines (see Scheme 1C) [10]. Appropriately 2-functionalized quinazolinones of type **5** might be good starting materials for the intramolecular cyclization providing the target compounds (see Scheme 1D); however, this approach was rarely used [33–36], possibly due to low regioselectivity of the ring formation at the two competing nucleophilic centers.

## Results and Discussion

In this work, we propose a regioselective approach to the synthesis of 2,3-dihydropyrroloquinazolin-5(1*H*)-ones **6** functionalized with a hydroxymethyl group by oxidative cyclization of hereto unknown 2-(buten-3-yl)quinazolin-4(3*H*)-ones **7** upon action of bis(trifluoroacetoxy)iodobenzene (PIFA) (see Scheme 1D). A part from the well-known applications of hypervalent iodine compounds for oxidative rearrangements, fragmentations, halogenations and hydroxylations [37–38], they were also involved in the synthesis of N-heterocycles [39–40] including from properly functionalized arenes and alkenes [41–53]. In the case of such substrates having oxygen-containing functional groups, PIFA attacked the double bond first [41–43]. With unsaturated amides or hydroxamates, oxidation of the nitrogen atom to nitrenium intermediates occurred initially; further stabilization of these species resulted in the formation of hydroxylated lactams [44–48], azaspirocycles [49–53], or tricyclic nitrogen-containing heterocycles [44,53].

Our study commenced with the synthesis of key intermediates **7** bearing a homoallyl substituent at the C-2 position. Most of the methods for the preparation of 2-akyl-substitued quinazolin-4(3*H*)-ones [54] require the use of hardly available starting materials, expensive catalysts, and/or harsh reaction conditions [55], often intolerant to the unsaturated moieties. The known literature exceptions included synthesis of 2-alkynylquinazolines via acylation of anthranilamides with alkynylcarboxylic acids and further cyclocondensation under alkaline conditions [36], as well as formation of 2-alkenyl counterparts by a one-pot Yb(OTf)_3_-catalyzed microwave- or ultrasound-assisted reaction of 2-aminobenzonitrile and alkenoyl chlorides [56].

We have found that substrates **7** can be obtained efficiently by a two-step reaction sequence commencing from acylation of anthranilamides **8** with α-allylacetyl chloride **9** leading to benzamides **10**. These intermediates appeared to be stable towards heating and underwent intramolecular cyclocondensation performed in diphenyl ether at 230 °C giving target products **7** in good to high yields (see Table 1).

**Table 1:** Synthesis of 2-(buten-3-yl)quinazolin-4(3*H*)-ones **7**.

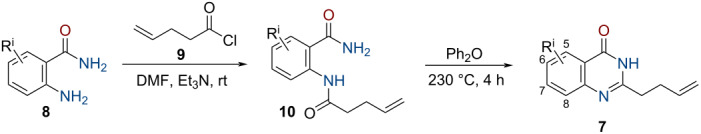						

Entry	Amide	R^i a^	Product **10**	Product **10** yield [%]^b^	Product **7**	Product **7** yield [%]^b^

1	**8a**	H	**10a**	74	**7a**	71
2	**8b**	5-NO_2_	**10b**	91	**7b**	62
3	**8c**	5-F	**10c**	87	**7c**	63
4	**8d**	6-Me	**10d**	75	**7d**	69
5	**8e**	6-OMe	**10e**	71	**7e**	76
6	**8f**	6-Cl	**10f**	89	**7f**	87
7	**8g**	6-NO_2_	**10g**	76	**7g**	91
8	**8h**	6,7-(OMe)_2_	**10h**	67	**7h**	71
9	**8i**	7-Cl	**10i**	83	**7i**	79
10	**8j**	8-Me	**10j**	74	**7j**	72
11	**8k**	8-Br	**10k**	68	**7k**	66
12	**8l**	8-F	**10l**	76	**7l**	63

^a^Atom numbering as in product **7**. ^b^Over two steps.

Since it was not possible to predict a priori which of the nitrogen atoms of **7** would participate in PIFA-promoted heterocyclization, optimization of the reaction conditions was performed. The solvent, reaction temperature and time, as well as the reagent ratio were varied. Since CH_2_Cl_2_, CH_2_Cl_2_–TFA, and 2,2,2-trifluoroethanol (CF_3_CH_2_OH, TFE) are used most often for the reactions with PIFA, these solvents were evaluated in the study (see Table 2). It was found the reaction did not proceed with 1.5 equiv of PIFA in CH_2_Cl_2_ or CH_2_Cl_2_–TFA at 0 °C over 2 h (Table 2, entries 1 and 2), while in TFE, the conversion was 37% (Table 2, entry 3). To achieve full conversion of the starting material **7a** in TFE at 0 °C, 2.5 equiv of the oxidant and 24 h reaction time were necessary (Table 2, entry 10).

**Table 2:** Optimization of the reaction conditions^a^.

Entry	Solvent	Amount of PIFA [equiv]	Temperature [°C]	Time [h]	Conversion [%]^b^

1	CH_2_Cl_2_	1.5	0	2	0
2	CH_2_Cl_2_–TFA	1.5	0	2	0
3	TFE	1.5	0	2	37
4	TFE	1.5	0	6	46
5	TFE	1.5	25	6	100^c^
6	TFE	2.5	0	6	57
7	TFE	3.5	0	6	56
8	TFE	2.5	0	12	85
9	TFE	2.5	0	18	96
10	TFE	2.5	0	24	100
11	TFE	2.5	−15	240	88
12	TFE	3.5	−15	240	100

^a^Reaction conditions: **4a** (0.25 mmol), solvent (10 mL). ^b^The conversion was determined by LC–MS. ^c^The target product content was 70%.

The optimized conditions were applied to all quinazolones **7a–l**, and target 1-(hydroxymethyl)-2,3-dihydropyrrolo[1,2-*a*]quinazolin-5(1*H*)-ones **6a–l** were obtained in 75–83% yield (see Table 3). Their structural analysis showed that the reaction proceeded with high regioselectivity as 5-*exo*-*trig* cyclization. The substituent in the quinazoline ring had virtually no effect at the yield or selectivity of the heterocyclization.

**Table 3:** Synthesis of 1-(hydroxymethyl)-2,3-dihydropyrroloquinazolin-5(1*H*)-ones **6**.

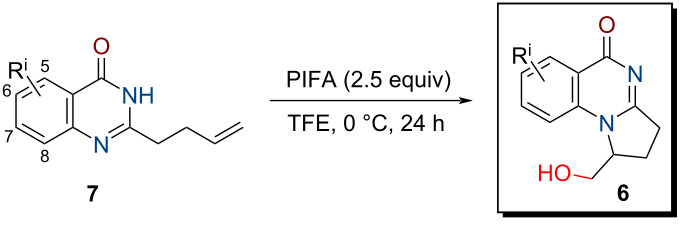					

Entry	Starting compound	R^i a^	Product	Yield [%]	NPL score^b^

1	**7a**	H	**6a**	80	0.42
2	**7b**	5-NO_2_	**6b**	82	−0.10
3	**7c**	5-F	**6c**	83	0.33
4	**7d**	6-Me	**6d**	79	0.54
5	**7e**	6-OMe	**6e**	75	0.57
6	**7f**	6-Cl	**6f**	81	0.24
7	**7g**	6-NO_2_	**6g**	75	0.01
8	**7h**	6,7-(OMe)_2_	**6h**	76	0.69
9	**7i**	7-Cl	**6i**	80	0.23
10	**7j**	8-Me	**6j**	81	0.81
11	**7k**	8-Br	**6k**	76	0.55
12	**7l**	8-F	**6l**	82	0.64

^a^Atom numbering as in starting compound **7**. ^b^Ertl’s natural product likeness (NPL) score calculated using an online NaPLeS tool [4].

**Scheme 2:**
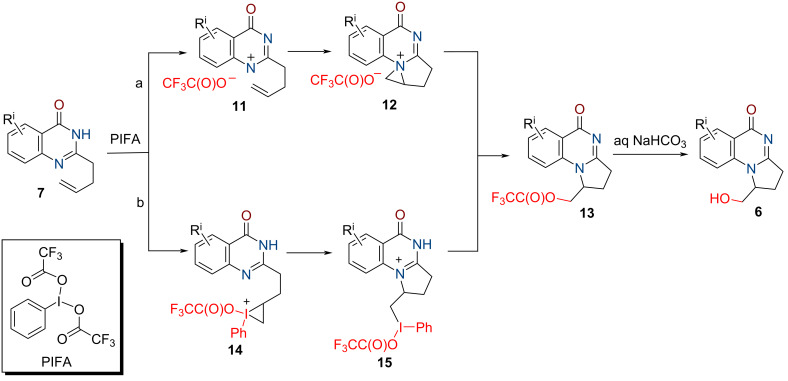
Plausible mechanism for the formation of **6**.

Two pathways seem to be possible for the oxidative heterocyclization of quinazolinones **7** (see Scheme 2). The first of them (pathway a) includes the formation of the nitrene cation **11** [41–42,44–45,53] under action of PIFA as an oxidant. A subsequent electrophilic attack at the double bond provides aziridinium cation **12** that undergoes selective ring opening with the trifluoroacetate anion to give intermediate **13**. The formation of the last-mentioned can be achieved by an initial PIFA attack on the homoallyl C=C bond [41–42,57–58] through the possible intermediates **14** and **15** alternatively (pathway b). Finally, alkaline hydrolysis of **13** upon work-up of the reaction mixture leads to the formation of target product **6**.

**Figure 2:**
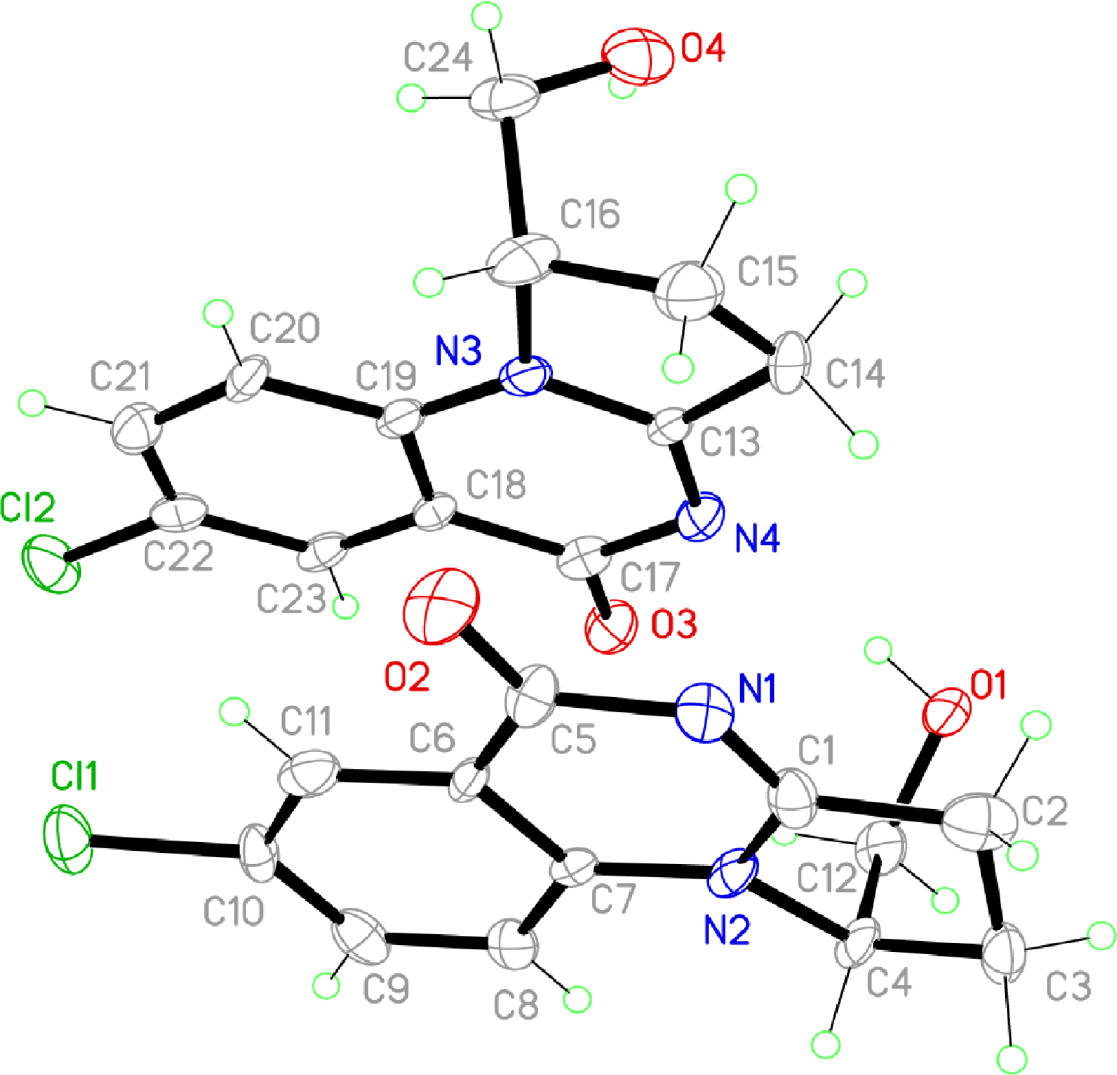
X-ray crystal structure of compound **6f**.

The structure of products **6** was confirmed by NMR spectroscopy; in addition to that, X-ray diffraction studies were performed with single crystals of compound **6f** (see Figure 2).

Since the application of the obtained compounds in early drug discovery is anticipated, it is important to assess their suitability for the synthesis of compound libraries relevant to medicinal chemistry. While many chemoinformatic tools are available for that purpose, we have turned our attention to Ertl’s natural product likeness (NPL) score since the target compounds were designed as natural product analogs [1–4]. In its essence, the NPL score for any molecule estimates its similarity to natural products vs synthetic molecules; it is based on the occurrence frequencies of the corresponding molecular fragments in the two series mentioned above. Zero value of the score is characteristic for the compounds equally similar to natural products and synthetic compounds, while positive values – for those more similar to natural products [1,4].

It was found that most of the compounds **6a–l** had the score values (–0.10 to 0.81; 0.46 on average, see Table 3) – even somewhat higher than natural vasicinone (0.30) and deoxyvasicinone (−0.01). Therefore, the products obtained in this work indeed fit into the natural product-like chemical space.

## Conclusion

A novel PIFA-initiated oxidative cyclization of 2-(but-3-en-1-yl)quinazolin-4(3*H*)-ones into 1-(hydroxymethyl)-2,3-dihydropyrrolo[1,2-*a*]quinazolin-5(1*H*)-ones was found. The reaction proceeds with high regioselectivity when 2.5 equivalents of PIFA are used in 2,2,2-trifluoroethanol solution at 0 °С over 24 h. The synthesized compounds represent a new class of functionalized pyrroloquinazolinones – close analogs of naturally occurring vasicinone alkaloids – that can be used as building blocks to obtain natural product-like compound libraries of potential biologically active compounds.

## Experimental

Commercially available reagents and solvents were used without further purification. The IR spectra of the compounds obtained were recorded on a Bruker Vertex 70 spectrometer in KBr pellets. The NMR spectra were recorded with Varian VXR-300 (400, 500, 600) instruments (300, 400, 600 MHz for ^1^H, 188 MHz for ^19^F and 100, 125, 150 MHz for ^13^C) in CDCl_3_ and DMSO-*d*_6_ solutions, with TMS as an internal standard. Multiplets were assigned as s (singlet), d (doublet), t (triplet), dd (doublet of doublet), q (quartet), m (multiplet) and br s (broad singlet). LC–MS spectra were recorded on an Agilent 1100 Series high-performance liquid chromatograph equipped with a diode matrix with an Agilent LC\MSD SL mass selective detector. Mass spectrometric detection of samples were performed with an Infinity 1260 UHPLC system (Agilent Technologies, Waldbronn, Germany) coupled to a 6224 Accurate Mass TOF LC–MS system (Agilent Technologies, Singapore).

**General procedure for the synthesis of 2-(but-3-en-1-yl)quinazolin-4(3*H*)-ones 7:** A solution of amide **10** (1 mmol) in diphenyl ether (10 mL) was heated at 230 °C for 4 h, then cooled to rt, and diluted with hexanes (20 mL). The precipitate was filtered, washed with *t*-BuOMe/hexanes (1:3), and dried to give product **7**. An analytical sample was obtained by recrystallization from *t*-BuOMe.

**2-(But-3-en-1-yl)quinazolin-4(3*H*)-one (7a)** [59]: Prepared using amide **10a** (218 mg, 1 mmol, 1 equiv). Light brown powder (142 mg, 0.71 mmol, 71%). Mp: 177–178 °C; ^1^H NMR (400 MHz, DMSO-*d*_6_) δ 12.18 (s, 1H, NH), 8.08 (d, *J* = 8.0 Hz, 1H, ArH), 7.77 (t, *J* = 8.0 Hz, 1H, ArH), 7.60 (d, *J* = 8.0 Hz, 1H, ArH), 7.46 (t, *J* = 7.6 Hz, 1H, ArH),5.92–5.82 (m, 1H, CH), 5.07 (d, *J* = 17.2 Hz, 1H, =CH_2_), 4.98 (d, *J* = 10.4 Hz, 1H, =CH_2_), 2.70 (t, *J* = 7.2 Hz, 2H, CH_2_), 2.49–2.47 (m, 2H, CH_2_); ^13^C NMR (125 MHz, DMSO-*d*_6_) δ 161.8, 156.7, 148.9, 137.2, 134.2, 126.8, 125.9, 125.7, 120.9, 115.5, 33.7, 30.6; HRMS–ESI (*m/z*): [M + H]^+^ calcd for C_12_H_12_N_2_O^+^, 201.1023; found, 201.1024.

**General procedure for the PIFA-mediated cyclization of compounds 7a–l:** To a solution of quinazoline **7** (0.65 mmol) in TFE (5 mL), a solution of PIFA (0.70 g, 1.63 mmol) in TFE (20 mL) was added at 0 °С, and the mixture was stirred for 24 h at 0 °С. Saturated aq NaHCO_3_ (20 mL) was added, the precipitate was filtered off, the filtrates were extracted with CH_2_Cl_2_ (3 × 25 mL), dried over Na_2_SO_4_, and the solvent was removed in vacuo. The residue was recrystallized from MeOH to give product **6**.

**1-(Hydroxymethyl)-2,3-dihydropyrrolo[1,2-*a*]quinazolin-5(1*H*)-one (6a):** Prepared using quinazoline **7a** (130 mg, 0.65 mmol, 1 equiv). White solid (112 mg, 0.52 mmol, 80%). Mp 253–255 °C; ^1^H NMR (400 MHz, DMSO-*d*_6_) δ 8.07 (d, *J* = 7.6 Hz, 1H, ArH), 7.77 (t, *J* = 7.6 Hz, 1H, ArH), 7.59 (d, *J* = 7.6 Hz, 1H, ArH), 7.46 (t, *J* = 7.6 Hz, 1H, ArH), 5.02 (t, *J* = 5.6 Hz, 1H, OH), 4.92–4.90 (m, 1H, CH), 3.88–3.82 (m, 1H, CH_2_), 3.67–3.61 (m, 1H, CH_2_), 3.22–3.15 (m, 1H, CH_2_), 2.83–2.81 (m, 1H, CH_2_), 2.44–2.33 (m, 1H, CH_2_), 2.22–2.16 (m, 1H, CH_2_); ^13^C NMR (125 MHz, DMSO-*d*_6_) δ 169.2, 167.4, 138.3, 133.4, 127.6, 125.3, 118.7, 116.1, 62.2, 61.3, 32.2, 22.7; HRMS–ESI (*m/z*): [M + H]^+^ calcd for C_12_H_12_N_2_O_2_^+^, 217.0972; found, 217.0973.

## Supporting Information

File 1 Detailed experimental procedures for all compounds and precursors, X-ray structure determination, ^1^H/^13^C/^19^F NMR spectra for all compounds.
