# Supplementary material for: The PIFA-initiated oxidative cyclization of 2-(3-butenyl)quinazolin-4(3H)-ones – an efficient approach to 1-(hydroxymethyl)-2,3-dihydropyrrolo[1,2-a]quinazolin-5(1H)-ones
Source: Beilstein J Org Chem. 2021 Nov 25;17:2787–94. doi: 10.3762/bjoc.17.189 (PMC8630437; doi:10.3762/bjoc.17.189)
Supplement: File 1 — Detailed experimental procedures for all compounds and precursors, X-ray structure determination, 1H/13C/19F NMR spectra for all compounds. [file Beilstein_J_Org_Chem-17-2787-s001.pdf]

BEILSTEIN JOURNAL OF ORGANIC CHEMISTRY

Supporting Information

for

The PIFA-initiated oxidative cyclization of 2-(3-butenyl)quinazolin-4(3*H*)-ones – an efficient approach to 1-(hydroxymethyl)-2,3-dihydropyrrolo[1,2-*a*]quinazolin-5(1*H*)-ones

Alla I. Vaskevych, Nataliia O. Savinchuk, Ruslan I. Vaskevych, Eduard B. Rusanov, Oleksandr O. Grygorenko and Mykhailo V. Vovk

Beilstein J. Org. Chem. **2021,** *17,* 2787–2794. doi:10.3762/bjoc.17.189

Detailed experimental procedures for all compounds and precursors, X-ray structure determination, $^{1}H/^{13}C/^{19}F$ NMR spectra for all compounds

License and Terms: This is a supporting information file under the terms of the Creative Commons Attribution License (https://creativecommons.org/licenses/by/4.0). Please note that the reuse, redistribution and reproduction in particular requires that the author(s) and source are credited and that individual graphics may be subject to special legal provisions.
The license is subject to the *Beilstein Journal of Organic Chemistry* terms and conditions: (https://www.beilstein-journals.org/bjoc/terms)

Contents

X-ray crystallographic structure of **6f** S3

Detailed experimental procedures for all compounds S6

References S26

^{1}H NMR, ^{13}C NMR and ^{19}F NMR spectra data S27

X-ray crystallographic structure of 6f

Crystal structure of 6f. Two independent molecules were found in the crystal **6f**, which are very close in geometric parameters. The quinazoline bicyclic system in both molecules is planar with rms deviation of fitted atoms 0.0216 and 0.0247 respectively. The distribution of bond lengths and bond angles are typical for such systems [1], thus the C–C and C–N bonds have intermediate values between single and double bonds carbon–carbon and carbon–nitrogen. The five-membered rings are nonplanar, and have the envelope conformation with a dihedral angle of 26.2(3)° between planes N2C1C2C4/C2C3C4 and 26.3(3)° between planes N3C13C14C16/C14C15C16. Molecules in the crystal are packed in columns directed along the *x* axis, which are additionally connected through OH···N hydrogen bonds (Figure S1).

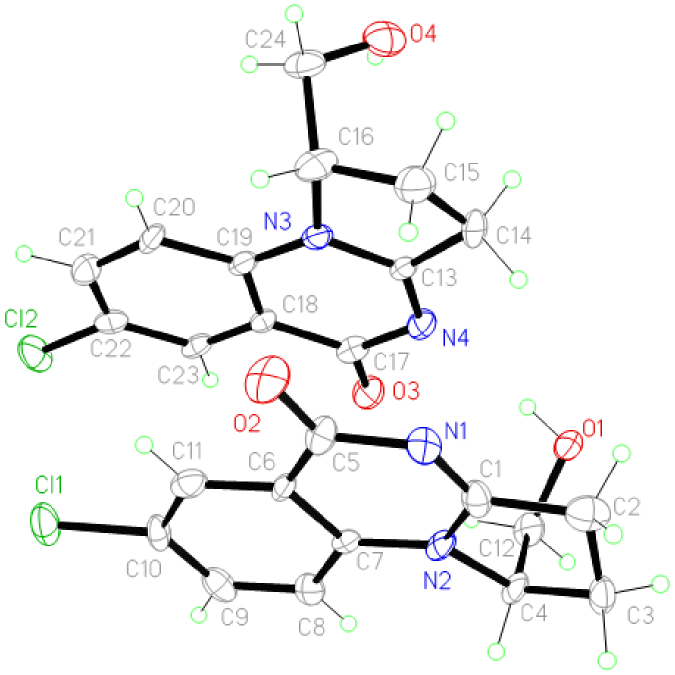

Figure S1: X-ray crystal structure of compound **6f.**

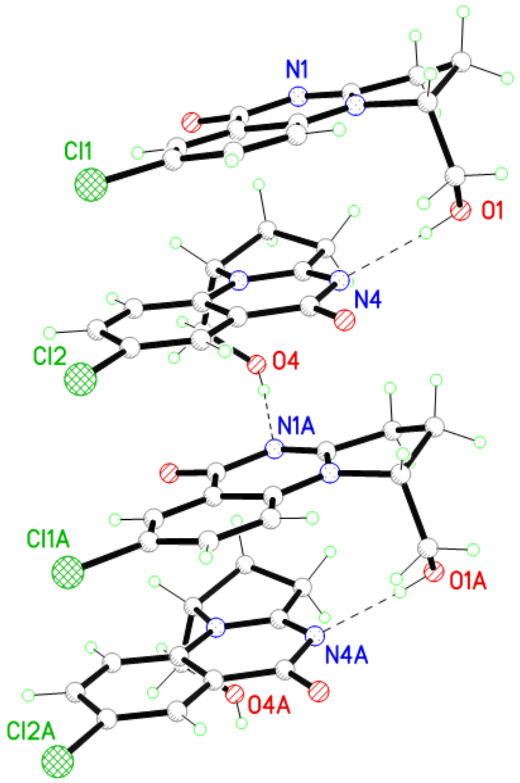

Figure S2: Molecular columns in crystal packing of **6f**.

Crystal data for 6f: $C_{12}H_{11}ClN_2O_2$, M = 250.68, triclinic, space group P-1, *a* = 7.236(2), *b* = 10.871(3), *c* = 14.582(4)Å, α = 74.069(17), β = 87.34(2), γ = 85.77(2)°, V 1099.6(6)Å^3, Z = 4, d_c = 1. 145, μ 0.337 мм$^{-1}$, F(000) 520, crystal size ca. 0.03 x 0.07 x 0.55mm. All crystallographic measurements were performed at 173 K on a Bruker Smart Apex II diffractometer operating in the ω scans mode. The intensity data were collected within the $\theta_{max} \leq 26.4°$ using Mo-K_α radiation (λ = 0.71078 Å). The intensities of 10642 reflections were collected (4114 unique reflections, R_{merg} = 0.1031). The structure was solved by direct methods and refined as a twin by the full-matrix least-squares technique in the anisotropic approximation for non-hydrogen atoms using the Bruker SHELXTL program package [2]. All CH hydrogen atoms were placed at calculated positions and refined as 'riding' model. Convergence for **6f** was obtained at R1 = 0.1131 and wR2 = 0.2856 for 2361 observed reflections with I ≥ 2σ(I); R1 = 0.1723 and wR2 = 0.3266, GOF = 1.031 for 4114 independent

reflections, 310 parameters, the largest and minimal peaks in the final difference map 2.0 and −0.47 e/Å^3.

Crystallographic data for the structures in this paper have been deposited at Cambridge Crystallographic Data Centre as supplementary publication number CCDC2088437. Copies of the data can be obtained, free of charge, on application to CCDC, 12 Union Road, Cambridge CB21EZ, UK, (fax: +44-(0)1223-336033 or e-mail: deposit@ccdc.cam.ac.uk).

Detailed experimental procedures for all compounds

Commercially available reagents and solvents were used without further purification. The IR spectra of the compounds obtained were recorded on a Bruker Vertex 70 spectrometer in KBr pellets. The NMR spectra were recorded with Varian VXR-300 (400, 500, 600) instruments (300, 400, 600 MHz for ^{1}H, 188 MHz for ^{19}F and 100, 125, 150 MHz for ^{13}C) in $CDCl_3$ and DMSO-d_6 solutions, with TMS as an internal standard. Multiplets were assigned as s (singlet), d (doublet), t (triplet), dd (doublet of doublet), q (quartet), m (multiplet) and br s (broad singlet). LC–MS spectra were recorded on an Agilent 1100 Series high performance liquid chromatograph equipped with a diode matrix with an Agilent LC\MSD SL mass selective detector. Mass spectrometric detection of samples were performed with an Infinity 1260 UHPLC system (Agilent Technologies, Waldbronn, Germany) coupled to a 6224 Accurate Mass TOF LC/MS system (Agilent Technologies, Singapore).

General procedure (GP1) for the synthesis of amides 10

To a solution of amine **8** (1.5 mmol) and triethylamine (0.15 g, 1.5 mmol) in DMF (10 mL), allylacetyl chloride **9** (0.18 g, 1.5 mmol) was added dropwise at 0 °C. The resulting mixture was stirred at rt for 3–4 h and then left overnight. The solvent was removed in vacuo, and the residue was triturated with H_2O (20 mL). The crystalline product **10** formed was filtered, dried, and used in the next without additional purification. An analytical sample was obtained by iPrOH or *t*-BuOMe.

2-(Pent-4-enoylamino)benzamide (10a)

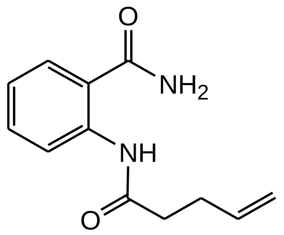

Prepared following the GP1 using amine **8a** (204 mg, 1.5 mmol, 1 equiv). White powder (242 mg, 1.11 mmol, 74%). Mp: 124–125 °C; **^{1}H NMR** (400 MHz, $CDCl_3$) δ 11.18 (s, 1H, NH), 8.66 (d, *J* = 8.4 Hz, 1H, ArH), 7.50–7.54 (m, 2H, ArH), 7.09 (t, *J* = 7.6 Hz, 1H, ArH), 6.13–6.32 (br s, 1H, NH), 5.85–5.94 (m, 1H, CH), 5.61–5.77 (br. s, 1H, NH), 5.12 (d, *J* = 17.6 Hz, 1H, $=CH_2$), 5.04 (d, *J* = 10.4 Hz, 1H, $=CH_2$), 2.48–2.55 (m, 4H, $2CH_2$); **^{13}C NMR** (125 MHz, DMSO-d_6) δ 170.8, 170.3, 139.6, 137.2, 132.2, 128.6, 122.3, 120.2, 119.6, 115.5, 36.7, 28.9; MS: m/z 219 (M+H); Anal. Calcd for $C_{12}H_{14}N_2O_2$: C, 66.04; H, 6.47; N, 12.84; found: C, 66.00; H, 6.43; N, 12.76.

2-Nitro-6-(pent-4-enoylamino)benzamide (10b)

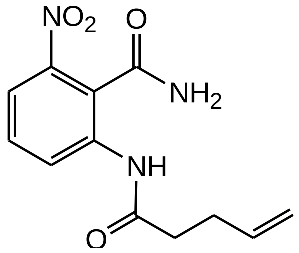

Prepared following the GP1 using amine **8b** (272 mg, 1.5 mmol, 1 equiv). Light yellow powder (359 mg, 1.37 mmol, 91%). Mp: 185–187 °C; **^{1}H NMR** (400 MHz, DMSO-d_6) δ 9.26 (s, 1H, NH), 8.03 (d, *J* = 7.2 Hz, 1H, ArH),), 7.92–7.96 (br. s, 1H, NH), 7.85 (d, *J* = 7.6 Hz, 1H, ArH), 7.66–7.72 (br s, 1H, NH), 7.60 (t, *J* = 8.0 Hz, 1H, ArH), 5.82–5.91 (m, 1H, CH), 5.08 (d, *J* = 17.2 Hz, 1H, $=CH_2$), 4.99 (d, *J* = 9.2 Hz, 1H, $=CH_2$), 2.45–2.49 (m, 2H, CH_2), 2.32–2.35 (m, 2H, CH_2); **^{13}C NMR** (150 MHz, DMSO-d_6) δ 171.7, 165.7, 147.4, 137.8, 136.3, 131.2, 129.8, 127.3, 120.7, 115.8, 35.4, 29.5; MS: m/z 264 (M+H); Anal. Calcd for $C_{12}H_{13}N_3O_4$: C, 54.75; H, 4.98; N, 15.96; found: C, 54.67; H, 4.92; N, 15.88.

2-Fluoro-6-(pent-4-enoylamino)benzamide (10c)

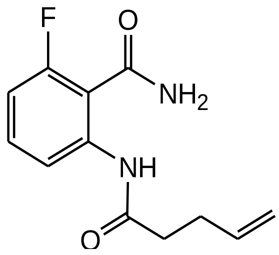

Prepared following the GP1 using amine **8c** (231 mg, 1.5 mmol, 1 equiv). White powder (308 mg, 1.31 mmol, 87%). Mp: 117–118 °C; **^{1}H NMR** (400 MHz, DMSO-*d*$_6$) δ 10.08 (s, 1H, NH), 7.97–8.02 (br. s, 1H, NH), 7.94–7.96 (br s, 1H, NH), 7.88 (d, *J* = 8.4 Hz, 1H, ArH), 7.42 (dd, *J* = 8.4, 7.5 Hz, 1H, ArH), 7.60 (t, *J* = 9.2 Hz, 1H, ArH), 5.79–5.89 (m, 1H, CH), 5.07 (d, *J* = 17.2 Hz, 1H, =CH$_2$), 4.98 (d, *J* = 10.8 Hz, 1H, =CH$_2$), 2.43 (t, *J* = 7.0 Hz, 2H, CH$_2$), 2.30–2.35 (m, 2H, CH$_2$); **^{13}C NMR** (125 MHz, DMSO-*d*$_6$) δ 171.0, 165.7, 159.6 (d, $^1J_{C\text{-}F}$ = 245.0 Hz), 138.8 (d, $^3J_{C\text{-}F}$ = 5.0 Hz), 137.6, 131.8, 131.7, 118.4, 115.8, 111.2 (d, $^2J_{C\text{-}F}$ = 22.5 Hz), 36.3, 29.3; MS: m/z 237 (M+H); Anal. Calcd for $C_{12}H_{13}FN_2O_2$: C, 61.01; H, 5.55; N, 11.86; found: C, 59.94; H, 5.46; N, 11.83.

5-Methyl-2-(pent-4-enoylamino)benzamide (10d)

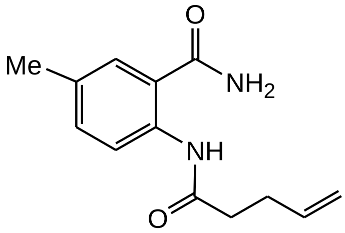

Prepared following the GP1 using amine **8d** (225 mg, 1.5 mmol, 1 equiv). White powder (261 mg, 1.13 mmol, 75%). Mp: 144–145 °C; **^{1}H NMR** (400 MHz, DMSO-*d*$_6$) δ 11.53 (s, 1H, NH), 8.32 (d, *J* = 8.4 Hz, 1H, ArH), 8.17–8.20 (br. s, 1H, NH), 7.65–7.67 (br s, 1H, NH), 7.61 (s, 1H, ArH), 7.29 (d, *J* = 7.6 Hz, 1H, ArH), 5.79–5.89 (m, 1H, CH), 5.07 (d, *J* = 17.2 Hz, 1H, =CH$_2$), 4.98 (d, *J* = 10.4 Hz, 1H, =CH$_2$), 2.40–2.44 (m, 2H, CH$_2$), 2.32–2.39 (m, 2H, CH$_2$), 2.28 (s, 3H, CH$_3$); **^{13}C NMR** (125 MHz, DMSO-*d*$_6$) δ 170.8, 170.0, 137.3, 137.2, 132.5, 131.3, 128.8, 120.2, 119.7, 115.4, 36.7, 28.9, 20.34; MS: m/z 233 (M+H); Anal. Calcd for $C_{13}H_{16}N_2O_2$: C, 67.22; H, 6.94; N, 12.06; found: C, 67.17; H, 6.90; N, 12.01.

5-Methoxy-2-(pent-4-enoylamino)benzamide (10e)

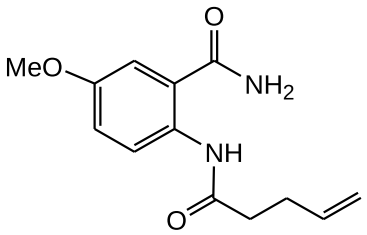

Prepared following the GP1 using amine **8e** (249 mg, 1.5 mmol, 1 equiv). White powder (264 mg, 1.07 mmol, 71%). Mp: 159–161 °C; **^{1}H NMR** (400 MHz, DMSO-d_6) δ 11.28 (s, 1H, NH), 8.31 (d, *J* = 8.8 Hz, 1H, ArH), 8.23–8.25 (br. s, 1H, NH), 7.70–7.73 (br. s, 1H, NH), 7.31 (s, 1H, ArH), 7.08 (d, *J* = 8.8 Hz, 1H, ArH), 5.79–5.89 (m, 1H, CH), 5.07 (d, *J* = 17.2 Hz, 1H, =CH_2), 4.98 (d, *J* = 10.0 Hz, 1H, =CH_2), 3.77 (s, 3H, OCH_3) 2.32–2.41 (m, 4H, 2CH_2); **^{13}C NMR** (125 MHz, DMSO-d_6) δ 170.8, 170.2, 154.6, 137.7, 133.2, 122.5, 121.9, 118.0, 115.8, 113.8, 55.8, 37.0, 29.4; MS: m/z 249 (M+H); Anal. Calcd for $C_{13}H_{16}N_2O_3$: C, 62.89; H, 6.50; N, 11.28; found: C, 62.82; H, 6.44; N, 11.19.

5-Chloro-2-(pent-4-enoylamino)benzamide (10f)

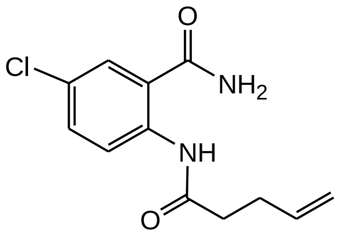

Prepared following the GP1 using amine **8f** (255 mg, 1.5 mmol, 1 equiv). White powder (336 mg, 1.34 mmol, 89%). Mp: 124–126 °C; **^{1}H NMR** (400 MHz, DMSO-d_6) δ 11.57 (s, 1H, NH), 8.46 (d, *J* = 8.8 Hz, 1H, ArH), 8.35–8.38 (br. s, 1H, NH), 7.84–7.88 (m, 2H, ArH + NH), 7.54 (d, *J* = 8.4 Hz, 1H, ArH), 5.79–5.89 (m, 1H, CH), 5.06 (d, *J* = 17.2 Hz, 1H, =CH_2), 4.98 (d, *J* = 10.4 Hz, 1H, =CH_2), 2.44 (t, *J* = 7.2 Hz, 2H, CH_2), 2.33–2.37 (m, 2H, CH_2); **^{13}C NMR** (150 MHz, DMSO-d_6) δ 170.8, 169.9, 138.8, 137.5, 132.2, 128.6, 126.5, 122.4, 121.7, 115.9, 37.1, 29.2; MS: m/z 253 (M+H); Anal. Calcd for $C_{12}H_{13}ClN_2O_2$: C, 57.04; H, 5.19; N, 11.09; found: C, 57.00; H, 5.13; N, 11.05.

5-Nitro-2-(pent-4-enoylamino)benzamide (10g)

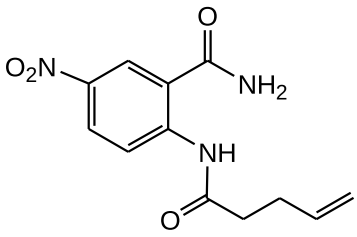

Prepared following the GP1 using amine **8g** (272 mg, 1.5 mmol, 1 equiv). Light yellow powder (300 mg, 1.14 mmol, 76%). Mp: 175–177 °C; **^{1}H NMR** (400 MHz, DMSO-d_6) δ 12.08 (s, 1H, NH), 8.68–8.72 (m, 3H, 2ArH + NH), 8.36 (d, J = 9.2 Hz, 1H, ArH), 8.03–8.05 (br s, 1H, NH), 5.80–5.90 (m, 1H, CH), 5.08 (d, J = 16.8 Hz, 1H, =CH_2), 4.99 (d, J = 10.4 Hz, 1H, =CH_2), 2.50–2.54 (m, 2H, CH_2), 2.35–2.40 (m, 2H, CH_2); **^{13}C NMR** (125 MHz, DMSO-d_6) δ 171.5, 169.5, 145.7, 141.5, 137.4, 127.8, 124.8, 120.3, 119.4, 116.0, 37.2, 29.0; MS: m/z 264 (M+H); Anal. Calcd for $C_{12}H_{13}N_3O_4$: C, 54.75; H, 4.98; N, 15.96; found: C, 54.68; H, 4.95; N, 15.89.

4,5-Dimethoxy-2-(pent-4-enoylamino)benzamide (10h)

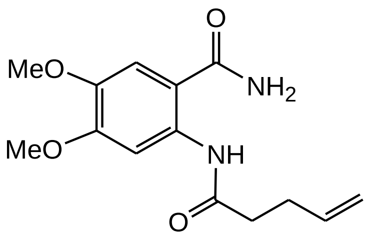

Prepared following the GP1 using amine **8h** (294 mg, 1.5 mmol, 1 equiv). White powder (279 mg, 1.01 mmol, 67%). Mp: 183–185 °C; **^{1}H NMR** (400 MHz, DMSO-d_6) δ 12.11 (s, 1H, NH), 8.29 (s, 1H, ArH), 8.18–8.28 (br. s, 1H, NH), 7.54–7.57 (br s, 1H, NH), 7.35 (s, 1H, ArH), 5.81–5.90 (m, 1H, CH), 5.07 (d, J = 17.2 Hz, 1H, =CH_2), 4.98 (d, J = 10.0 Hz, 1H, =CH_2), 3.77 (2s, 6H, 2OCH_3) 2.34–2.41 (m, 4H, 2CH_2); **^{13}C NMR** (150 MHz, DMSO-d_6) δ 171.1, 170.4, 152.0, 143.7, 137.7, 136.0, 115.8, 112.2, 110.6, 103.9, 56.4, 55.8, 37.3, 29.3; MS: m/z 279 (M+H); Anal. Calcd for $C_{14}H_{18}N_2O_4$: C, 60.42; H, 6.52; N, 10.07; found: C, 60.38; H, 6.44; N, 10.01.

4-Chloro-2-(pent-4-enoylamino)benzamide (10i)

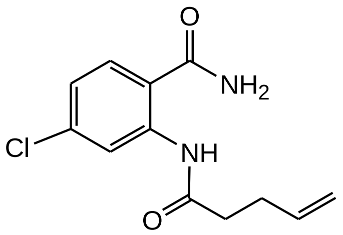

Prepared following the GP1 using amine **8i** (255 mg, 1.5 mmol, 1 equiv). Wheit powder (314 mg, 1.25 mmol, 83%). Mp: 131–133 °C; **^{1}H NMR** (400 MHz, DMSO-d_6) δ 11.88 (s, 1H, NH), 8.57 (s, 1H, ArH), 8.33–8.35 (br s, 1H, NH), 7.81–7.83 (m, 2H, ArH + NH), 7.19 (d, J = 8.4 Hz, 1H, ArH), 5.79–5.89 (m, 1H, CH), 5.07 (d, J = 18.8 Hz, 1H, =CH_2), 4.98 (d, J = 10.4 Hz, 1H, =CH_2), 2.44 (t, J = 7.2 Hz, 2H, CH_2), 2.33–2.38 (m, 2H, CH_2); **^{13}C NMR** (125 MHz, DMSO-d_6) δ 171.1, 170.4, 141.4, 137.4, 137.1, 130.6, 122.4, 119.8, 118.2, 115.9, 37.1, 29.1; MS: m/z 253 (M+H); Anal. Calcd for $C_{12}H_{13}ClN_2O_2$: C, 57.04; H, 5.19; N, 11.09; found: C, 57.03; H, 5.15; N, 11.08.

3-Methyl-2-(pent-4-enoylamino)benzamide (10j)

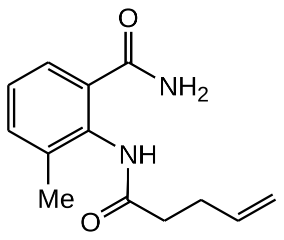

Prepared following the GP1 using amine **8j** (225 mg, 1.5 mmol, 1 equiv). White powder (256 mg, 1.11 mmol, 74%). Mp: 152–153 °C; **^{1}H NMR** (400 MHz, DMSO-d_6) δ 9.46 (s, 1H, NH), 7.50–7.54 (br s, 1H, NH), 7.31–7.35 (m, 3H, ArH + NH), 7.19 (t, J = 7.6 Hz, 1H, ArH), 5.82–5.92 (m, 1H, CH), 5.08 (d, J = 17.2 Hz, 1H, =CH_2), 4.99 (d, J = 10.4 Hz, 1H, =CH_2), 2.31–2.41 (m, 4H, 2CH_2), 2.15 (s, 3H, CH_3); **^{13}C NMR** (125 MHz, DMSO-d_6) δ 170.6, 169.6, 137.7, 135.8, 134.0, 133.8, 131.8, 125.8, 125.7, 115.1, 34.9, 29.2, 18.3; MS: m/z 233 (M+H); Anal. Calcd for $C_{13}H_{16}N_2O_2$: C, 67.22; H, 6.94; N, 12.06; found: C, 67.15; H, 6.86; N, 11.98.

3-Bromo-2-(pent-4-enoylamino)benzamide (10k)

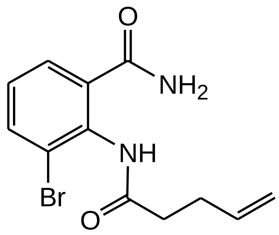

Prepared following the GP1 using amine **8k** (323 mg, 1.5 mmol, 1 equiv). White powder (303 mg, 1.02 mmol, 68%). Mp: 215–217 °C; **^{1}H NMR** (400 MHz, DMSO-d_6) δ 9.62 (s, 1H, NH), 7.74 (d, J = 8.0 Hz, 1H, ArH), 7.51–8.54 (br s, 1H, NH), 7.48 (d, J = 7.6 Hz, 1H, ArH), 7.41–7.44 (br. s, 1H, NH), 7.26 (t, J = 7.6 Hz, 1H, ArH), 5.83–5.93 (m, 1H, CH), 5.08 (d, J = 17.2 Hz, 1H, =CH_2), 4.99 (d, J = 10.0 Hz, 1H, =CH_2), 2.30–2.41 (m, 4H, 2CH_2); **^{13}C NMR** (100 MHz, DMSO-d_6) δ 171.3, 168.6, 138.1, 137.8, 134.4, 134.3, 128.6, 128.0, 123.9, 115.6, 35.1, 29.5; MS: m/z 298 (M+H); Anal. Calcd for $C_{12}H_{13}BrN_2O_2$: C, 48.50; H, 4.41; N, 9.43; found: C, 48.43; H, 4.35; N, 9.39.

3-Fluoro-2-(pent-4-enoylamino)benzamide (10l)

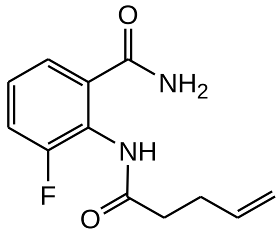

Prepared following the GP1 using amine **8l** (231 mg, 1.5 mmol, 1 equiv). Gray powder (269 mg, 1.14 mmol, 76%). Mp: 177–179 °C; **^{1}H NMR** (400 MHz, DMSO-d_6) δ 9.55 (s, 1H, NH), 7.71–7.74 (br. s, 1H, NH), 7.46–7.50 (br s, 1H, NH), 7.30–7.36 (m, 3H, ArH), 5.80–5.90 (m, 1H, CH), 5.07 (d, J = 17.2 Hz, 1H, =CH_2), 4.98 (d, J = 10.4 Hz, 1H, =CH_2), 2.39 (t, J = 7.0 Hz, 2H, CH_2), 2.29–2.34 (m, 2H, CH_2); **^{13}C NMR** (150 MHz, DMSO-d_6) δ 171.1, 168.5, 157.5 (d,$^1J_{C-F}$ = 246.0 Hz), 137.9, 135.2, 127.4, 124.1 (d,$^4J_{C-F}$ = 3.0 Hz), 124.0 (d,$^3J_{C-F}$ = 13.5 Hz), 117.8 (d,$^2J_{C-F}$ = 21 Hz), 115.6, 35.0, 29.5; MS: m/z 237 (M+H); Anal. Calcd for $C_{12}H_{13}FN_2O_2$: C, 61.01; H, 5.55; N, 11.86; found: C, 59.98; H, 5.48; N, 11.85.

General procedure (GP2) for the synthesis of 2-(but-3-en-1-yl)quinazolin-4(3*H*)-ones 7

A solution of amide **10** (1 mmol) in diphenyl ether (10 mL) was heated at 230 °C for 4 h, then cooled to rt, and diluted with hexanes (20 mL). The precipitate was filtered, washed with *t*-BuOMe–hexanes (1:3), and dried to give product **7**. An analytical sample was obtained by recrystallization from *t*-BuOMe.

2-(But-3-en-1-yl)quinazolin-4(3*H*)-one (7a) [3]

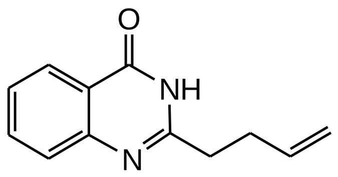

Prepared following the GP2 using amide **10a** (218 mg, 1 mmol, 1 equiv). Light brown powder (142 mg, 0.71 mmol, 71%). Mp: 177–178 °C; **^{1}H NMR** (400 MHz, DMSO-d_6) δ 12.18 (s, 1H, NH), 8.08 (d, *J* = 8.0 Hz, 1H, ArH), 7.77 (t, *J* = 8.0 Hz, 1H, ArH), 7.60 (d, *J* = 8.0 Hz, 1H, ArH), 7.46 (t, *J* = 7.6 Hz, 1H, ArH), 5.82–5.92 (m, 1H, CH), 5.07 (d, *J* = 17.2 Hz, 1H, =CH_2), 4.98 (d, *J* = 10.4 Hz, 1H, =CH_2), 2.70 (t, *J* = 7.2 Hz, 2H, CH_2), 2.47–2.49 (m, 2H, CH_2); **^{13}C NMR** (125 MHz, DMSO-d_6) δ 161.8, 156.7, 148.9, 137.2, 134.2, 126.8, 125.9, 125.7, 120.9, 115.5, 33.7, 30.6; **HRMS** (ESI) *m/z* MH$^+$, found 201.1024. $C_{12}H_{12}N_2O^+$ requires 201.1023.

2-(But-3-en-1-yl)-5-nitroquinazolin-4(3*H*)-one (7b)

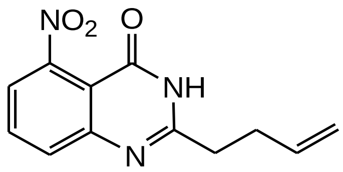

Prepared following the GP2 using amide **10b** (263 mg, 1 mmol, 1 equiv). Yellow powder (152 mg, 0.62 mmol, 62%). Mp: 178–180 °C; **^{1}H NMR** (400 MHz, DMSO-d_6) δ 12.65 (s, 1H, NH), 7.91 (t, *J* = 8.0 Hz, 1H, ArH), 7.82 (d, *J* = 8.0 Hz, 1H, ArH), 7.74 (d, *J* = 7.6 Hz, 1H, ArH),

5.82–5.92 (m, 1H, CH), 5.07 (d, *J* = 17.6 Hz, 1H, =CH_2), 4.99 (d, *J* = 10.4 Hz, 1H, =CH_2), 2.73 (t, *J* = 7.6 Hz, 2H, CH_2), 2.47–2.48 (m, 2H, CH_2); **^{13}C NMR** (150 MHz, DMSO-d_6) δ 159.4, 158.9, 150.2, 148.7, 137.4, 135.0, 130.0, 120.1, 116.2, 111.7, 34.0, 30.9; **HRMS** (ESI) *m/z* MH^+, found 246.0874. $C_{12}H_{11}N_3O_3^+$ requires 246.0873.

2-(But-3-en-1-yl)-5-fluoroquinazolin-4(3*H*)-one (7c)

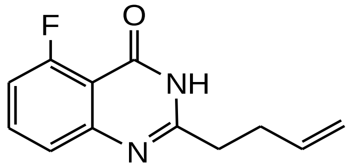

Prepared following the GP2 using amide **10c** (236 mg, 1 mmol, 1 equiv). Light gray powder (137 mg, 0.63 mmol, 63%). Mp: 190–192 °C; **^{1}H NMR** (400 MHz, DMSO-d_6) δ 12.23 (s, 1H, NH), 7.73 (tt, *J* = 6.0, 2.4 Hz, 1H, ArH), 7.40 (d, *J* = 8.4 Hz, 1H, ArH), 7.18 (dd, *J* = 8.0, 2.4 Hz, 1H, ArH), 5.81–5.91 (m, 1H, CH), 5.06 (d, *J* = 17.6 Hz, 1H, =CH_2), 4.98 (d, *J* = 10.0 Hz, 1H, =CH_2), 2.67 (t, *J* = 7.6 Hz, 2H, CH_2), 2.44–2.50 (m, 2H, CH_2); **^{19}F NMR** (188 MHz, DMSO-d_6) δ -114.8 (m, 1F); **^{13}C NMR** (125 MHz, DMSO-d_6) δ 160.9 (d, $^1J_{C-F}$ = 261.3 Hz), 159.5, 158.3, 151.5 137.5, 135.2 (d, $^3J_{C-F}$ = 10.0 Hz), 123.2, 116.0, 112.7 (d, $^2J_{C-F}$ = 20.0 Hz), 110.7 (d, $^4J_{C-F}$ = 6.3 Hz), 33.9, 30.9; **IR/cm^{-1}**: 2916, 1681, 1621, 1474, 1040, 891, 821; **HRMS** (ESI) *m/z* MH^+, found 219.0925. $C_{12}H_{11}FN_2O^+$ requires 219.0928.

2-(But-3-en-1-yl)-6-methylquinazolin-4(3*H*)-one (7d)

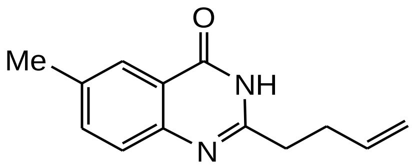

Prepared following the GP2 using amide **10d** (232 mg, 1 mmol, 1 equiv). Light yellow powder (148 mg, 0.69 mmol, 69%). Mp: 213–215 °C; **^{1}H NMR** (400 MHz, DMSO-d_6) δ 12.09 (s, 1H, NH), 7.86 (s, 1H, ArH), 7.59 (d, *J* = 8.4 Hz, 1H, ArH), 7.49 (d, *J* = 8.4 Hz, 1H, ArH), 5.82–5.92 (m, 1H, CH), 5.06 (d, *J* = 17.2 Hz, 1H, =CH_2), 4.97 (d, *J* = 10.4 Hz, 1H, =CH_2), 2.68 (t, *J* = 7.6 Hz, 2H, CH_2), 2.45–2.49 (m, 2H, CH_2), 2.42 (s, 3H, CH_3); **^{13}C NMR** (125 MHz, DMSO-d_6) δ

161.7, 155.8, 146.9, 137.2, 135.5, 135.5, 126.7, 125.1, 120.6, 115.6, 33.7, 30.6, 20.8; **IR/cm^{-1}**: 2895, 1674, 1617, 1488, 912,840; **HRMS** (ESI) *m/z* MH$^+$, found 215.1177. $C_{13}H_{14}N_2O^+$ requires 215.1179.

2-(But-3-en-1-yl)-6-methoxyquinazolin-4(3*H*)-one (7e)

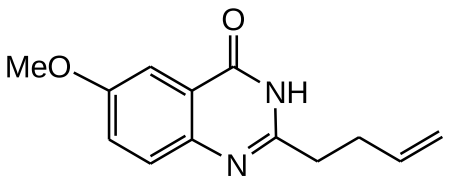

Prepared following the GP2 using amide **10e** (248 mg, 1 mmol, 1 equiv). Light yellow powder (175 mg, 0.76 mmol, 76%). Mp: 195–197 °C; **^{1}H NMR** (400 MHz, DMSO-d_6) δ 12.14 (s, 1H, NH), 7.54 (d, *J* = 8.8 Hz, 1H, ArH), 7.46 (d, *J* = 2.8 Hz, 1H, ArH), 7.37 (dd, *J* = 8.8, 2.8 Hz, 1H, ArH), 5.81–5.91 (m, 1H, CH), 5.06 (d, *J* = 17.6 Hz, 1H, =CH_2), 4.97 (d, *J* = 10.4 Hz, 1H, =CH_2), 3.85 (s, 3H, OCH_3), 2.67 (t, *J* = 7.6 Hz, 2H, CH_2), 2.44–2.49 (m, 2H, CH_2); **^{13}C NMR** (150 MHz, DMSO-d_6) δ 162.0, 157.6, 154.7, 143.8, 137.6, 128.9, 124.1, 121.9, 116.0, 106.1, 56.0, 34.0, 31.1; **IR/cm^{-1}**: 2895, 1670, 1622, 1491, 1365, 1258, 1226, 1035, 910, 845; **HRMS** (ESI) *m/z* MH$^+$, found 231.1131. $C_{13}H_{14}N_2O_2^+$ requires 231.1128.

2-(But-3-en-1-yl)-6-chloroquinazolin-4(3*H*)-one (7f)

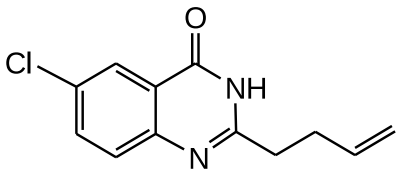

Prepared following the GP2 using amide **10f** (253 mg, 1 mmol, 1 equiv). Light yellow powder (203 mg, 0.87 mmol, 87%). Mp: 206–207 °C; **^{1}H NMR** (400 MHz, DMSO-d_6) δ 12.39 (s, 1H, NH), 7.99 (d, *J* = 2.0 Hz, 1H, ArH), 7.78 (dd, *J* = 8.8, 2.0 Hz, 1H, ArH), 7.61 (d, *J* = 8.4 Hz, 1H, ArH), 5.81–5.91 (m, 1H, CH), 5.06 (d, *J* = 17.2 Hz, 1H, =CH_2), 4.97 (d, *J* = 10.0 Hz, 1H, =CH_2), 2.70 (t, *J* = 7.6 Hz, 2H, CH_2), 2.45–2.49 (m, 2H, CH_2); **^{13}C NMR** (125 MHz, DMSO-d_6) δ 160.7, 157.3, 147.5, 137.1, 134.2, 130.2, 129.0, 124.7, 122.1, 115.6, 33.7, 30.5; **HRMS** (ESI) *m/z* MH$^+$, found 235.0631. $C_{12}H_{11}ClN_2O^+$ requires 235.0633.

2-(But-3-en-1-yl)-6-nitroquinazolin-4(3*H*)-one (7g)

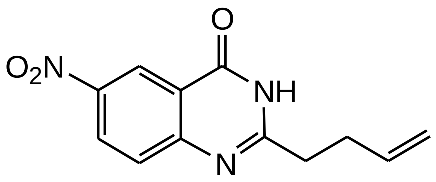

Prepared following the GP2 using amide **10g** (263 mg, 1 mmol, 1 equiv). Light brown powder (169 mg, 0.91 mmol, 91%). Mp: 220–222 °C; **^{1}H NMR** (400 MHz, DMSO-*d*$_6$) δ 12.39 (s, 1H, NH), 8.72 (d, *J* = 2.4 Hz, 1H, ArH), 8.47 (dd, *J* = 8.8, 2.4 Hz, 1H, ArH), 7.74 (d, *J* = 8.8 Hz, 1H, ArH), 5.82–5.92 (m, 1H, CH), 5.07 (d, *J* = 17.2 Hz, 1H, =CH$_2$), 4.99 (d, *J* = 10.4 Hz, 1H, =CH$_2$), 2.74 (t, *J* = 7.6 Hz, 2H, CH$_2$), 2.47–2.53 (m, 2H, CH$_2$); **^{13}C NMR** (125 MHz, DMSO-*d*$_6$) δ 160.8, 160.7, 152.9, 144.2, 136.9, 128.4, 128.0, 121.7, 120.6, 115.7, 33.9, 30.3; **HRMS** (ESI) *m/z* MH$^+$, found 246.0870. $C_{12}H_{11}N_3O_3^+$ requires 246.0873.

2-(But-3-en-1-yl)-6,7-dimethoxyquinazolin-4(3*H*)-one (7h)

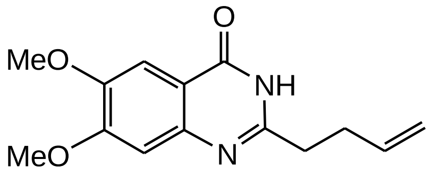

Prepared following the GP2 using amide **10h** (278 mg, 1 mmol, 1 equiv). Light yellow powder (185 mg, 0.71 mmol, 71%). Mp: 258–260 °C; **^{1}H NMR** (400 MHz, DMSO-*d*$_6$) δ 12.04 (s, 1H, NH), 7.40 (s, 1H, ArH), 7.07 (s, 1H, ArH), 5.81–5.91 (m, 1H, CH), 5.06 (d, *J* = 17.2 Hz, 1H, =CH$_2$), 4.98 (d, *J* = 10.0 Hz, 1H, =CH$_2$), 3.87 (s, 3H, OCH$_3$), 3.85 (s, 3H, OCH$_3$), 2.67 (t, *J* = 7.2 Hz, 2H, CH$_2$), 2.45–2.49 (m, 2H, CH$_2$); **^{13}C NMR** (125 MHz, DMSO-*d*$_6$) δ 161.6, 155.5, 154.9, 148.5, 145.4, 137.7, 116.0, 114.1, 108.1, 105.3, 56.3, 56.1, 34.0, 31.1; **HRMS** (ESI) *m/z* MH$^+$, found 261.1229. $C_{14}H_{16}N_2O_3^+$ requires 261.1234.

2-(But-3-en-1-yl)-7-chloroquinazolin-4(3*H*)-one (7i)

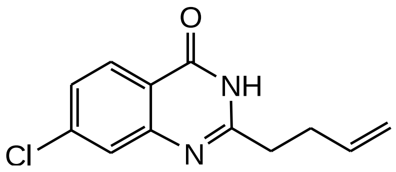

Prepared following the GP2 using amide **10i** (253 mg, 1 mmol, 1 equiv). Light yellow powder (185 mg, 0.79 mmol, 79%). Mp: 184–185 °C; **^{1}H NMR** (400 MHz, DMSO-d_6) δ 12.25 (s, 1H, NH), 8.06 (d, J = 8.8 Hz, 1H, ArH), 7.61 (s, H, ArH), 7.46 (d, J = 8.4 Hz, 1H, ArH), 5.82–5.92 (m, 1H, CH), 5.06 (d, J = 17.2 Hz, 1H, =CH_2), 4.98 (d, J = 10.4 Hz, 1H, =CH_2), 2.70 (t, J = 7.6 Hz, 2H, CH_2), 2.46–2.49 (m, 2H, CH_2); **^{13}C NMR** (150 MHz, DMSO-d_6) δ 161.5, 158.8, 150.3, 139.2, 137.4, 128.1, 126.5, 126.3, 120.0, 116.0, 34.1, 30.9; **IR/cm^{-1}**: 2910, 1682, 1620, 1603, 1428, 1072, 1001, 922, 878; **HRMS** (ESI) *m/z* MH^+, found 235.0635. $C_{12}H_{11}ClN_2O^+$ requires 235.0633.

2-(But-3-en-1-yl)-8-methylquinazolin-4(3*H*)-one (4j)

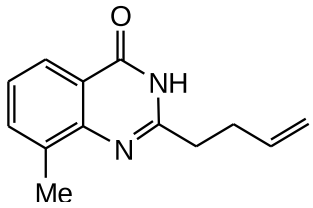

Prepared following the GP2 using amide **10j** (232 mg, 1 mmol, 1 equiv). Light yellow powder (154 mg, 0.72 mmol, 72%). Mp: 169–171 °C; **^{1}H NMR** (400 MHz, $CDCl_3$) δ 11.21–11.31 (br. s, 1H, NH), 8.14 (d, J = 7.6 Hz, 1H, ArH), 7.64 (d, J = 7.2 Hz, H, ArH), 7.38 (t, J = 7.6 Hz, 1H, ArH), 5.93–6.03 (m, 1H, CH), 5.18 (d, J = 16.8 Hz, 1H, =CH_2), 5.08 (d, J = 10.4 Hz, 1H, =CH_2), 2.96 (t, J = 7.6 Hz, 2H, CH_2), 2.69–2.72 (m, 5H, CH_3 + CH_2); **^{13}C NMR** (125 MHz, DMSO-d_6) δ 164.2, 154.4, 147.2, 136.3, 135.3, 134.9, 125.5, 123.4, 119.9, 115.5, 34.3, 30.5, 17.2; **HRMS** (ESI) *m/z* MH^+, found 215.1181. $C_{13}H_{14}N_2O^+$ requires 215.1179.

8-Bromo-2-(but-3-en-1-yl)quinazolin-4(3*H*)-one (7k)

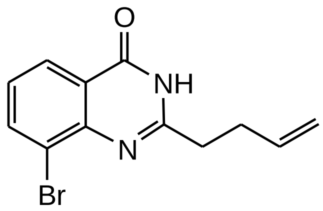

Prepared following the GP2 using amide **10k** (297 mg, 1 mmol, 1 equiv). Light yellow powder (183 mg, 0.66 mmol, 66%). Mp: 205–206 °C; **^{1}H NMR** (400 MHz, DMSO-d_6) δ 12.46 (s, 1H, NH), 8.06–8.10 (m, 2H, 2ArH), 7.36 (t, *J* = 7.6 Hz, 1H, ArH), 5.86–5.96 (m, 1H, CH), 5.10 (d, *J* = 16.8 Hz, 1H, =CH_2), 4.99 (d, *J* = 10.0 Hz, 1H, =CH_2), 2.74 (t, *J* = 7.2 Hz, 2H, CH_2), 2.51–2.55 (m, 2H, CH_2); **^{13}C NMR** (150 MHz, DMSO-d_6) δ 161.7, 158.2, 146.6, 138.1, 137.6, 127.2, 126.0, 122.9, 122.0, 116.1, 34.2, 30.8; **HRMS** (ESI) *m/z* MH$^+$, found 279.0129. $C_{12}H_{11}BrN_2O^+$ requires 279.0128.

2-(But-3-en-1-yl)-8-fluoroquinazolin-4(3*H*)-one (7l)

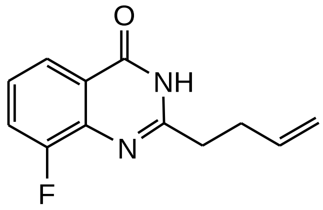

Prepared following the GP2 using amide **10l** (236 mg, 1 mmol, 1 equiv). White powder (137 mg, 0.63 mmol, 63%). Mp: 175–177 °C; **^{1}H NMR** (400 MHz, DMSO-d_6) δ 12.30–12.50 (br. s, 1H, NH), 7.73 (d, *J* = 8.0 Hz, 1H, ArH), 7.61–7.66 (m, 1H, ArH), 7.46–7.41 (m, 1H, ArH), 5.83–5.93 (m, 1H, CH), 5.08 (d, *J* = 17.2 Hz, 1H, =CH_2), 4.99 (d, *J* = 10.0 Hz, 1H, =CH_2), 2.72 (t, *J* = 7.6 Hz, 2H, CH_2), 2.46–2.52 (m, 2H, CH_2); **^{19}F NMR** (188 MHz, DMSO-d_6) δ -127.7 (m, 1F); **^{13}C NMR** (150 MHz, DMSO-d_6) δ 161.5, 158.2, 156.6 (d, $^{1}J_{C\text{-}F}$ = 252.0 Hz), 138.5 (d, $^{3}J_{C\text{-}F}$ = 10.5 Hz), 137.5, 126.4 (d, $^{4}J^{C\text{-}F}$ = 7.5 Hz), 123.4, 121.8, 120.0 (d, $^{2}J_{C\text{-}F}$ = 19.5 Hz), 116.0, 34.4, 31.1; **HRMS** (ESI) *m/z* MH$^+$, found 219.0925. $C_{12}H_{11}FN_2O^+$ requires 219.0928.

General procedure (GP3) for the PIFA-mediated cyclization of compounds 7a–l:

To a solution of quinazoline **7** (0.65 mmol) in TFE (5 mL), a solution of PIFA (0.70 g, 1.63 mmol) in TFE (20 mL) was added at 0 °C, and the mixture was stirred for 24 h at 0 °C. Saturated aq $NaHCO_3$ (20 mL) was added, the precipitate was filtered off, the filtrates were extracted with CH_2Cl_2 (3 × 25 mL), dried over Na_2SO_4, and the solvent was removed in vacuo. The residue was recrystallized from MeOH to give product **6**.

1-(Hydroxymethyl)-2,3-dihydropyrrolo[1,2-*a*]quinazolin-5(1*H*)-one (6a)

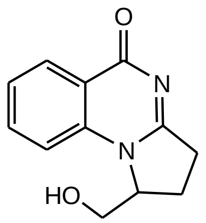

Prepared following the GP3 using quinazoline **7a** (130 mg, 0.65 mmol, 1 equiv). White solid (112 mg, 0.52 mmol, 80%). Mp 253–255 °C; ^{1}**H NMR** (400 MHz, DMSO-*d*$_6$) δ 8.07 (d, *J* = 7.6 Hz, 1H, 1ArH), 7.77 (t, *J* = 7.6 Hz, 1H, ArH), 7.59 (d, *J* = 7.6 Hz, 1H, 1ArH), 7.46 (t, *J* = 7.6 Hz, 1H, ArH), 5.02 (t, *J* = 5.6 Hz, 1H, OH), 4.92-4.90 (m, 1H, CH), 3.88-3.82 (m, 1H, CH_2), 3.67–3.61 (m, 1H, CH_2), 3.22-3.15 (m, 1H, CH_2), 2.83–2.81 (m, 1H, CH_2), 2.44–2.33 (m, 1H, CH_2), 2.22–2.16 (m, 1H, CH_2); ^{13}**C NMR** (125 MHz, DMSO-*d*$_6$) δ 169.2, 167.4, 138.3, 133.4, 127.6, 125.3, 118.7, 116.1, 62.2, 61.3, 32.2, 22.7; **HRMS** (ESI) *m/z* MH^+, found 217.0973. $C_{12}H_{12}N_2O_2^+$ requires 217.0972.

1-(Hydroxymethyl)-6-nitro-2,3-dihydropyrrolo[1,2-*a*]quinazolin-5(1*H*)-one (6b)

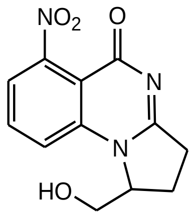

Prepared following the GP3 using quinazoline **7b** (159 mg, 0.65 mmol, 1 equiv). White solid (138 mg, 0.53 mmol, 82%). Mp: 270–272 (decom.) °C; ^{1}**H NMR** (400 MHz, DMSO-*d*$_6$) δ 7.93

(t, J = 8 Hz, 1H, 1ArH), 7.86 (d, J = 8 Hz, 1H, ArH), 7.71 (d, J = 8 Hz, 1H, 1ArH), 5.06 (t, J = 5.8 Hz, 1H, OH), 5.00–4.98 (m, 1H, CH), 3.88–3.83 (m, 1H, CH_2), 3.64–3.59 (m, 1H, CH_2), 3.26–3.16 (m, 1H, CH_2), 2.92–2.85 (m, 1H, CH_2), 2.46–2.33 (m, 1H, CH_2), 2.21-2.15 (m, 1H, CH_2); **^{13}C NMR** (150 MHz, DMSO-d_6) δ 168.6, 166.0, 149.6, 139.8, 134.3, 119.7, 119.4, 109.8, 63.4, 61.9, 32.7, 23.1; **IR/cm^{-1}**: 3217, 1656, 1603, 1546, 1535, 1503, 1418, 1084, 1065, 827; **HRMS** (ESI) *m/z* MH$^+$, found 262.0821. $C_{12}H_{11}N_3O_4^+$ requires 262.0823.

6-Fluoro-1-(hydroxymethyl)-2,3-dihydropyrrolo[1,2-*a*]quinazolin-5(1*H*)-one (6c).

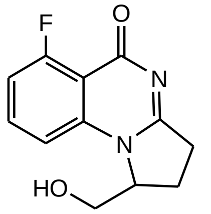

Prepared following the GP3 using quinazoline **7c** (142 mg, 0.65 mmol, 1 equiv). White solid (126 mg, 0.54 mmol, 83%). Mp: 263–265°C (decom.); **^{1}H NMR** (400 MHz, DMSO-d_6) δ 7.77-7.72 (m, 1H, 1ArH), 7.38 (d, J = 8.4 Hz, 1H, 1ArH), 7.20-7.15 (m, 1H, ArH), 5.04 (s, 1H, OH), 4.88–4.85 (m, 1H, CH), 3.85–3.81 (m, 1H, CH_2), 3.63–3.59 (m, 1H, CH_2), 3.22–3.12 (m, 1H, CH_2), 2.86–2.79 (m, 1H, CH_2), 2.43–2.32 (m, 1H, CH_2), 2.20-2.14 (m, 1H, CH_2); **^{19}F NMR** (188 MHz, DMSO-d_6) δ -114.5 (m, 1F); **IR/cm^{-1}**:3206, 2937, 1653, 1619, 1606, 1549, 1505, 1433, 1158, 1085, 816; **HRMS** (ESI) *m/z* MH$^+$, found 235.0876. $C_{12}H_{11}FN_2O_2^+$ requires 235.0878.

1-(Hydroxymethyl)-7-methyl-2,3-dihydropyrrolo[1,2-*a*]quinazolin-5(1*H*)-one (6d).

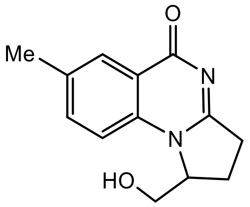

Prepared following the GP3 using quinazoline **7d** (139 mg, 0.65 mmol, 1 equiv). White solid (117 mg, 0.51 mmol, 79%). Mp: 271–273 °C; **^{1}H NMR** (400 MHz, DMSO-d_6) δ 7.87 (s, 1H,

1ArH), 7.60 (d, J = 8.4 Hz, 1H, ArH), 7.51 (d, J = 8.4 Hz, 1H, 1ArH), 5.04 (t, J = 5.6 Hz, 1H, OH), 4.89–4.87 (m, 1H, CH), 3.85–3.80 (m, 1H, CH_2), 3.64–3.59 (m, 1H, CH_2), 3.22–3.12 (m, 1H, CH_2), 2.86–2.79 (m, 1H, CH_2), 2.42–2.30 (m, 4H, CH_3+CH_2), 2.20–2.15 (m, 1H, CH_2); **^{13}C NMR** (125 MHz, DMSO-d_6) δ 169.7, 167.2, 136.7, 135.2, 134.9, 127.6, 119.1, 116.5, 62.6, 61.7, 32.6, 23.1, 21.1; **HRMS** (ESI) *m/z* MH^+, found 231.1127. $C_{13}H_{14}N_2O_2^+$ requires 231.1128.

1-(Hydroxymethyl)-7-methoxy-2,3-dihydropyrrolo[1,2-*a*]quinazolin-5(1*H*)-one (6e).

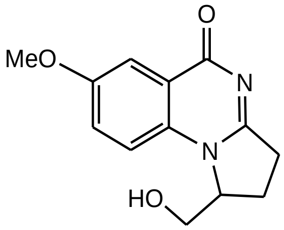

Prepared following the GP3 using quinazoline **7e** (150 mg, 0.65 mmol, 1 equiv). White solid (121 mg, 0.49 mmol, 75%). Mp: 269–271 °C; **^{1}H NMR** (400 MHz, DMSO-d_6) δ 7.57 (s, 1H, 1ArH), 7.53 (d, J = 9.8 Hz, 1H, ArH), 7.37 (d, J = 9.8 Hz, 1H, 1ArH), 4.94–4.85 (m, 2H, OH+CH), 3.89 (s, 3H, OCH_3), 3.86–3.80(m, 1H, CH_2), 3.69–3.62 (m, 1H, CH_2), 3.23-3.16 (m, 1H, CH_2), 2.87–2.81 (m, 1H, CH_2), 2.44–2.33 (m, 1H, CH_2), 2.22-2.17 (m, 1H, CH_2); **^{13}C NMR** (125 MHz, DMSO-d_6) δ 169.0, 166.0, 156.8, 132.6, 122.3, 120.0, 118.0, 108.2, 62.3, 61.4, 55.6, 32.0, 22.7; **IR/cm^{-1}**: 3225, 2960, 2937, 1634, 1615, 1547, 1508, 1069, 847; **HRMS** (ESI) *m/z* MH^+, found 247.1077. $C_{13}H_{14}N_2O_3$ requires$^+$ 247.1077.

7-Chloro-1-(hydroxymethyl)-2,3-dihydropyrrolo[1,2-*a*]quinazolin-5(1*H*)-one (6f).

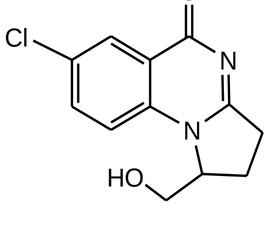

Prepared following the GP3 using quinazoline **7f** (153 mg, 0.65 mmol, 1 equiv). White solid (133 mg, 0.53 mmol, 81%). Mp: 281–283 (decom.) °C; **^{1}H NMR** (400 MHz, DMSO-d_6) δ 8.00

(s, 1H, 1ArH), 7.80 (d, *J* = 8.8 Hz, 1H, ArH), 7.67 (d, *J* = 8.8 Hz, 1H, 1ArH), 4.96 (t, *J* = 5.2 Hz, 1H, OH), 4.93–4.90 (m, 1H, CH), 3.87–3.80 (m, 1H, CH_2), 3.67–3.60 (m, 1H, CH_2), 3.25–3.20 (m, 1H, CH_2), 2.90–2.83 (m, 1H, CH_2), 2.45–2.34 (m, 1H, CH_2), 2.22-2.16 (m, 1H, CH_2); **^{13}C NMR** (150 MHz, DMSO-d_6) δ 168.5, 168.2, 137.5, 133.8, 130.0, 126.7, 120.4, 119.3, 62.9, 61.8, 32.6, 23.1; **IR/cm^{-1}**: 3260, 2928, 1638, 1599, 1538, 1492, 1161, 1084, 846; **HRMS** (ESI) *m/z* MH$^+$, found 251.0581. $C_{12}H_{11}ClN_2O_2$ requires$^+$ 251.0582.

1-(Hydroxymethyl)-7-nitro-2,3-dihydropyrrolo[1,2-*a*]quinazolin-5(1*H*)-one (6g).

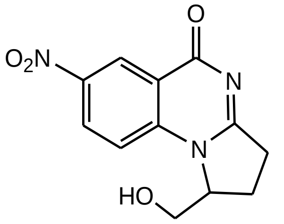

Prepared following the GP3 using quinazoline **7g** (159 mg, 0.65 mmol, 1 equiv). White solid (128 mg, 0.49 mmol, 75%). Mp: 285–287 (decom.) °C; **^{1}H NMR** (400 MHz, DMSO-d_6) δ 8.75 (s, 1H, 1ArH), 8.54 (d, *J* = 9.2 Hz, 1H, ArH), 7.86 (d, *J* = 9.2 Hz, 1H, 1ArH), 5.08 (t, *J* = 6.2 Hz, 1H, OH), 5.01–4.99 (m, 1H, CH), 3.89–3.84 (m, 1H, CH_2), 3.68–3.62 (m, 1H, CH_2), 3.26–3.20 (m, 1H, CH_2), 2.95–2.88 (m, 1H, CH_2), 2.42–2.37 (m, 1H, CH_2), 2.22–2.16 (m, 1H, CH_2); **^{13}C NMR** (125 MHz, DMSO-d_6) δ 169.9, 168.7, 144.4, 143.0, 128.2, 128.2, 123.9, 118.8, 63.3, 61.9, 32.7, 23.1; **HRMS** (ESI) *m/z* MH$^+$, found 262.0821. $C_{12}H_{11}N_3O_4$ requires$^+$ 262.0823.

1-(Hydroxymethyl)-7,8-dimethoxy-2,3-dihydropyrrolo[1,2-*a*]quinazolin-5(1*H*)-one (6h).

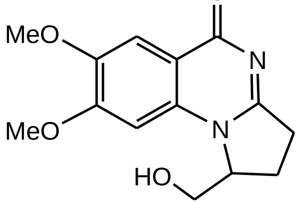

Prepared following the GP3 using quinazoline **7h** (169 mg, 0.65 mmol, 1 equiv). White solid (135 mg, 0.49 mmol, 76%). Mp: 277–279 (decom.) °C; **^{1}H NMR** (400 MHz, DMSO-d_6) δ 7.43

(s, 1H, 1ArH), 7.02 (s, 1H, 1ArH), 5.04 (t, *J* = 4.6 Hz, 1H, OH), 4.98–4.93 (m, 1H, CH), 3.92–3.84 (m, 7H, $2CH_3$ + CH_2), 3.68–3.61 (m, 1H, CH_2), 3.23–3.12 (m, 1H, CH_2), 2.84-2.78 (m, 1H, CH_2), 2.41–2.30 (m, 1H, CH_2), 2.20–2.14 (m, 1H, CH_2); **^{13}C NMR** (125 MHz, DMSO-*d*6) δ 168.7, 165.6, 153.8, 147.4, 133.8, 112.1, 107.4, 98.5, 62.2, 61.5, 56.4, 55.7, 31.9, 22.6; **HRMS** (ESI) *m/z* MH^+, found 277.1183. $C_{14}H_{16}N_2O_4^+$ requires 277.1183.

8-Chloro-1-(hydroxymethyl)-2,3-dihydropyrrolo[1,2-*a*]quinazolin-5(1*H*)-one (6i).

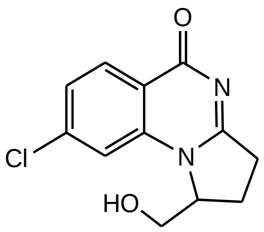

Prepared following the GP3 using quinazoline **7i** (153 mg, 0.65 mmol, 1 equiv). White solid (130 mg, 0.52 mmol, 80%). Mp: 222–224 (decom.) °C; **^{1}H NMR** (400 MHz, DMSO–*d*6) δ 8.05 (d, *J* = 8.4 Hz, 1H, 1ArH), 7.79 (d, *J* = 1.6 Hz, 1H, ArH), 7.49 (dd, J^1 = 8.4 Hz, J^2 = 1.6 Hz, 1H, 1ArH), 5.04 (t, *J* = 4 Hz, 1H, OH), 4.94–4.92 (m, 1H, CH), 3.86–3.79 (m, 1H, CH_2), 3.63–3.57 (m, 1H, CH_2), 3.24–3.15 (m, 1H, CH_2), 2.88–2.81 (m, 1H, CH_2), 2.42-2.32 (m, 1H, CH_2), 2.18–2.12 (m, 1H, CH_2); **^{13}C NMR** (125 MHz, DMSO-*d*6) δ 168.9, 168.5, 139.8, 138.6, 130.1, 126.0, 117.7, 116.4, 62.8, 61.9, 32.6, 23.2; **HRMS** (ESI) *m/z* MH^+, found 251.0582. $C_{12}H_{11}ClN_2O_2$ requires$^+$ 251.0582.

1-(Hydroxymethyl)-9-methyl-2,3-dihydropyrrolo[1,2-*a*]quinazolin-5(1*H*)-one (6j).

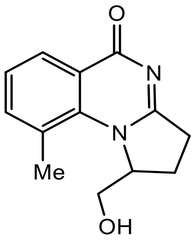

Prepared following the GP3 using quinazoline **7j** (139 mg, 0.65 mmol, 1 equiv). White solid (122 mg, 0.53 mmol, 81%). Mp: 217-219 (decom.) °C; **^{1}H NMR** (400 MHz, DMSO-*d*6) δ 8.00 (d, *J* = 7.6 Hz, 1H, 1ArH), 7.58 (d, *J* = 7.6 Hz, 1H, ArH), 7.35 (t, *J* = 7.6 Hz, 1H, 1ArH), 5.36–

5.13 (m, 1H, CH), 5.09 (t, *J* = 5.6 Hz, 1H, OH), 3.60–3.47 (m, 2H, CH_2), 3.17–3.07 (m, 1H, CH_2), 2.92–2.85 (m, 1H, CH_2), 2.65 (s, 3H, CH_3), 2.40–2.29 (m, 1H, CH_2), 2.14–2.09 (m, 1H, CH_2); **^{13}C NMR** (125 MHz, DMSO-*d*$_6$) δ 169.3, 168.6, 138.1, 137.5, 126.4, 125.7, 125.5, 120.5, 64.7, 62.6, 31.5, 22.2, 21.5; **IR/cm^{-1}**: 3213, 2926, 2872, 1637, 1552, 1493, 1413, 1329, 1086, 1015, 760; **HRMS** (ESI) *m/z* MH$^+$, found 231.1125. $C_{13}H_{14}N_2O_2$+ requires$^+$ 231.1128.

9-Bromo-1-(hydroxymethyl)-2,3-dihydropyrrolo[1,2-*a*]quinazolin-5(1*H*)-one (6k).

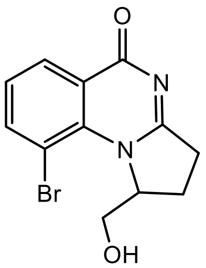

Prepared following the GP3 using quinazoline **7k** (181 mg, 0.65 mmol, 1 equiv). White solid (144 mg, 0.49 mmol, 76%). Mp: 227-229 °C; **^{1}H NMR** (400 MHz, DMSO-*d*$_6$) δ 8.17 (dd, *J* = 7.8 Hz, *J* = 1.6 Hz, 1H, 1ArH), 8.08 (dd, *J* = 7.8 Hz, *J* = 1.6 Hz, 1H, ArH), 7.38 (t, *J* = 7.8 Hz, 1H, 1ArH), 6.05–6.03 (m, 1H, CH), 5.07 (t, *J* = 5.4 Hz, 1H, OH), 3.72–3.63 (m, 2H, CH_2), 3.18–3.09 (m, 1H, CH_2), 2.94–2.87 (m, 1H, CH_2), 2.42–2.31 (m, 1H, CH_2), 2.17–2.11 (m, 1H, CH_2); **^{13}C NMR** (100 MHz, DMSO-*d*$_6$) δ 170.4, 168.6, 141.0, 137.6, 128.9, 127.3, 122.6, 108.0, 64.9, 63.3, 32.4, 21.6; **HRMS** (ESI) *m/z* MH$^+$, found 295.0075. $C_{12}H_{11}BrN_2O_2^+$ requires 295.0077.

9-Fluoro-1-(hydroxymethyl)-2,3-dihydropyrrolo[1,2-*a*]quinazolin-5(1*H*)-one (6l).

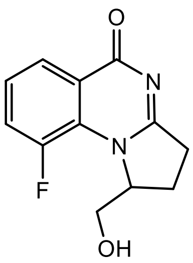

Prepared following the GP3 using quinazoline **7l** (142 mg, 0.65 mmol, 1 equiv). White solid (124 mg, 0.53 mmol, 82%). Mp: 239-241 °C; **^{1}H NMR** (400 MHz, DMSO-d_6) δ 7.93 (d, J = 8 Hz, 1H, 1ArH), 7.73-7.67 (m, 1H, ArH), 7.49-7.44 (m, 1H, 1ArH), 5.09 (t, J = 5.6 Hz, 1H, OH), 5.04–5.01 (m, 1H, CH), 3.75-3.64 (m, 2H, CH_2), 3.19–3.10 (m, 1H, CH_2), 2.88-2.81 (m, 1H, CH_2), 2.43-2.32 (m, 1H, CH_2), 2.20–2.14 (m, 1H, CH_2); **^{19}F NMR** (188 MHz, DMSO-d_6) δ -126.9 (m, 1F); **^{13}C NMR** (125 MHz, DMSO-d_6) δ 168.5, 167.9, 149.9 (d, $^{1}J_{C\text{-}F}$ = 247.5 Hz), 127.5 (d, $^{3}J_{C\text{-}F}$ = 8.8 Hz), 125.9 (d, $^{3}J_{C\text{-}F}$ = 7.5 Hz), 123.8, 121.2, 120.3 (d, $^{2}J_{C\text{-}F}$ = 21.25 Hz), 62.7 (d, $^{4}J_{C\text{-}F}$ = 8.8 Hz), 62.6, 31.6, 22.4; **HRMS** (ESI) *m/z* MH^+, found 235.0878. $C_{12}H_{11}FN_2O_2^+$ requires 235.0878.

References

1 Zborovskii, Y.L.; Orysyk, V.V.; Dobosh, A.A.; Staninets, V.I.; Pirozhenko, V.V.; Chernega A.N. *Chem.Heterocycl.Compd.* **2003**, *39*, 1099-1106.

2 Sheldrick, G. *Acta Cryst., Sect. A*, **2008**, *64*, 112.

3 Hamasharif, M.S.; Smith, O.E.P.; Curran, C.J.; Hemming, K. *ACS Omega* **2017**, *2*, 1222−1231. doi:10.1021/acsomega.7b00211.

4

^1H, ^{13}C and ^{19}F NMR- spectra data

Figure S3. ^1H NMR spectrum (400 MHz, $CDCl_3$) of compound 10a.

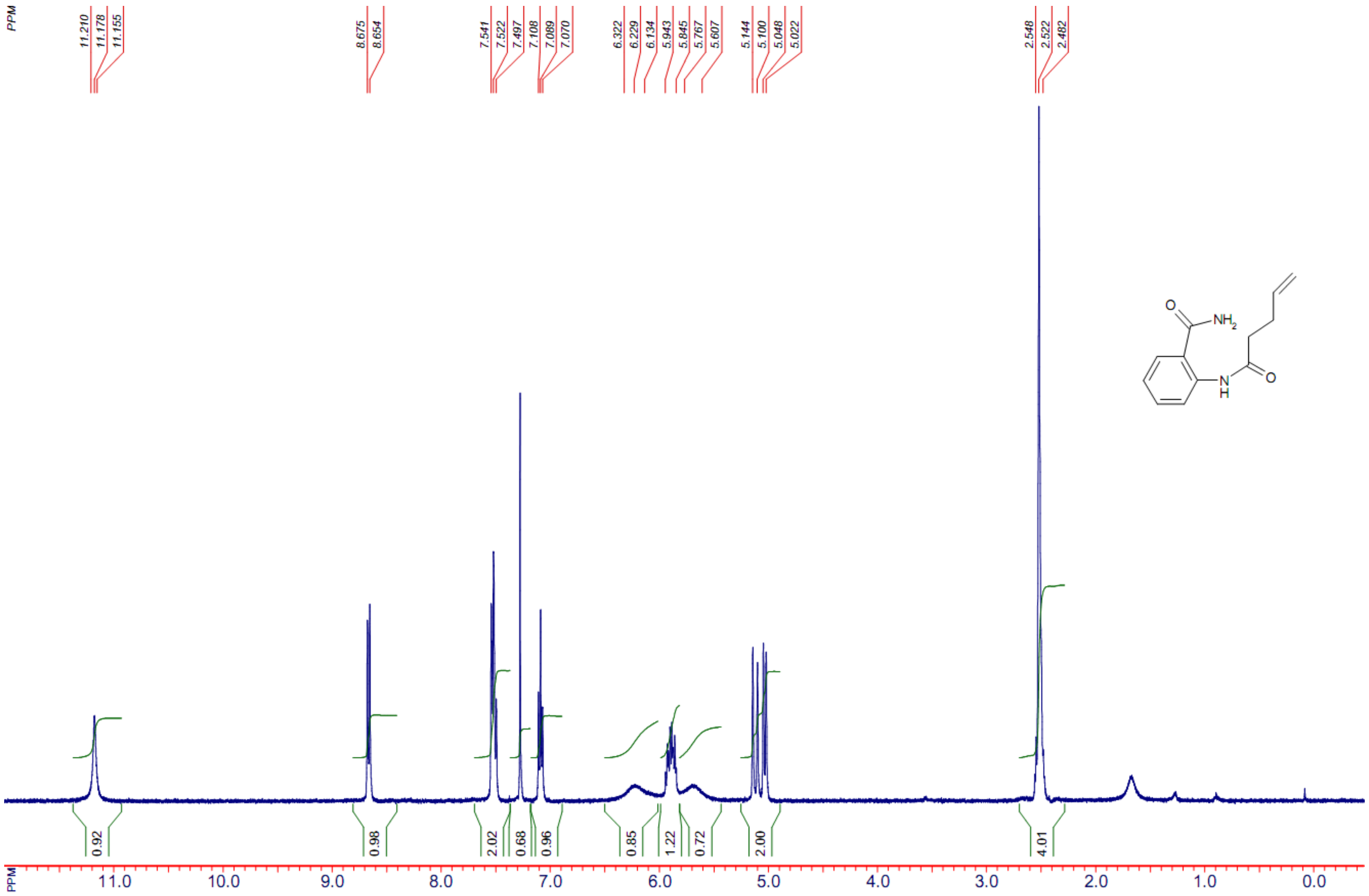

**File name: phvm45-1.fid**	Operator:	SF: 399.9736 MHz	NSC: 0	PW: 11.60 usec, RG: 30	SI: 32768
Date: 06-Jul-2020	Solvent: cdcl3	SW: 7599 Hz	TE: 294 K	AQ: 2.11 sec, RD: 0.00 sec	□

Figure S4. ^{13}C NMR spectrum (125 MHz, DMSO-d_6) of compound 10a.

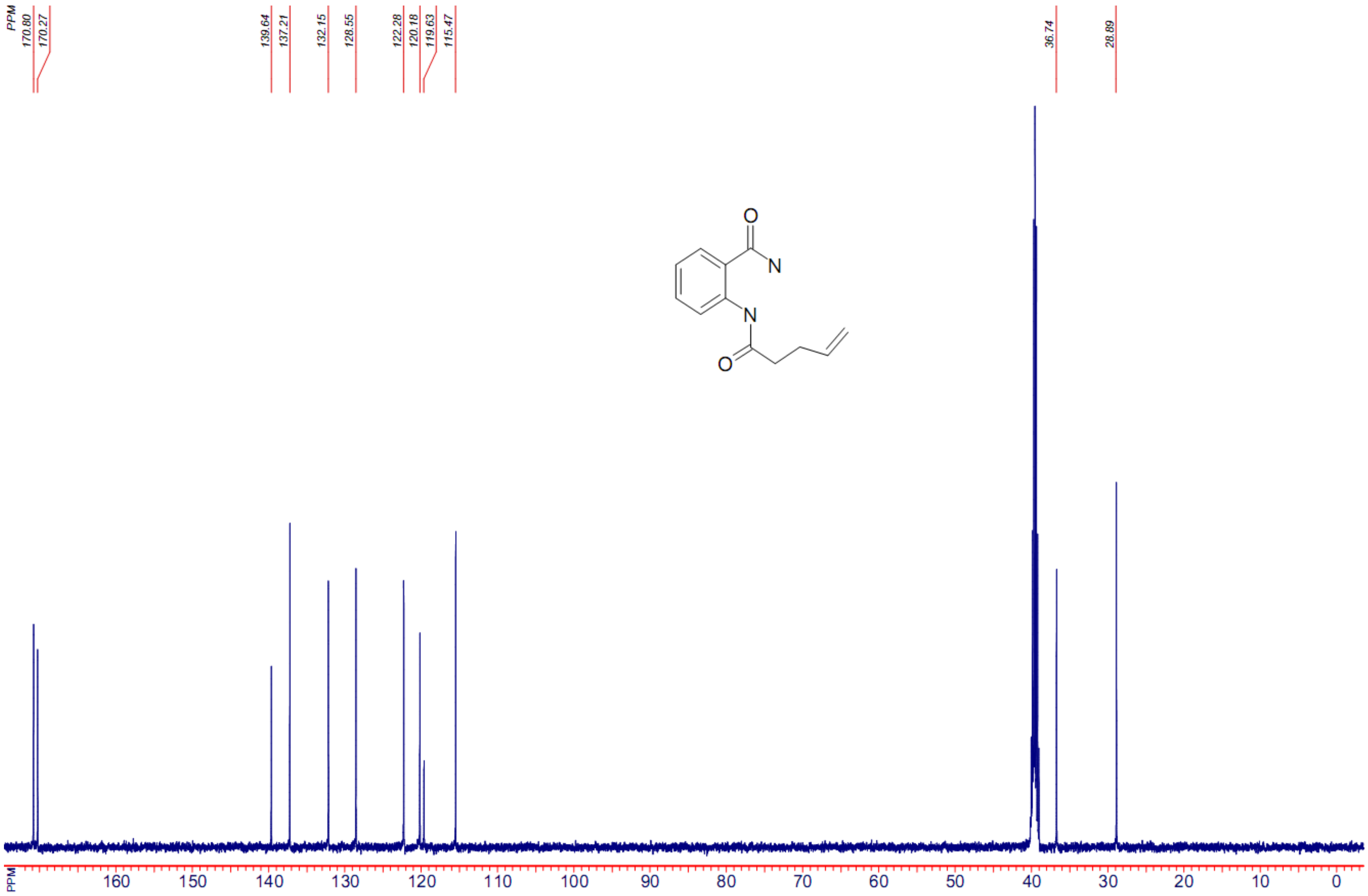

**File name: PHVM22_C13**	Operator: root	SF: 125.6681 MHz	NSC: 189	PW: 0.00 usec, RG: 51200	SI: 131072
Date: 14-Jan-2021	Solvent: CDCl3	SW: 32680 Hz	TE: 683 K	AQ: 1.57 sec, RD: 0.00 sec	

Figure S5. ^{1}H NMR spectrum (400 MHz, DMSO-d_6) of compound 10b.

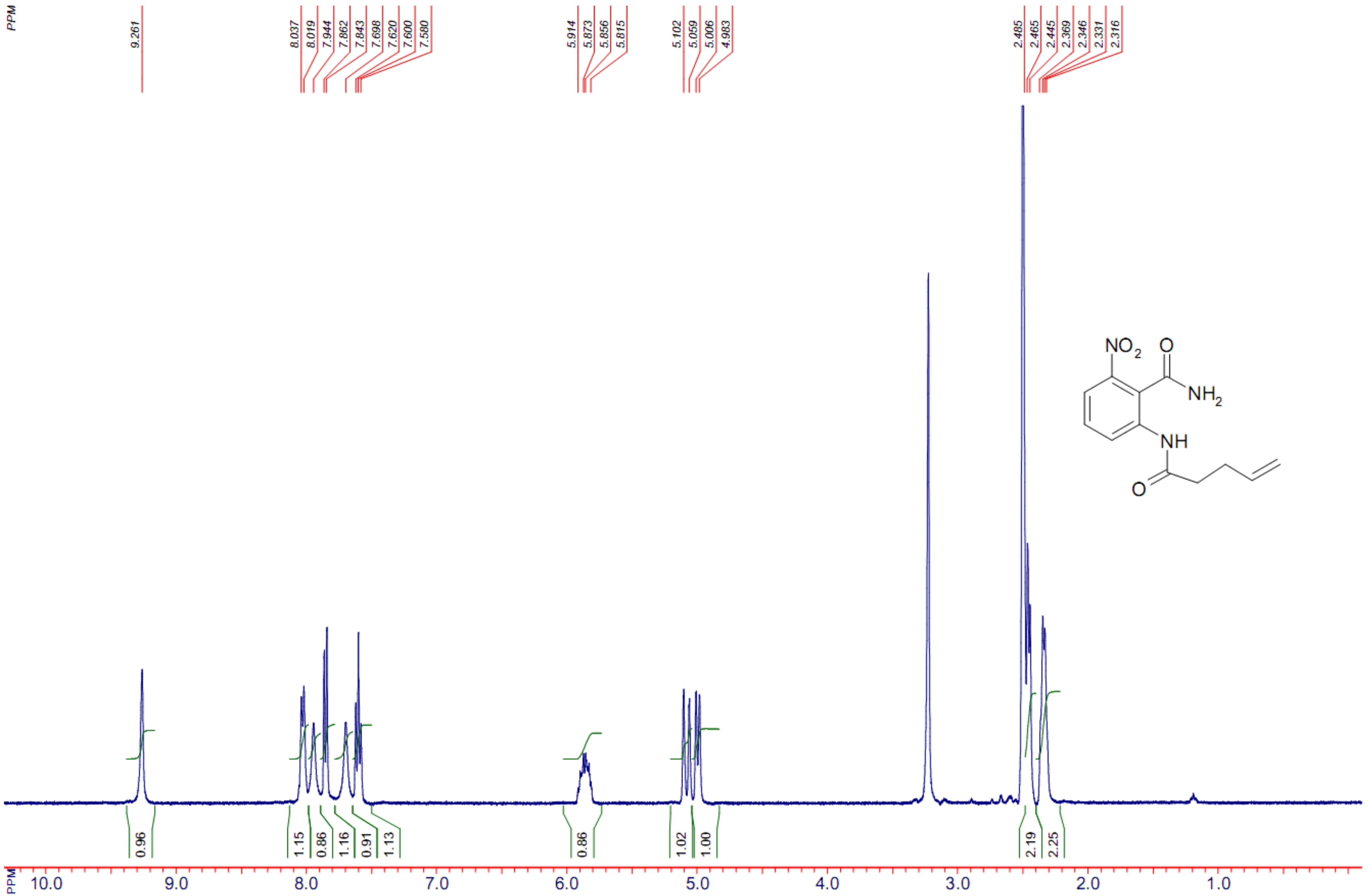

File name: PHvm86.fid	Operator:	SF: 399.9753 MHz	NSC: 0	PW: 10.90 usec, RG: 24	SI: 32768
Date: 26-Apr-2021	Solvent: dmso	SW: 8000 Hz	TE: 298 K	AQ: 2.00 sec, RD: 0.00 sec	□

Figure S6. ^{13}C NMR spectrum (150 MHz, DMSO-d_6) of compound 10b.

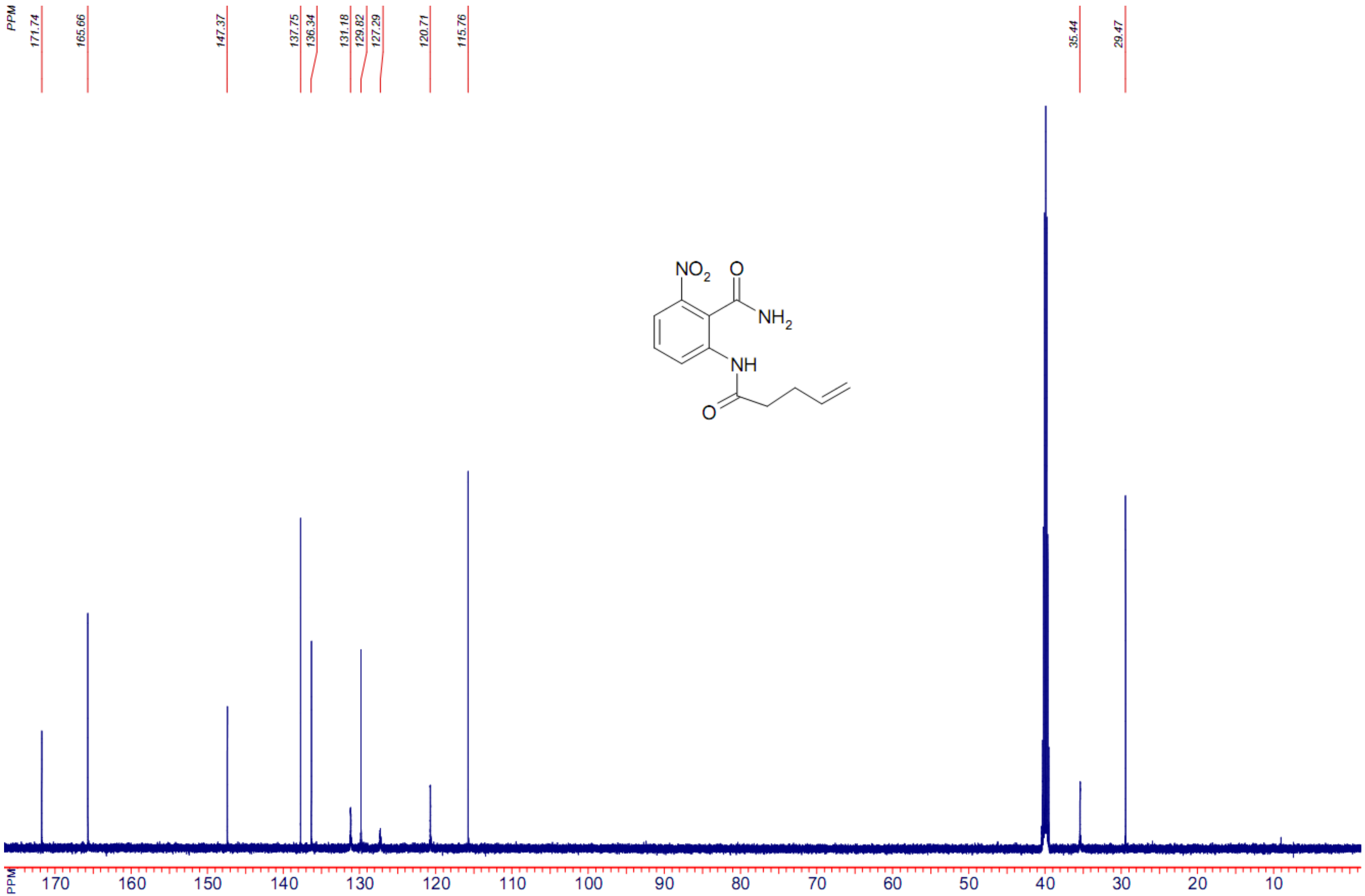

File name: phvm86_C13.fid	Operator:	SF: 150.8305 MHz	NSC: 0	PW: 3.09 usec, RG: 60	SI: 131072
Date: 28-Apr-2021	Solvent: dmso	SW: 37879 Hz	TE: 298 K	AQ: 1.73 sec, RD: 0.00 sec	□

Figure S7. ^{1}H NMR spectrum (400 MHz, DMSO-d_6) of compound 10c.

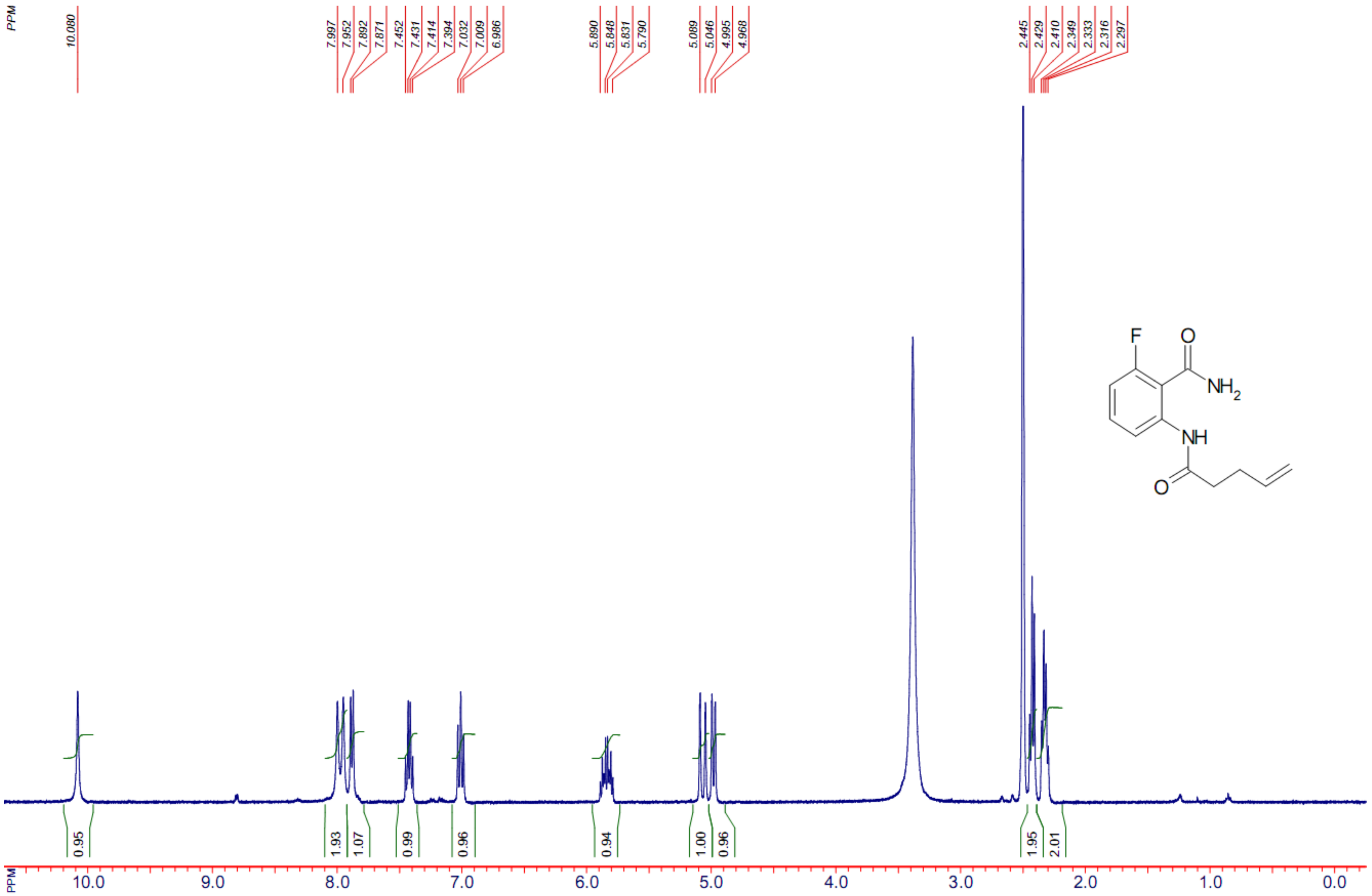

**File name: PHvm1475.fid**	Operator:	SF: 399.9753 MHz	NSC: 0	PW: 10.90 usec, RG: 24	SI: 32768
Date: 31-Mar-2021	Solvent: dmso	SW: 8000 Hz	TE: 298 K	AQ: 2.00 sec, RD: 0.00 sec	□

Figure S8. ^{13}**C NMR spectrum (125 MHz, DMSO-*d*$_6$) of compound 10c.**

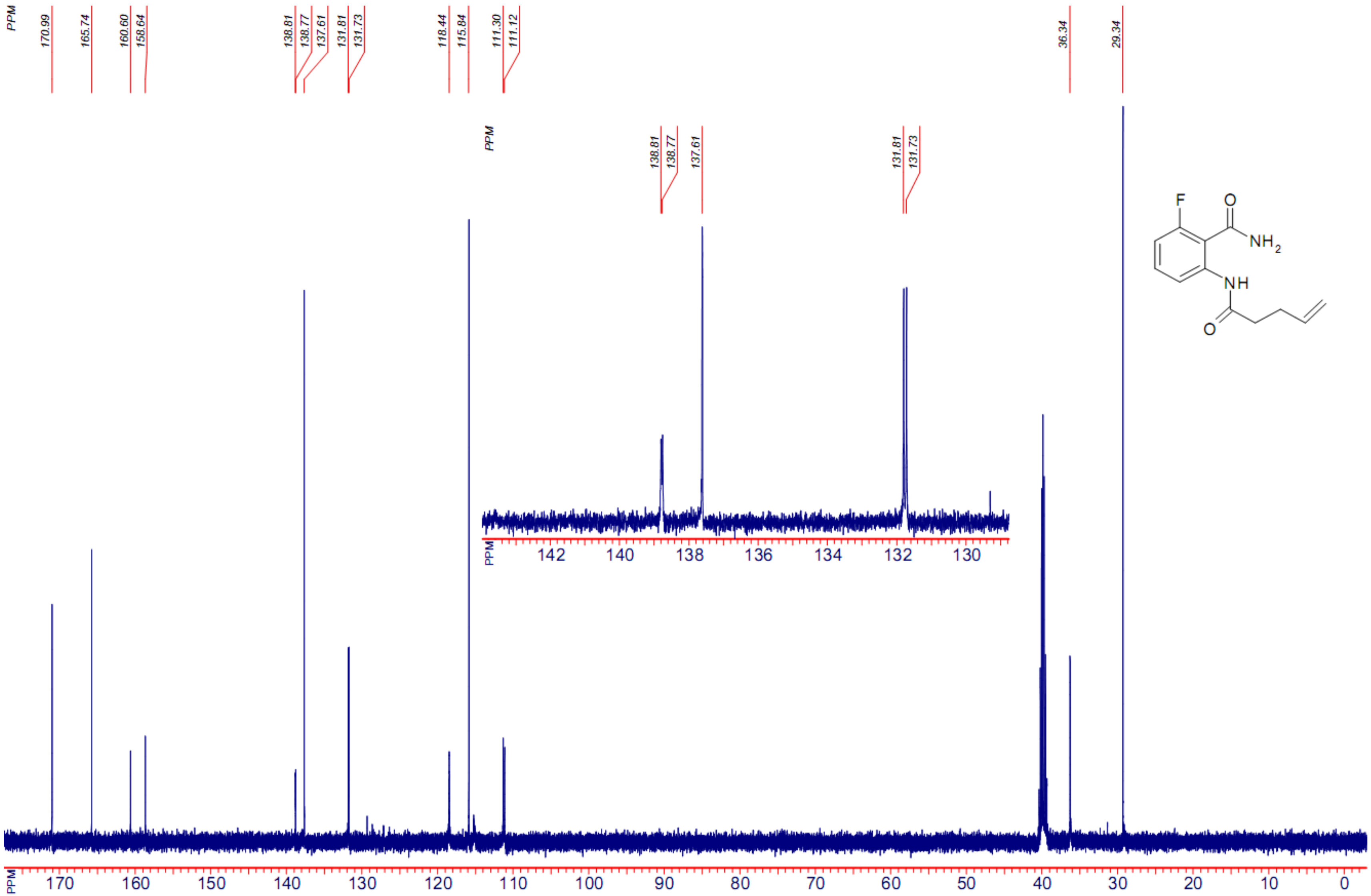

**File name: phvm1475_C13.fid**	Operator:	SF: 125.6927 MHz	NSC: 0	PW: 3.27 usec, RG: 60	SI: 65536
Date: 01-Apr-2021	Solvent: dmso	SW: 31250 Hz	TE: 298 K	AQ: 1.02 sec, RD: 0.00 sec	13C (1H-decoupled)

Figure S9. ^{1}H NMR spectrum (400 MHz, DMSO-*d*6) of compound 10d.

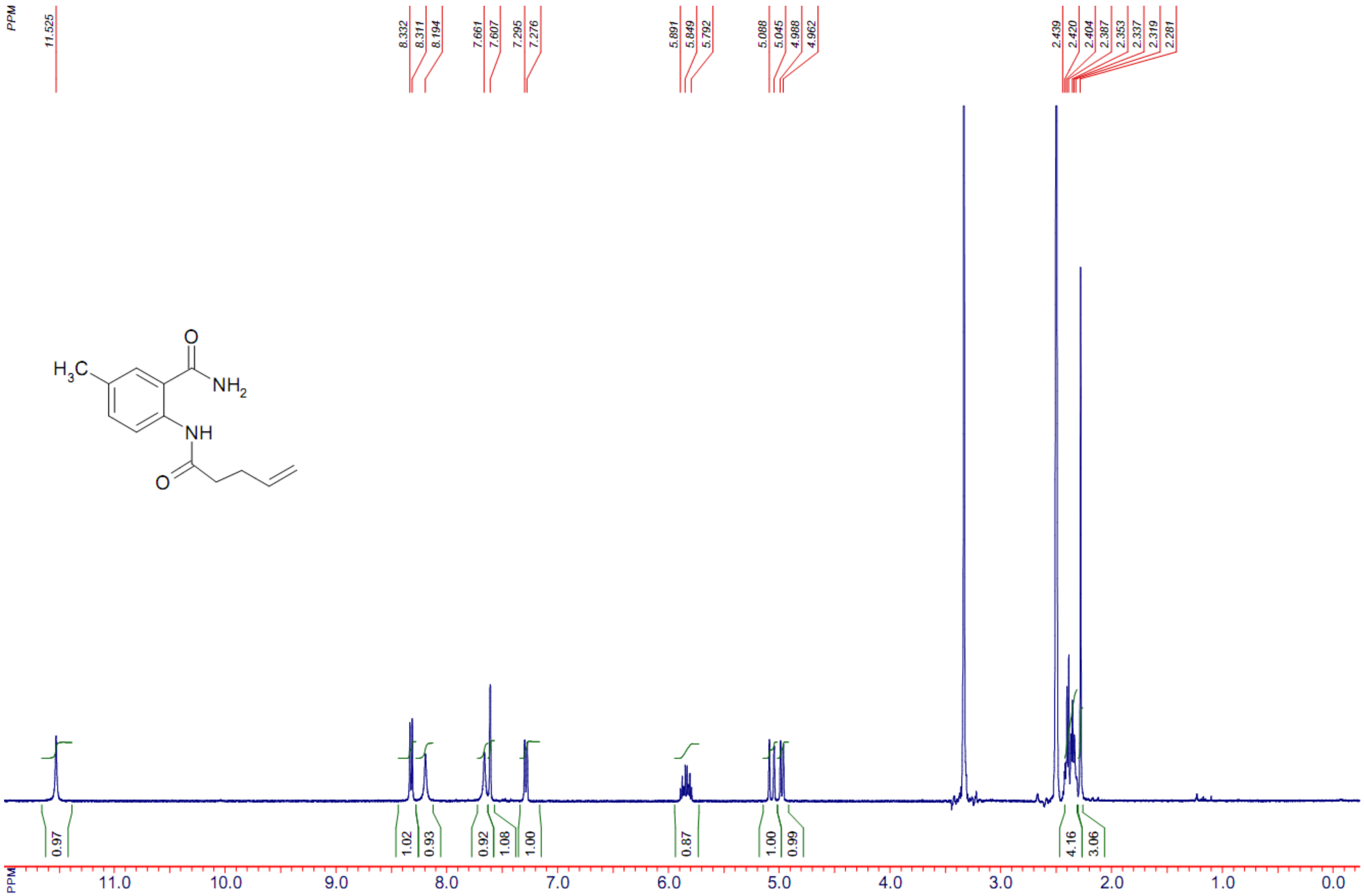

**File name: PHVM93-6.fid**	Operator:	SF: 399.9753 MHz	NSC: 0	PW: 10.90 usec, RG: 24	SI: 32768
Date: 25-May-2021	Solvent: dmso	SW: 8000 Hz	TE: 298 K	AQ: 2.00 sec, RD: 0.00 sec	□

Figure S10. ^{13}C **NMR spectrum (125 MHz, DMSO-d_6) of compound 10d.**

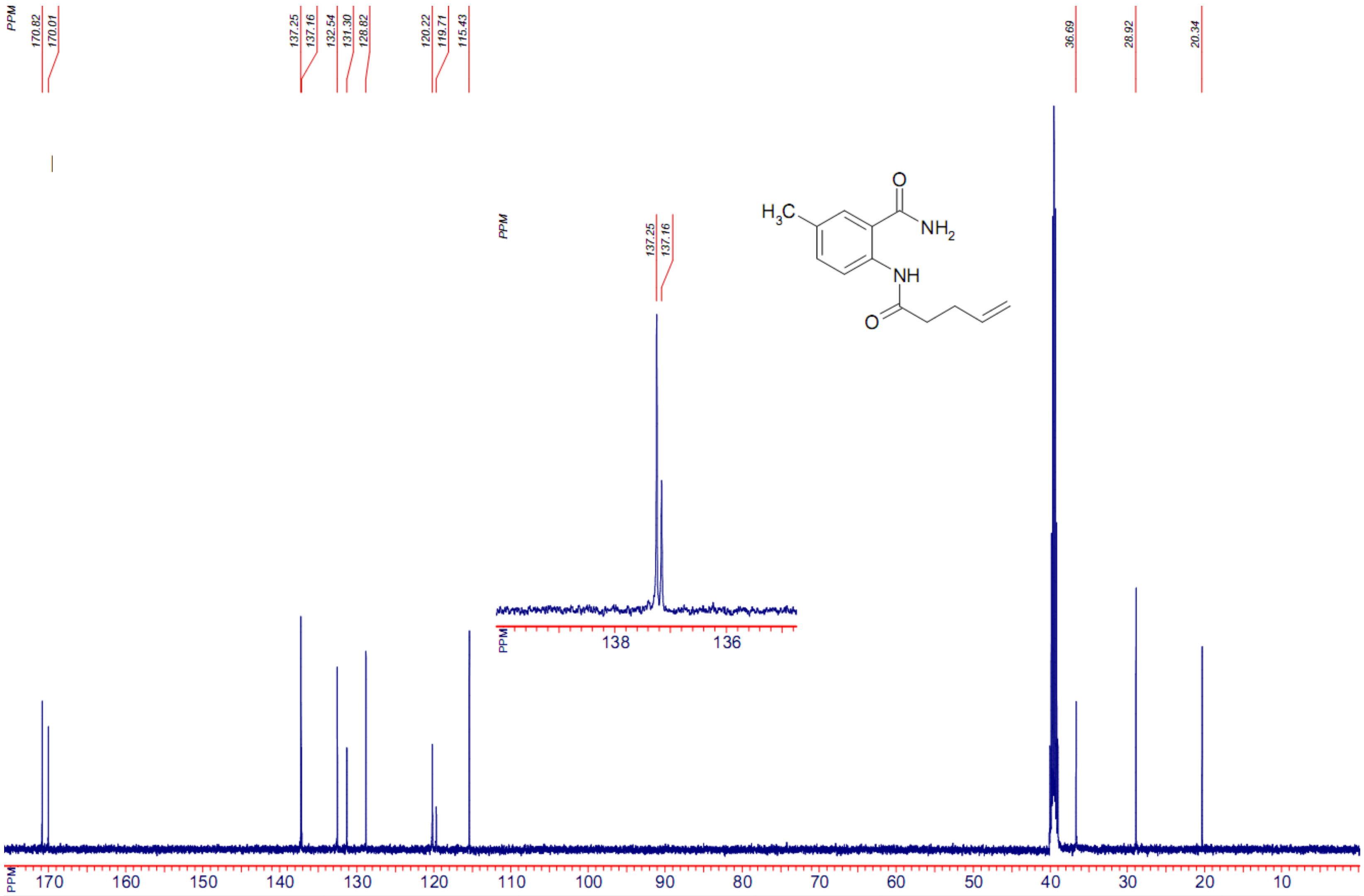

File name: PHvm93-6-C13	Operator: root	SF: 125.6681 MHz	NSC: 202	PW: 0.00 usec, RG: 51200	SI: 131072
Date: 27-May-2021	Solvent: DMSO	SW: 32680 Hz	TE: 683 K	AQ: 1.57 sec, RD: 0.00 sec	

Figure S11. ^{1}H NMR spectrum (400 MHz, DMSO-d_6) of compound 10e.

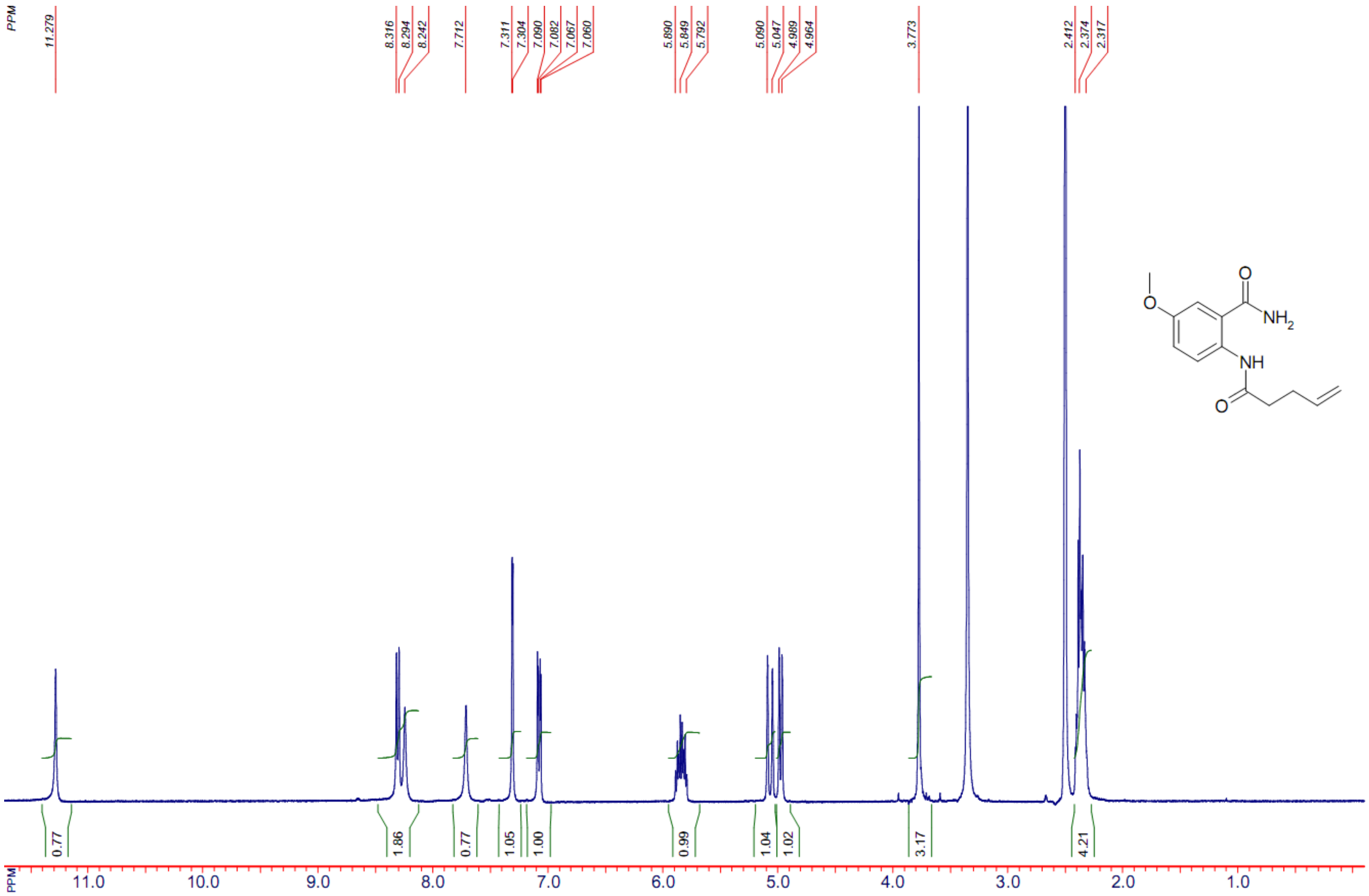

**File name: PHVM48-1.fid**	Operator:	SF: 399.9755 MHz	NSC: 0	PW: 11.60 usec, RG: 20	SI: 32768
Date: 02-Mar-2021	Solvent: dmso	SW: 7599 Hz	TE: 294 K	AQ: 2.11 sec, RD: 0.00 sec	□

Figure S12. ^{13}C **NMR spectrum (125 MHz, DMSO-*d*$_6$) of compound 10e.**

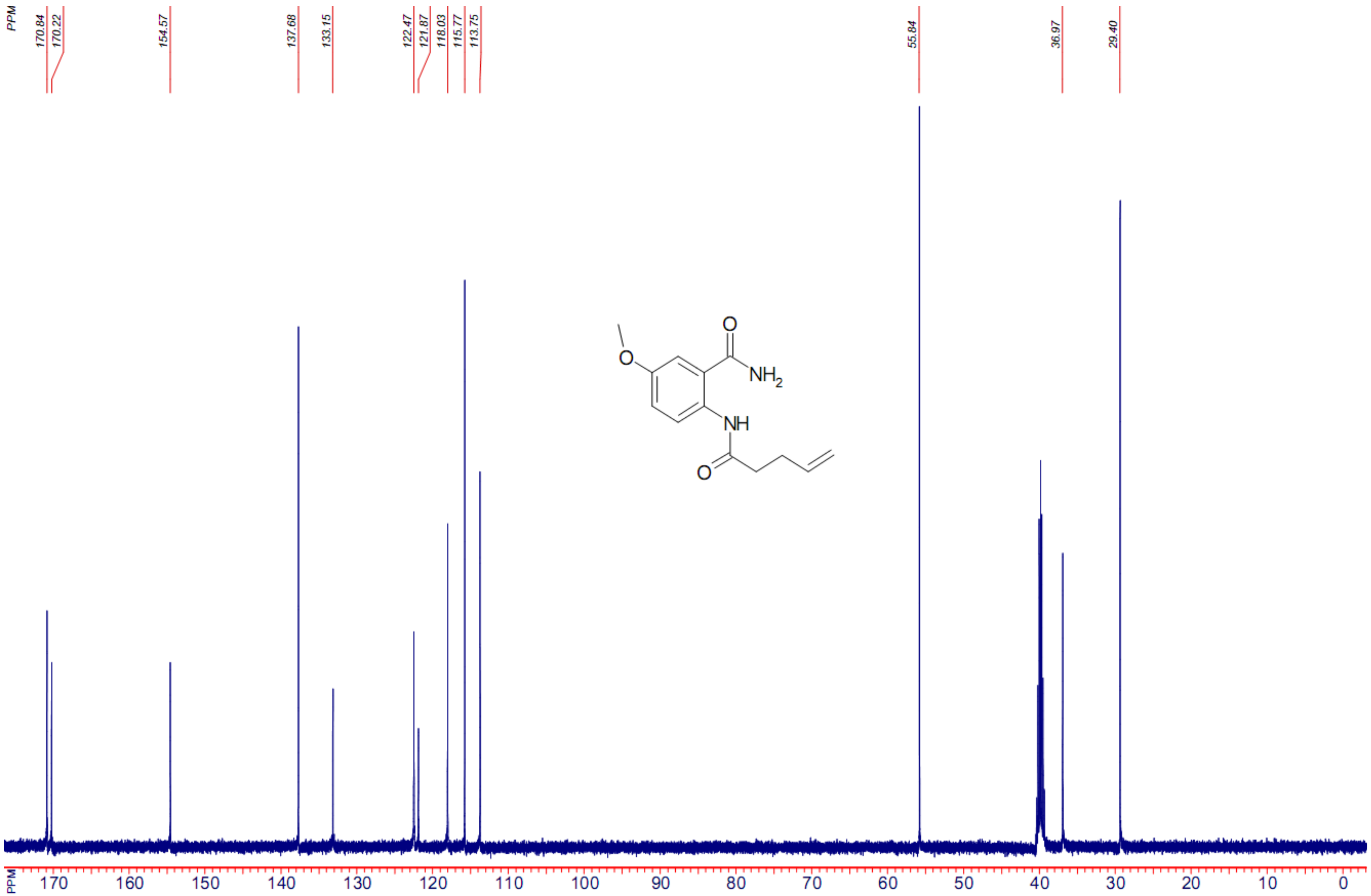

**File name: phvm48-1_C13.fid**	Operator:	SF: 125.6927 MHz	NSC: 0	PW: 3.27 usec, RG: 60	SI: 65536
Date: 04-Mar-2021	Solvent: dmso	SW: 31250 Hz	TE: 298 K	AQ: 1.02 sec, RD: 0.00 sec	13C (1H-decoupled)

Figure S13. ^{1}H NMR spectrum (400 MHz, DMSO-d_6) of compound **10f**.

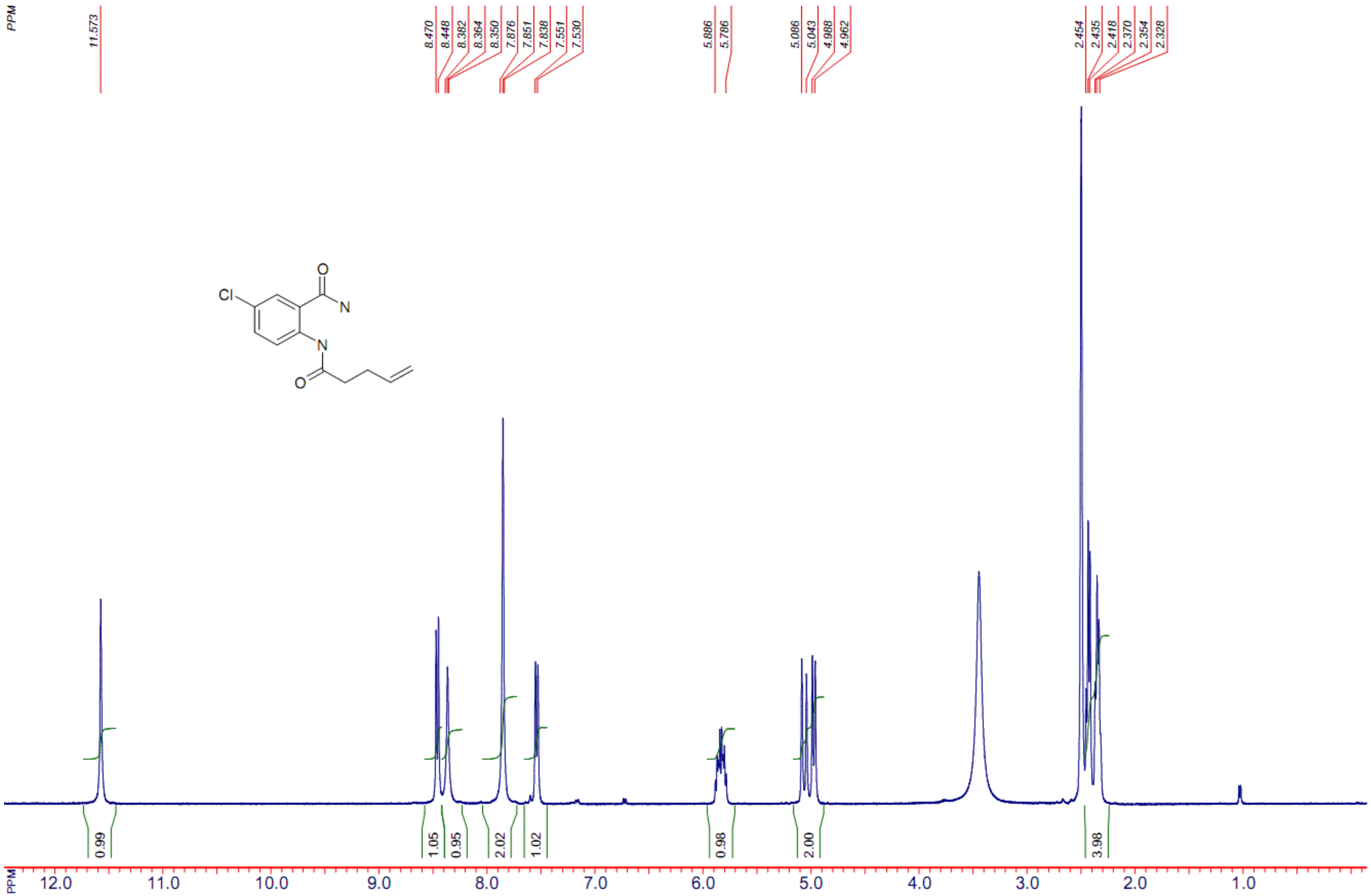

**File name: phvm4-5.fid**	Operator:	SF: 399.9755 MHz	NSC: 0	PW: 11.60 usec, RG: 20	SI: 32768
Date: 16-Nov-2020	Solvent: dmso	SW: 7599 Hz	TE: 294 K	AQ: 2.11 sec, RD: 0.00 sec	□

Figure S14. ^{13}C NMR spectrum (150 MHz, DMSO-d_6) of compound 10f.

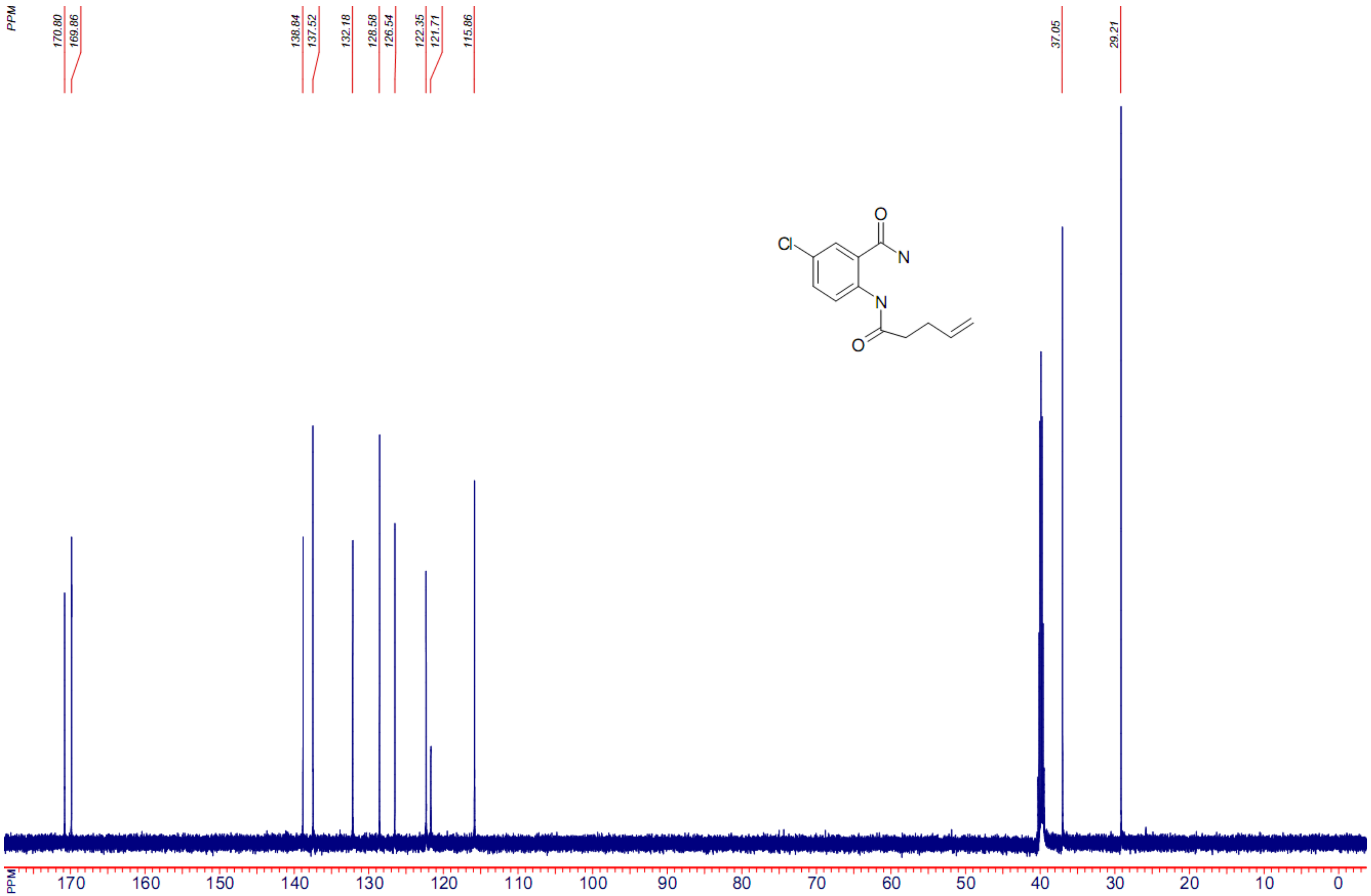

File name: phvm4_C13.fid	Operator:	SF: 150.8304 MHz	NSC: 0	PW: 3.09 usec, RG: 60	SI: 131072
Date: 17-Nov-2020	Solvent: dmso	SW: 37879 Hz	TE: 298 K	AQ: 1.69 sec, RD: 0.00 sec	□

Figure S15. ^{1}H NMR spectrum (400 MHz, DMSO-d_6) of compound **10g**.

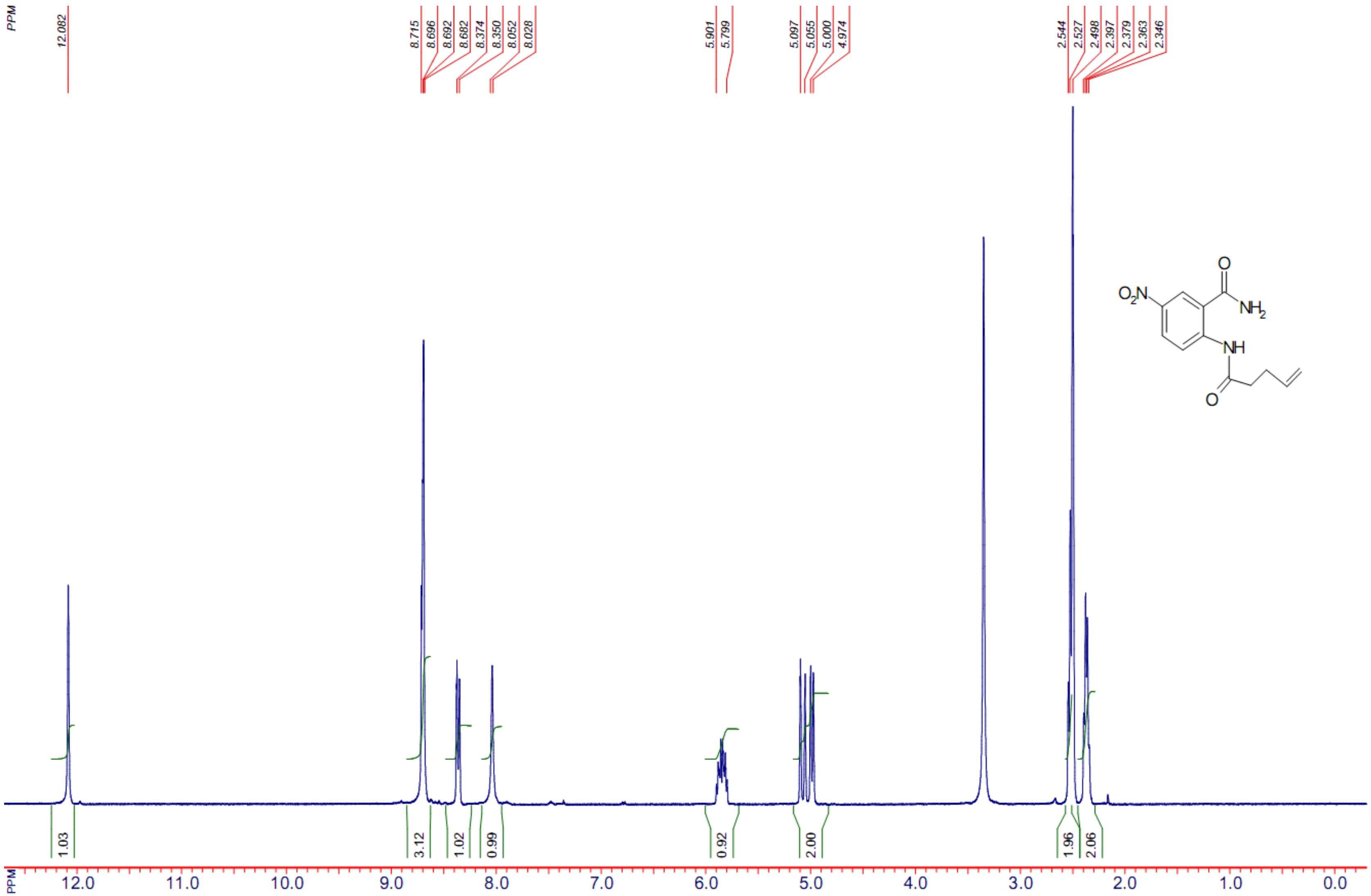

**File name: PHvm16-1.fid**	Operator:	SF: 399.9755 MHz	NSC: 0	PW: 11.60 usec, RG: 30	SI: 32768
Date: 02-Dec-2020	Solvent: dmso	SW: 7599 Hz	TE: 294 K	AQ: 2.11 sec, RD: 0.00 sec	□

Figure S16. **^{13}C NMR spectrum (125 MHz, DMSO-*d*6) of compound 10g.**

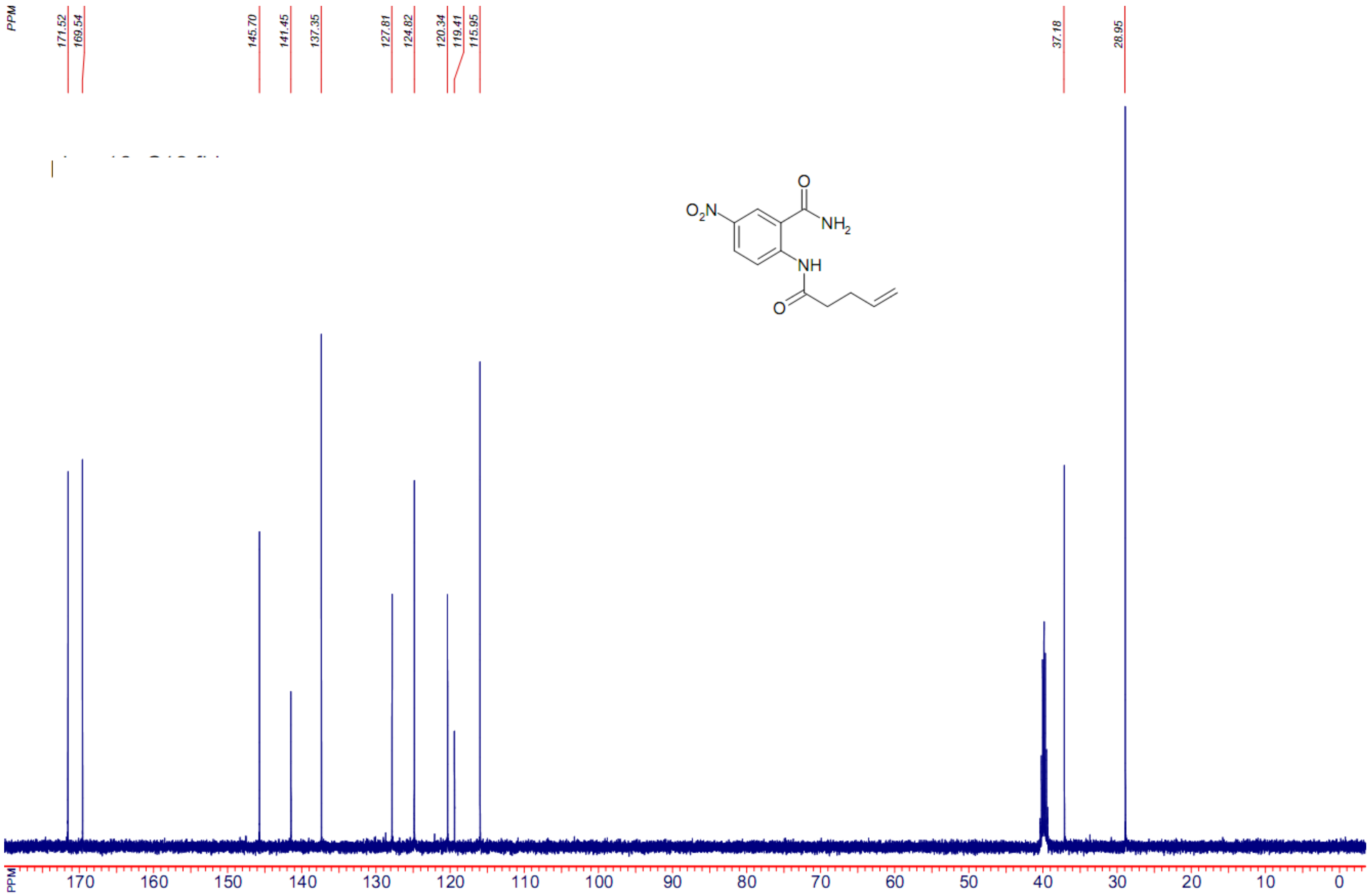

**File name: phvm16_C13.fid**	Operator:	SF: 125.6927 MHz	NSC: 0	PW: 3.27 usec, RG: 60	SI: 65536
Date: 04-Dec-2020	Solvent: dmso	SW: 31250 Hz	TE: 298 K	AQ: 1.02 sec, RD: 0.00 sec	□

Figure S17. ^{1}H NMR spectrum (400 MHz, DMSO-d_6) of compound 10h.

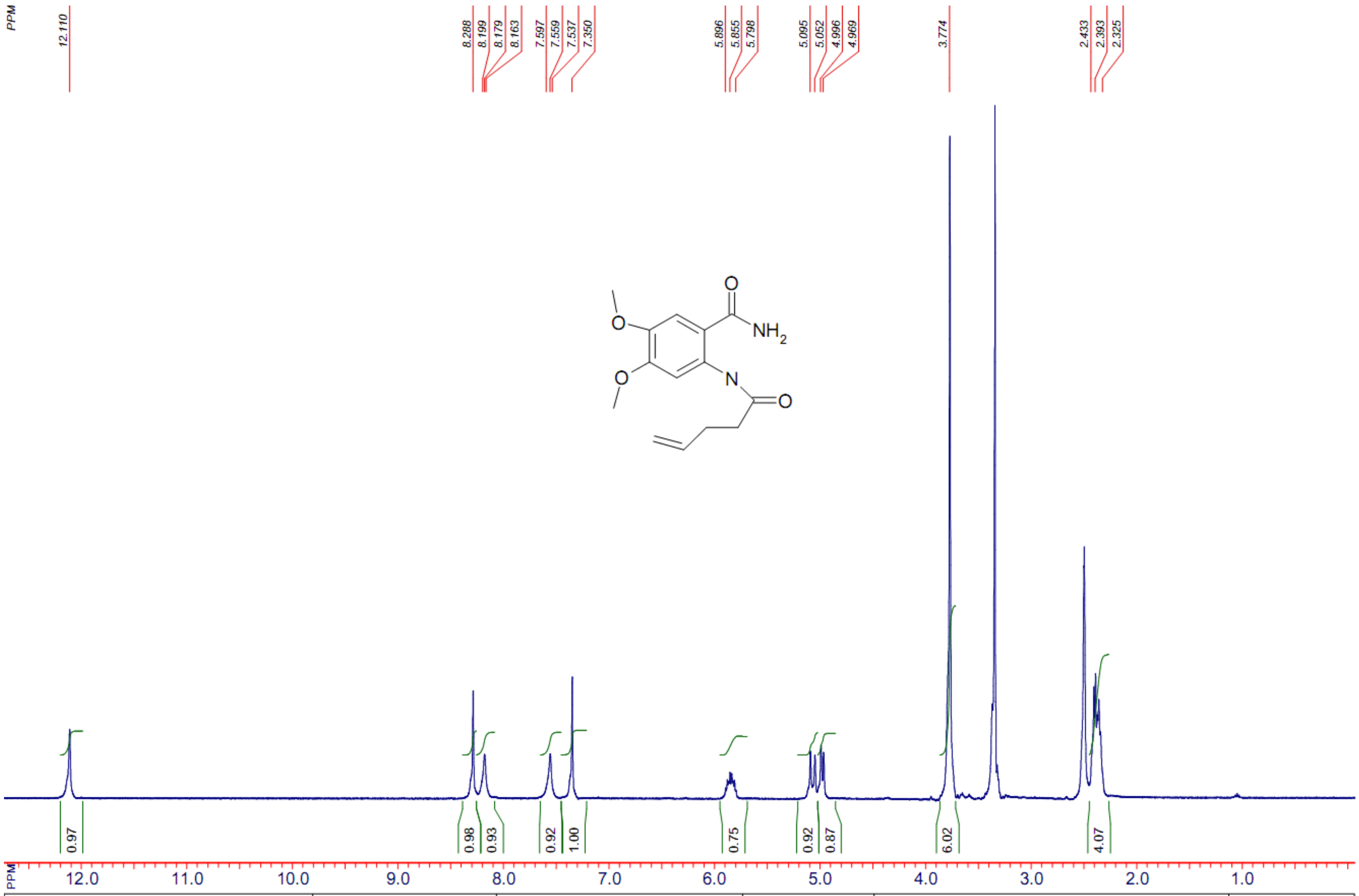

**File name: phvm18-2.fid**	Operator:	SF: 399.9755 MHz	NSC: 0	PW: 11.60 usec, RG: 20	SI: 32768
Date: 14-Dec-2020	Solvent: dmso	SW: 7599 Hz	TE: 294 K	AQ: 2.11 sec, RD: 0.00 sec	□

Figure S18. ^{13}**C NMR spectrum (150 MHz, DMSO-*d*$_6$) of compound 10h.**

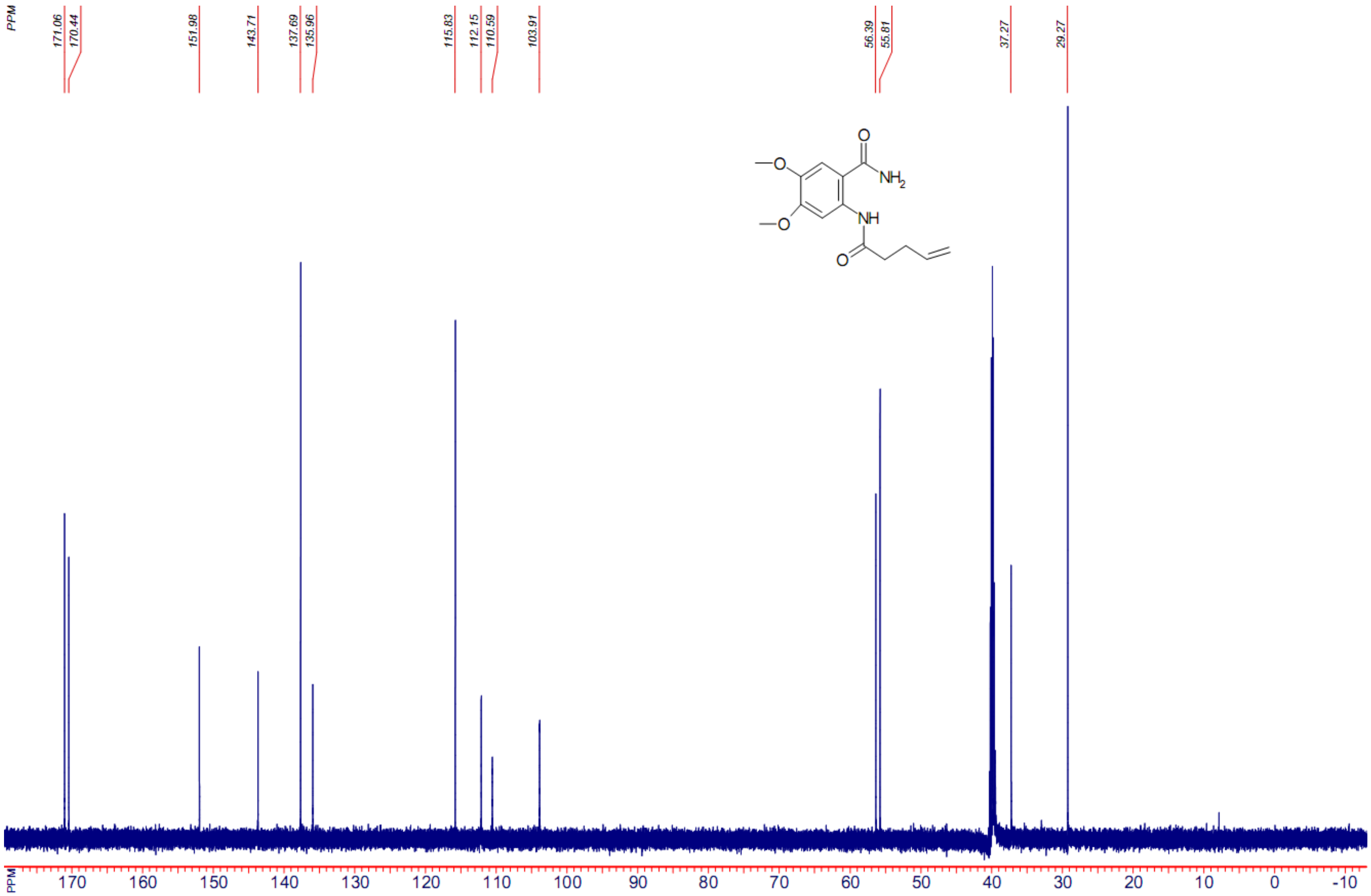

File name: PHVM18-2_C13.fid	Operator:	SF: 150.8304 MHz	NSC: 0	PW: 3.09 usec, RG: 60	SI: 131072
Date: 14-Dec-2020	Solvent: dmso	SW: 37879 Hz	TE: 298 K	AQ: 1.69 sec, RD: 0.00 sec	□

Figure S19. [1]**H NMR spectrum (400 MHz, DMSO-*d*6) of compound 10i.**

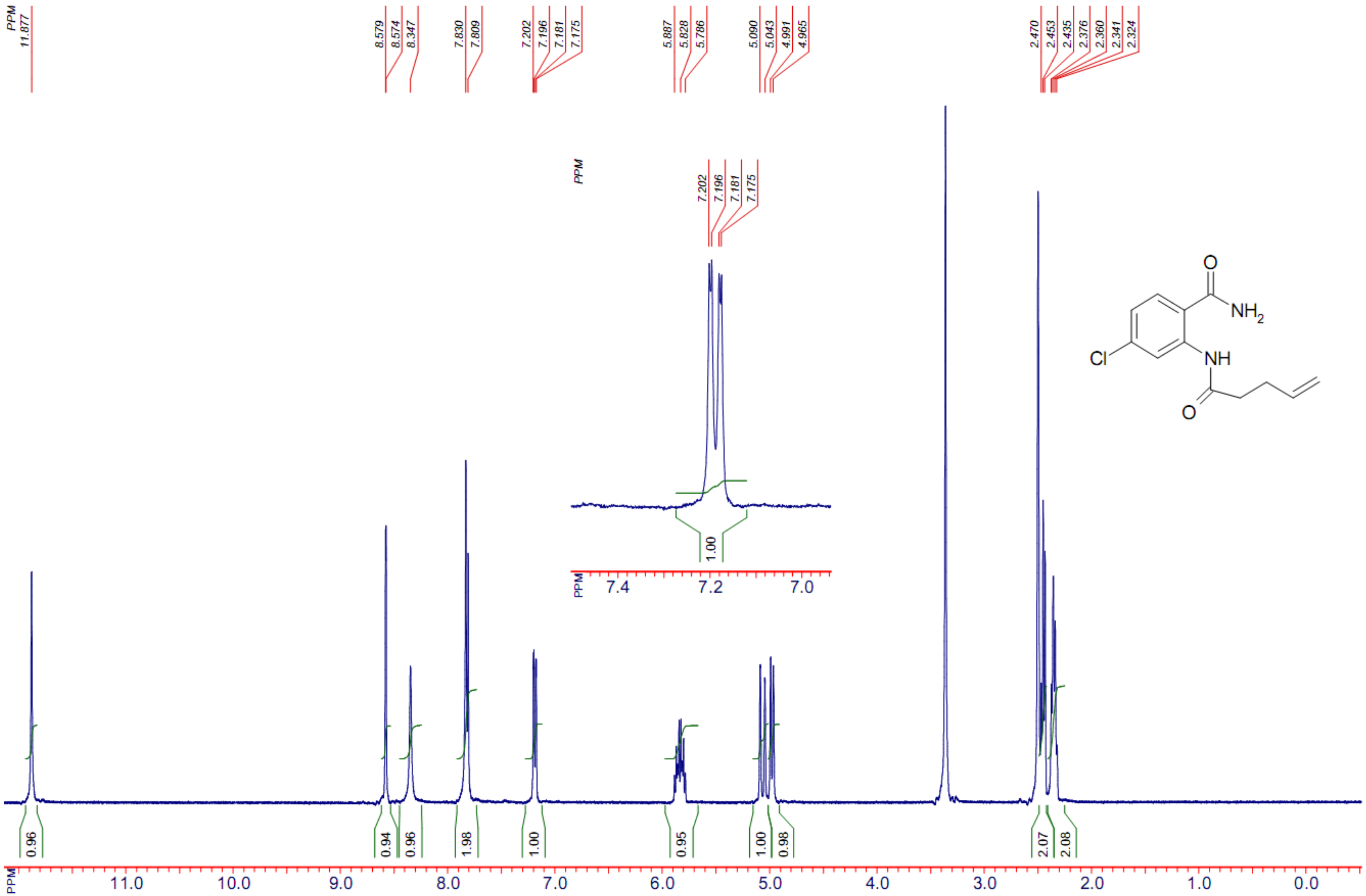

File name: PHvm1481.fid	Operator:	SF: 399.9753 MHz	NSC: 0	PW: 10.90 usec, RG: 34	SI: 32768
Date: 07-Apr-2021	Solvent: dmso	SW: 8000 Hz	TE: 298 K	AQ: 2.00 sec, RD: 0.00 sec	□

Figure S20. ^{13}C **NMR spectrum (125 MHz, DMSO-*d*$_6$) of compound 10i.**

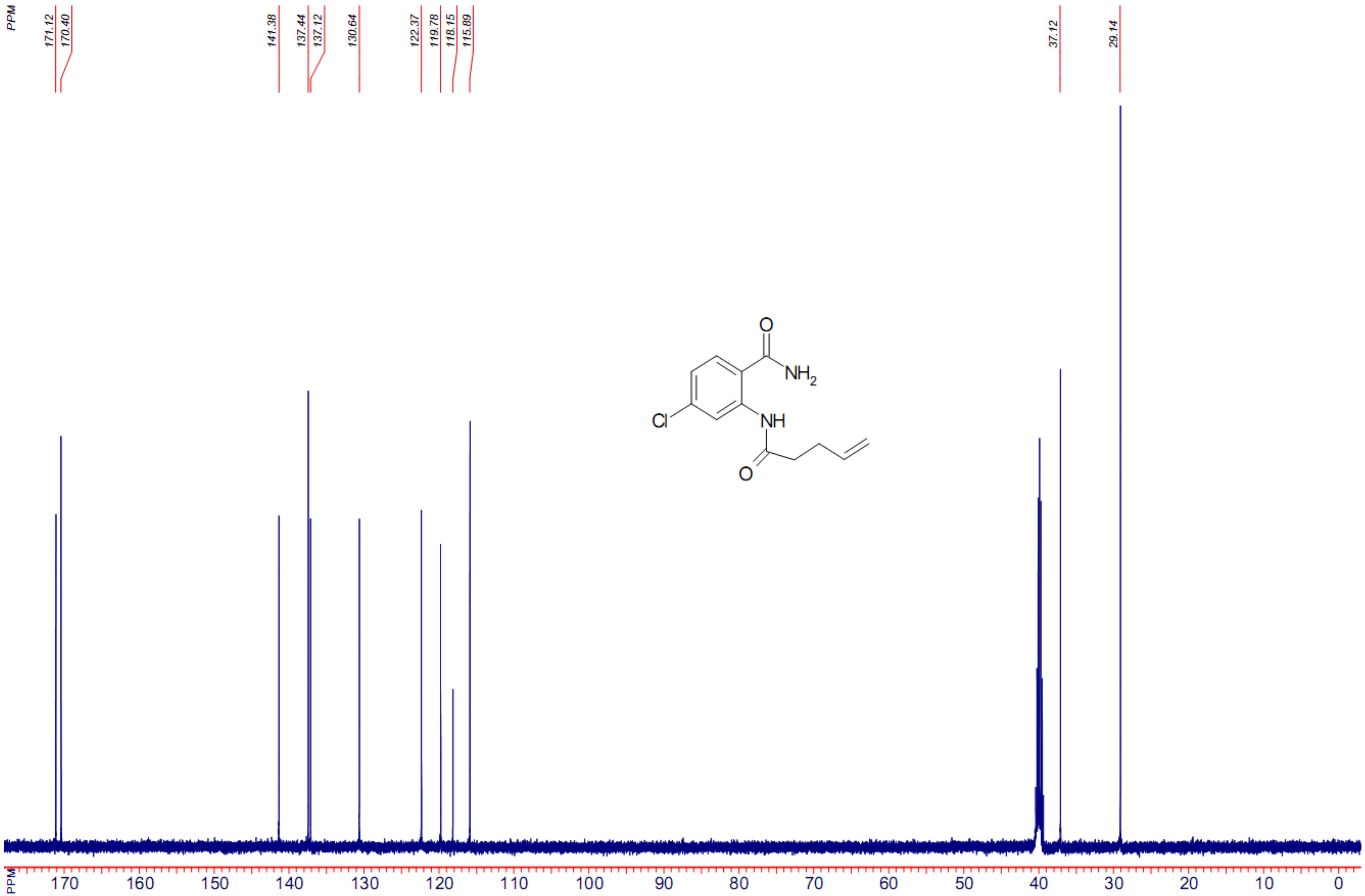

File name: phvm1481_C13.fid	Operator:	SF: 125.6927 MHz	NSC: 0	PW: 3.27 usec, RG: 60	SI: 65536
Date: 08-Apr-2021	Solvent: dmso	SW: 31250 Hz	TE: 298 K	AQ: 1.02 sec, RD: 0.00 sec	13C (1H-decoupled)

Figure S21. ^{1}H NMR spectrum (400 MHz, DMSO-d_6) of compound **10j**.

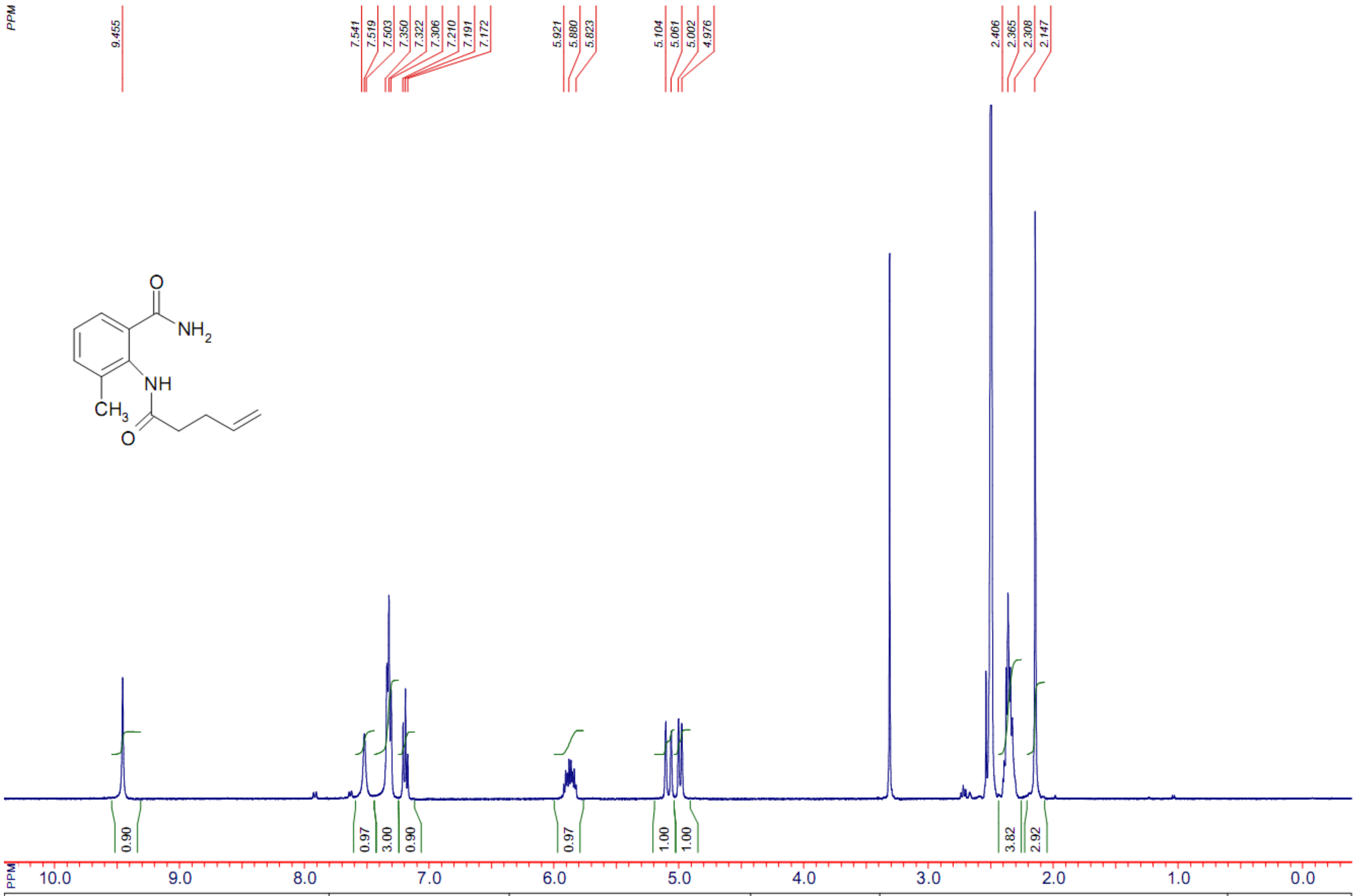

File name: PHVM110-5.fid	Operator:	SF: 399.9753 MHz	NSC: 0	PW: 10.90 usec, RG: 24	SI: 32768
Date: 01-Jul-2021	Solvent: dmso	SW: 8000 Hz	TE: 298 K	AQ: 2.00 sec, RD: 0.00 sec	□

Figure S22. **^{13}C NMR spectrum (125 MHz, DMSO-d_6) of compound 10j.**

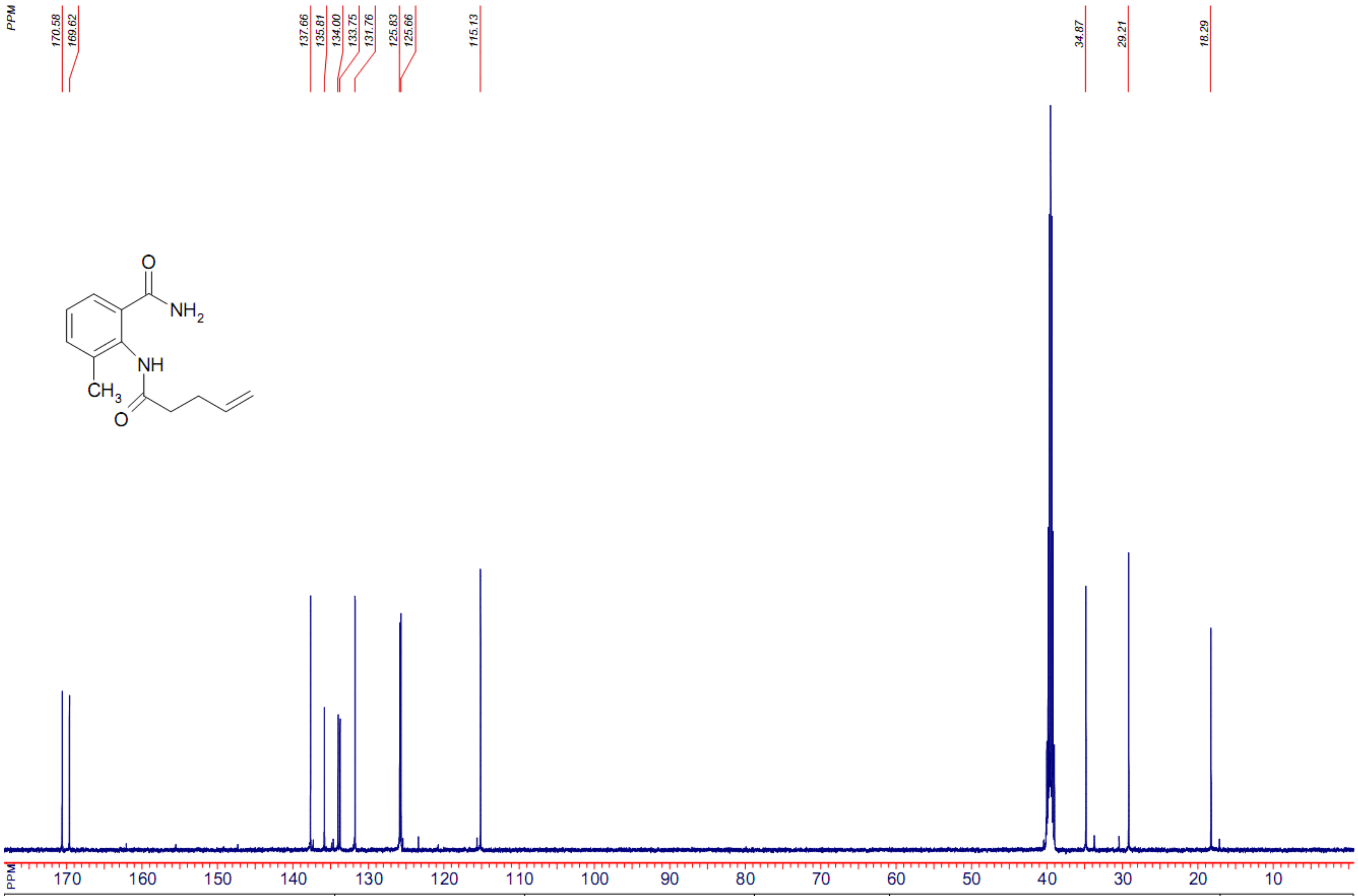

**File name: PHVM110-5_C13**	Operator: root	SF: 125.6681 MHz	NSC: 2290	PW: 0.00 usec, RG: 51200	SI: 131072
Date: 04-Jul-2021	Solvent: DMSO	SW: 32680 Hz	TE: 683 K	AQ: 1.00 sec, RD: 0.00 sec	

Figure S23. ^{1}H NMR spectrum (400 MHz, DMSO-d_6) of compound 10k.

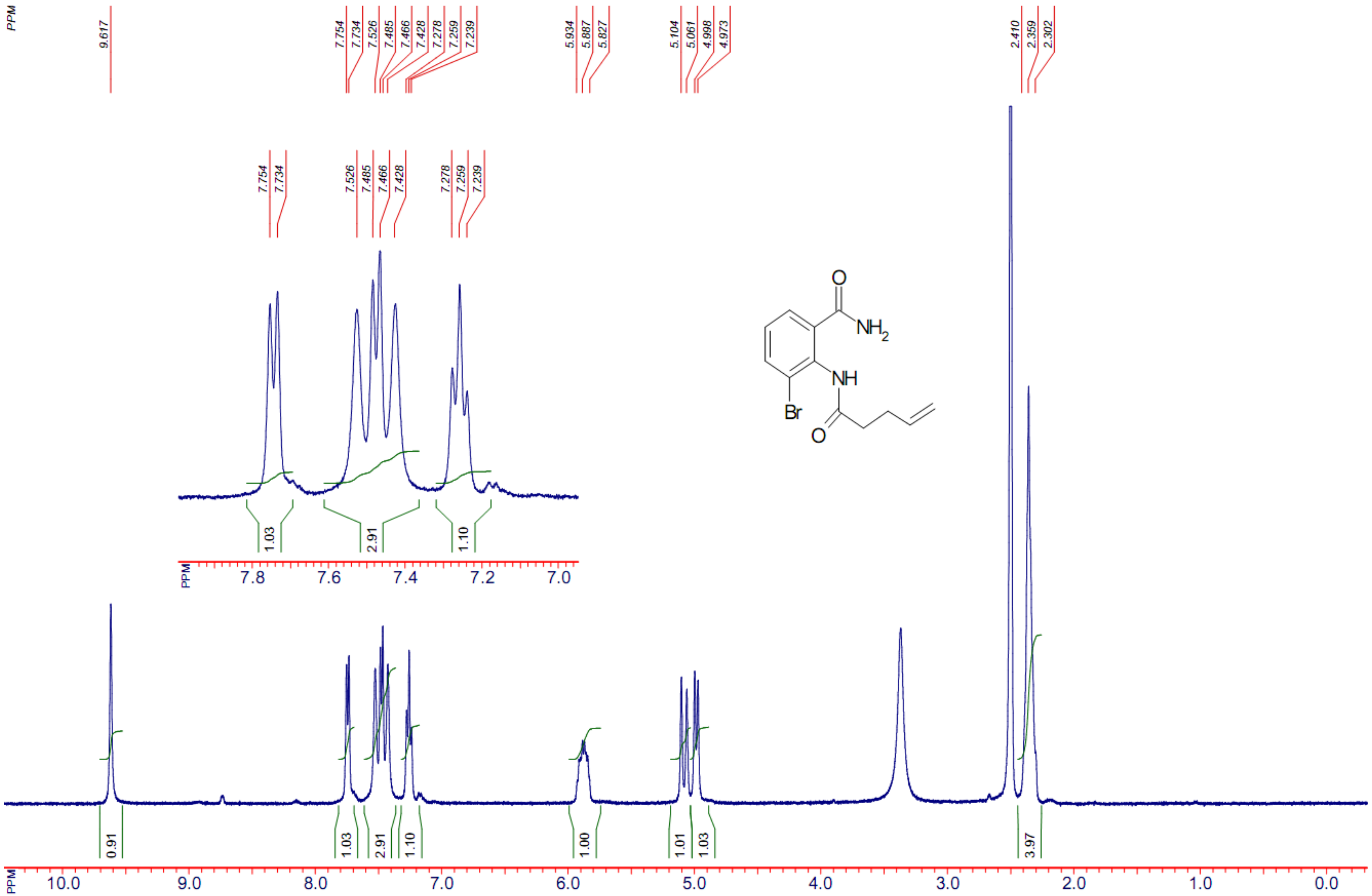

File name: PHvm53-7.fid	Operator:	SF: 399.9753 MHz	NSC: 0	PW: 10.90 usec, RG: 24	SI: 32768
Date: 18-Mar-2021	Solvent: dmso	SW: 8000 Hz	TE: 298 K	AQ: 2.00 sec, RD: 0.00 sec	□

Figure S24. **^{13}C NMR spectrum (100 MHz, DMSO-d_6) of compound 10k.**

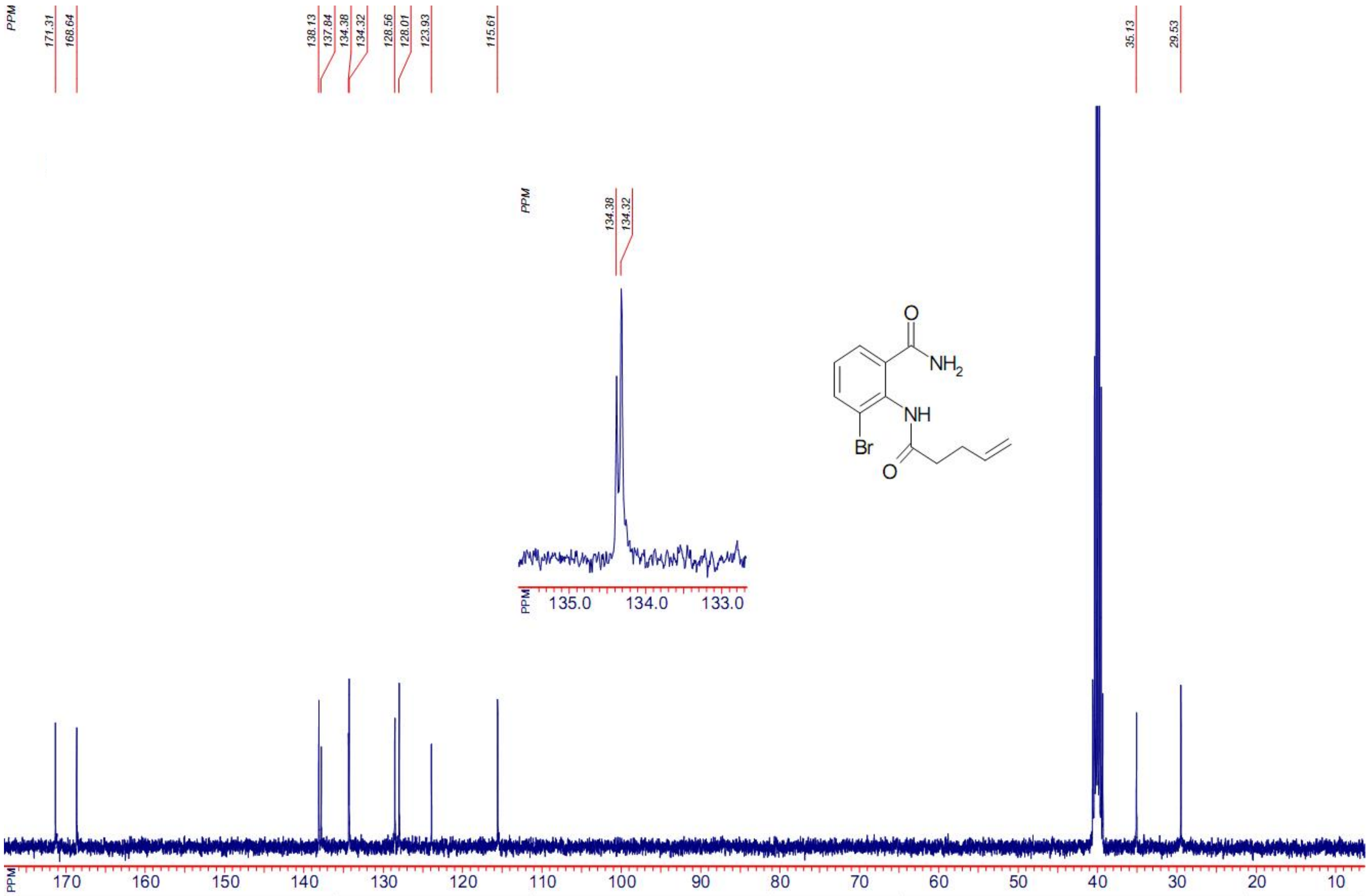

File name: PHVM53-7_C13	Operator: nmr□	SF: 100.6128 MHz	NSC: 402	PW: 0.00 usec, RG: 2050	SI: 32768
Date: 19-Mar-2021	Solvent: DMSO	SW: 26042 Hz	TE: 296 K	AQ: 0.98 sec, RD: 0.00 sec	□

Figure S25. ^{1}H **NMR spectrum (400 MHz, DMSO-*d*$_6$) of compound 10l.**

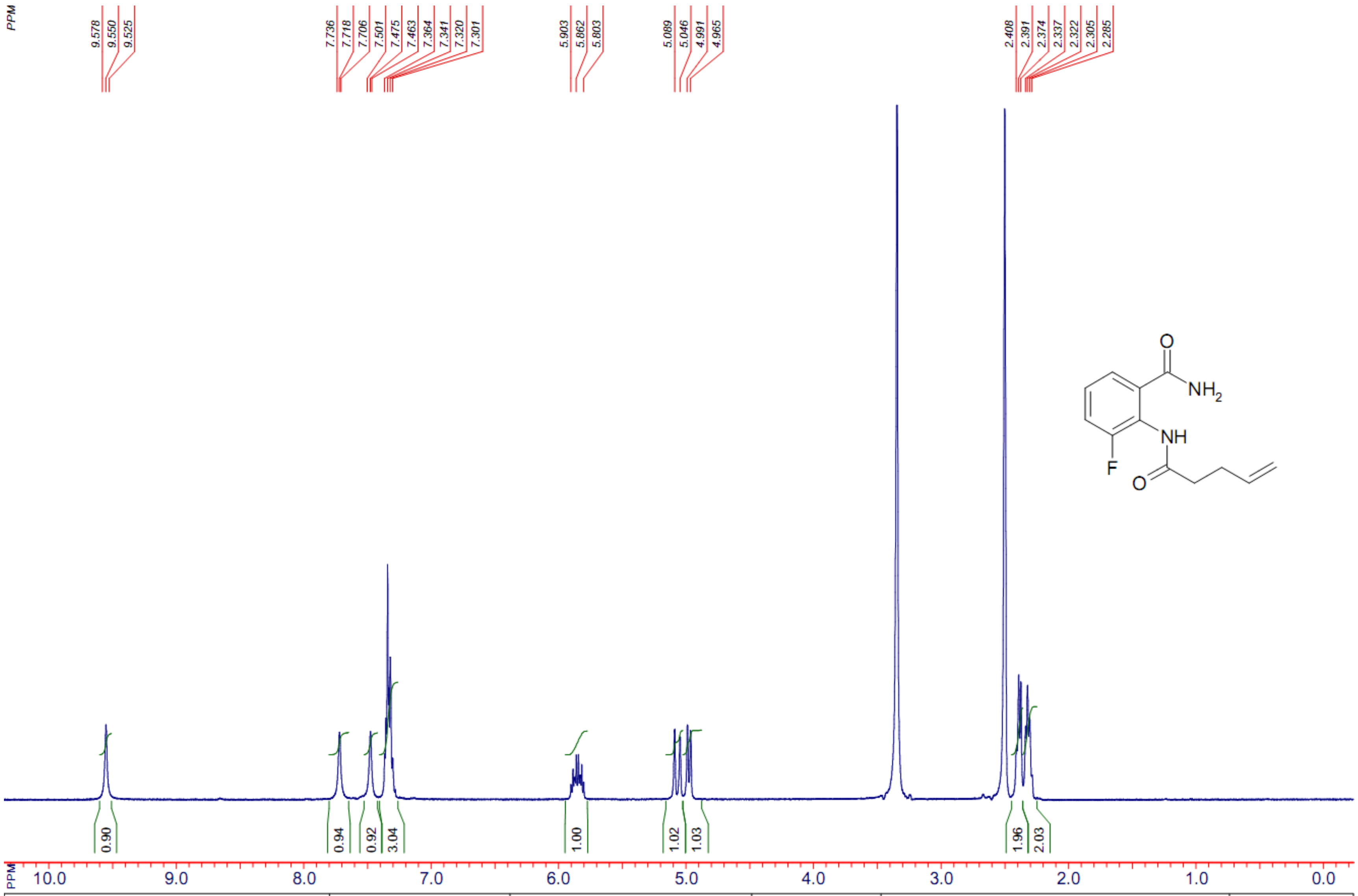

**File name: PHVM109-1.fid**	Operator:	SF: 399.9753 MHz	NSC: 0	PW: 10.90 usec, RG: 20	SI: 32768
Date: 22-Jun-2021	Solvent: dmso	SW: 8000 Hz	TE: 298 K	AQ: 2.00 sec, RD: 0.00 sec	□

Figure S26. [13]**C NMR spectrum (150 MHz, DMSO-*d*6) of compound 10l.**

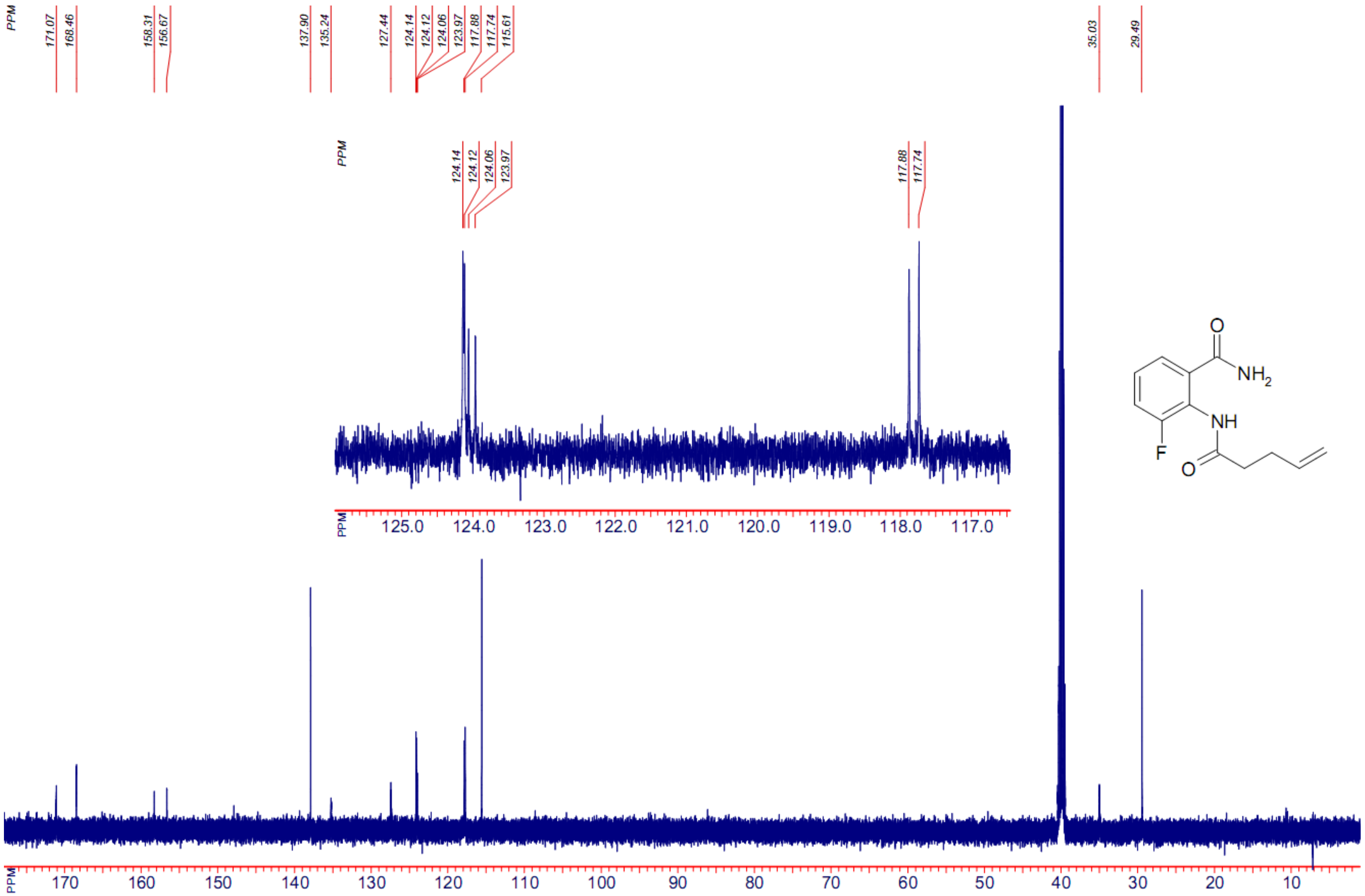

File name: phvm109-1_C13.fid	Operator:	SF: 150.8305 MHz	NSC: 0	PW: 3.09 usec, RG: 60	SI: 131072
Date: 24-Jun-2021	Solvent: dmso	SW: 37879 Hz	TE: 298 K	AQ: 1.73 sec, RD: 0.00 sec	□

Figure S27. ^{1}H NMR spectrum (400 MHz, DMSO-d_6) of compound 7a.

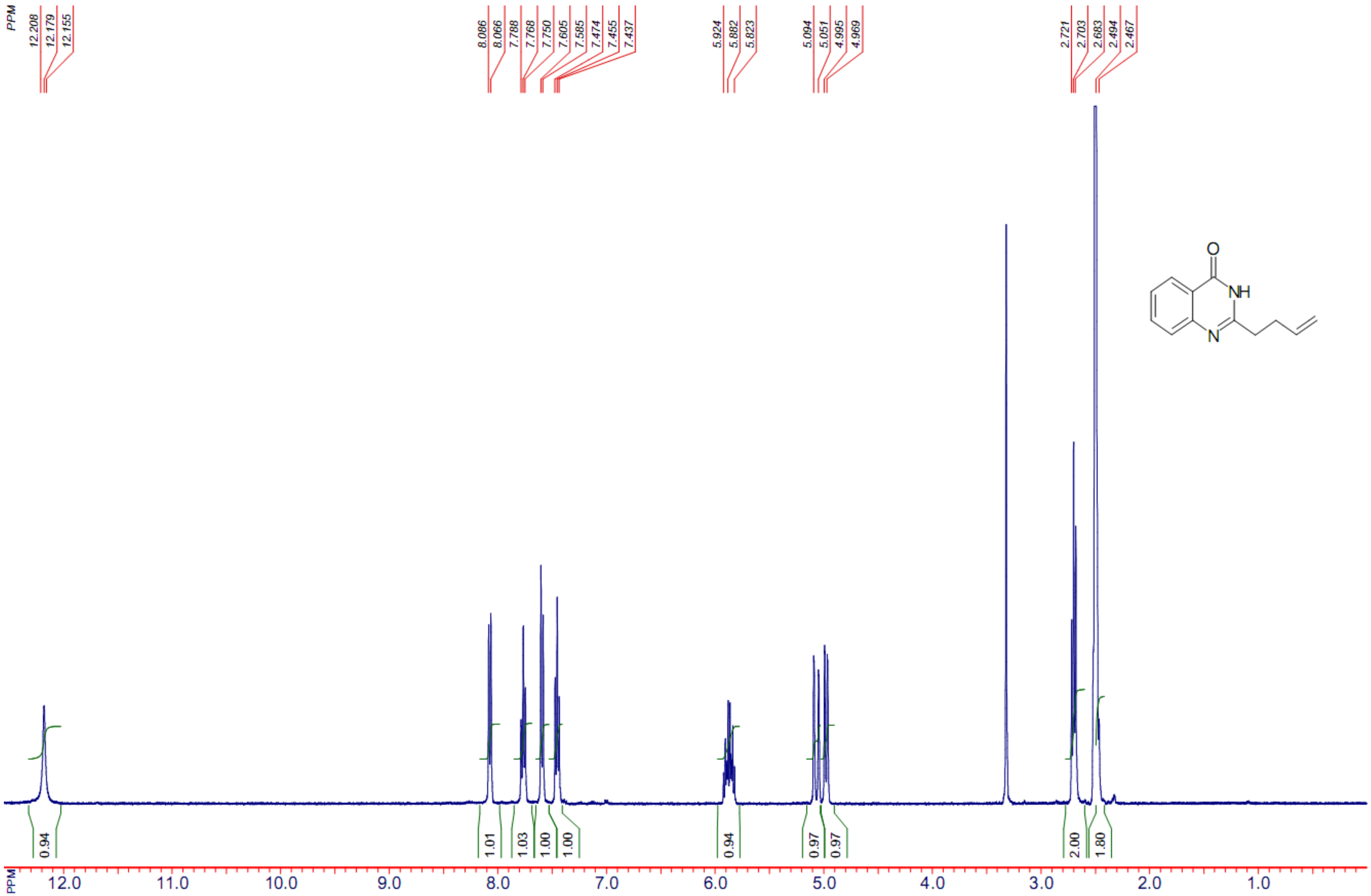

File name: PHvm1391.fid	Operator:	SF: 399.9755 MHz	NSC: 0	PW: 11.60 usec, RG: 30	SI: 32768
Date: 10-Sep-2020	Solvent: dmso	SW: 7599 Hz	TE: 294 K	AQ: 2.11 sec, RD: 0.00 sec	□

Figure S28. ^{13}**C NMR spectrum (125 MHz, DMSO-*d*$_6$) of compound 7a.**

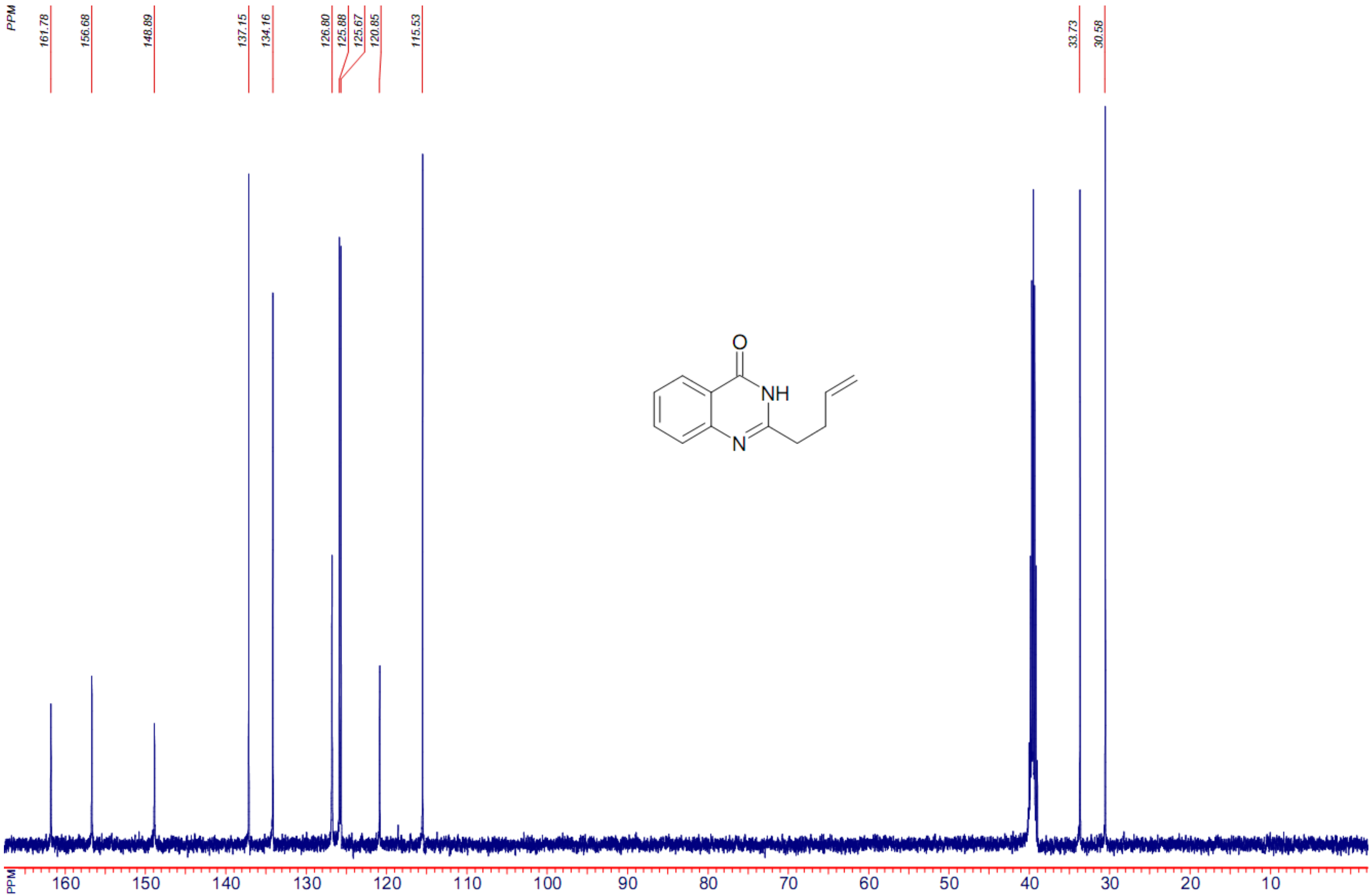

**File name: phvm1391_C13**	Operator: root	SF: 125.6681 MHz	NSC: 99	PW: 0.00 usec, RG: 51200	SI: 65536
Date: 11-Sep-2020	Solvent: DMSO	SW: 32680 Hz	TE: 683 K	AQ: 1.02 sec, RD: 0.00 sec	

Figure S29. ^{1}H NMR spectrum (400 MHz, DMSO-d_6) of compound **7b**.

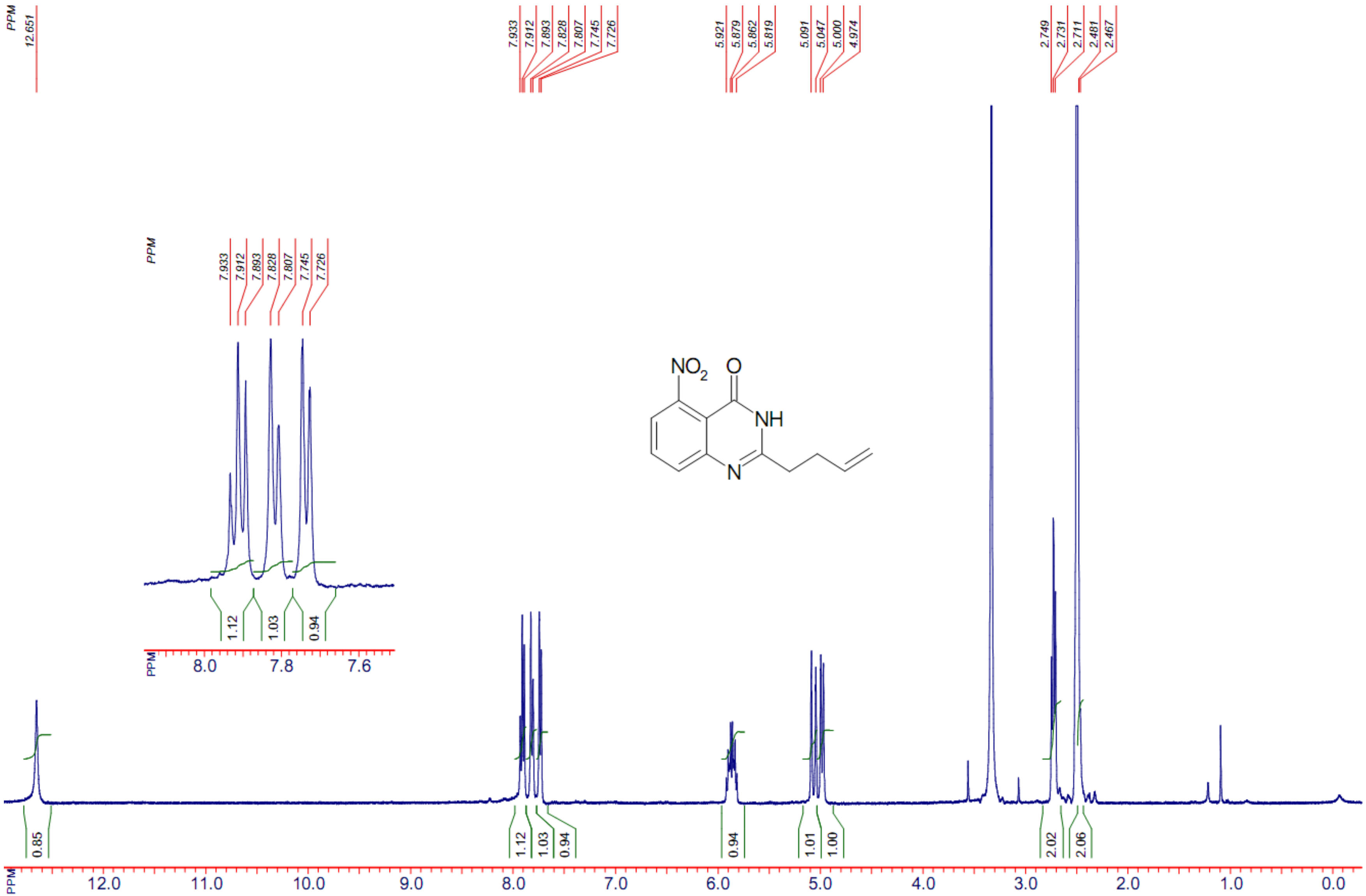

File name: PHvm86-4.fid	Operator:	SF: 399.9753 MHz	NSC: 0	PW: 10.90 usec, RG: 30	SI: 32768
Date: 13-May-2021	Solvent: dmso	SW: 8000 Hz	TE: 298 K	AQ: 2.00 sec, RD: 0.00 sec	□

Figure S30. ^{13}C NMR spectrum (150 MHz, DMSO-d_6) of compound 7b.

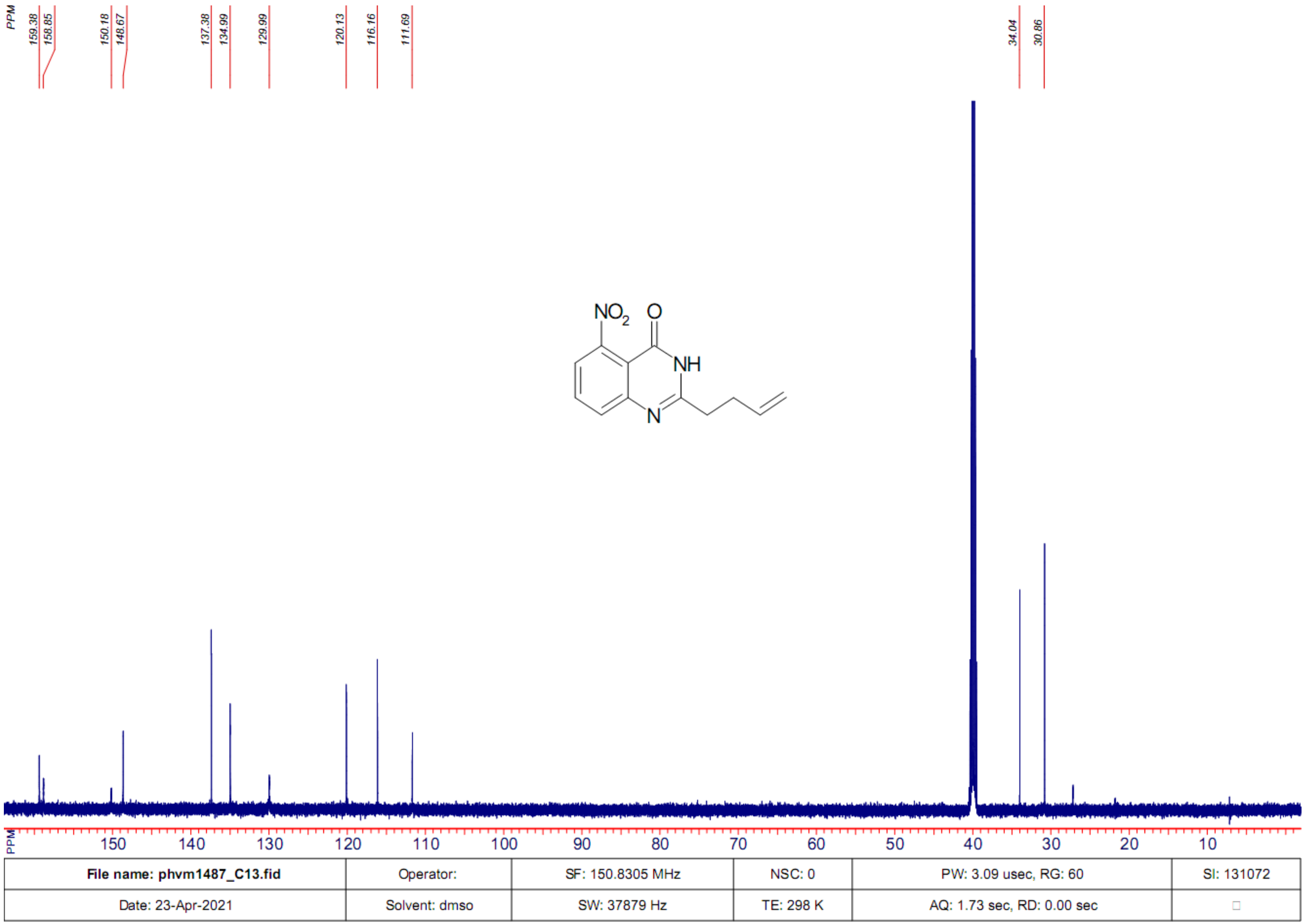

Figure S31. ^{1}H NMR spectrum (400 MHz, DMSO-d_6) of compound **7c**.

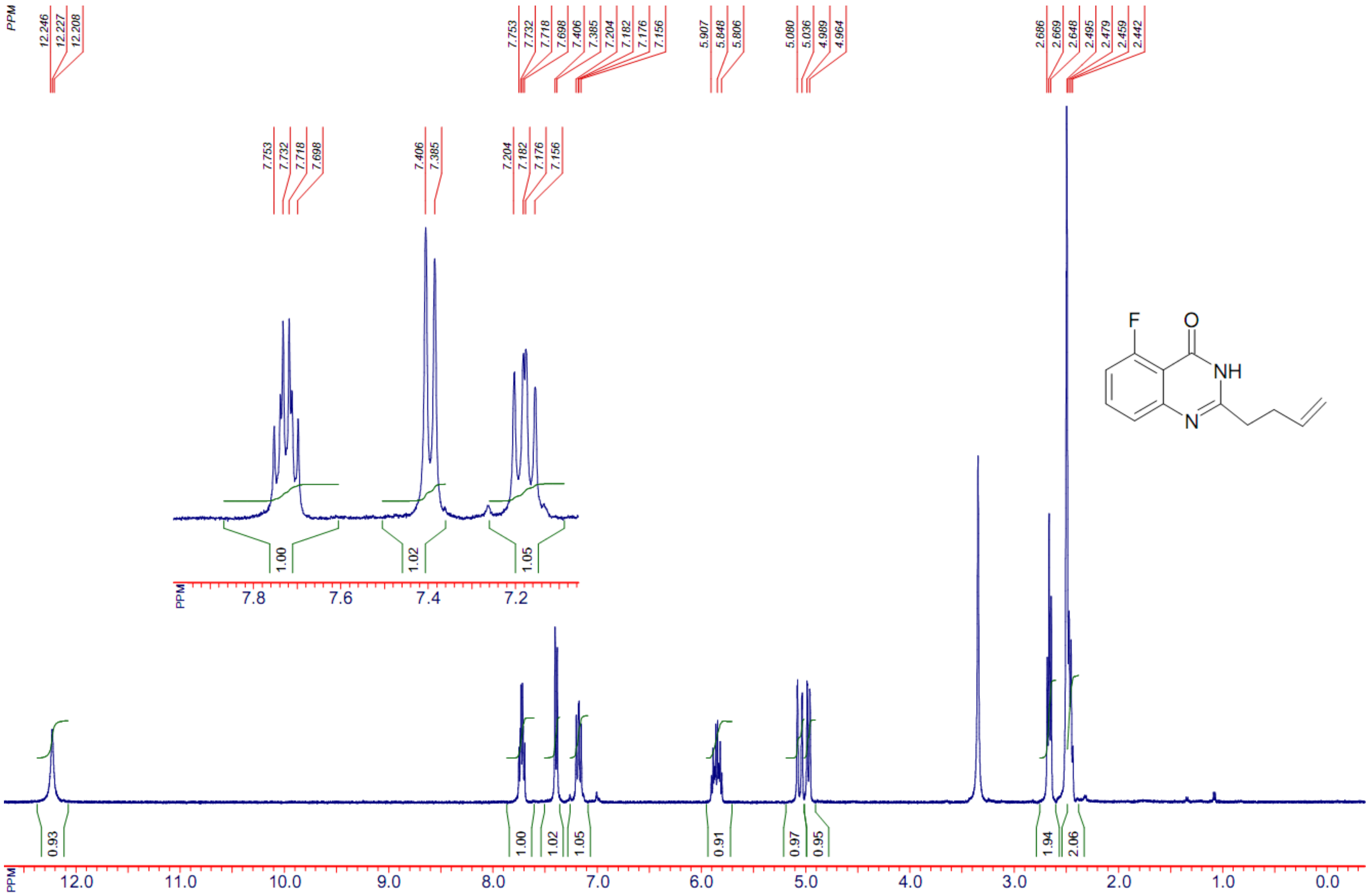

**File name: PHVM1478.fid**	Operator:	SF: 399.9753 MHz	NSC: 0	PW: 10.90 usec, RG: 20	SI: 32768
Date: 02-Apr-2021	Solvent: dmso	SW: 8000 Hz	TE: 298 K	AQ: 2.00 sec, RD: 0.00 sec	□

Figure S32. ^{13}C NMR spectrum (125 MHz, DMSO-d_6) of compound 7c.

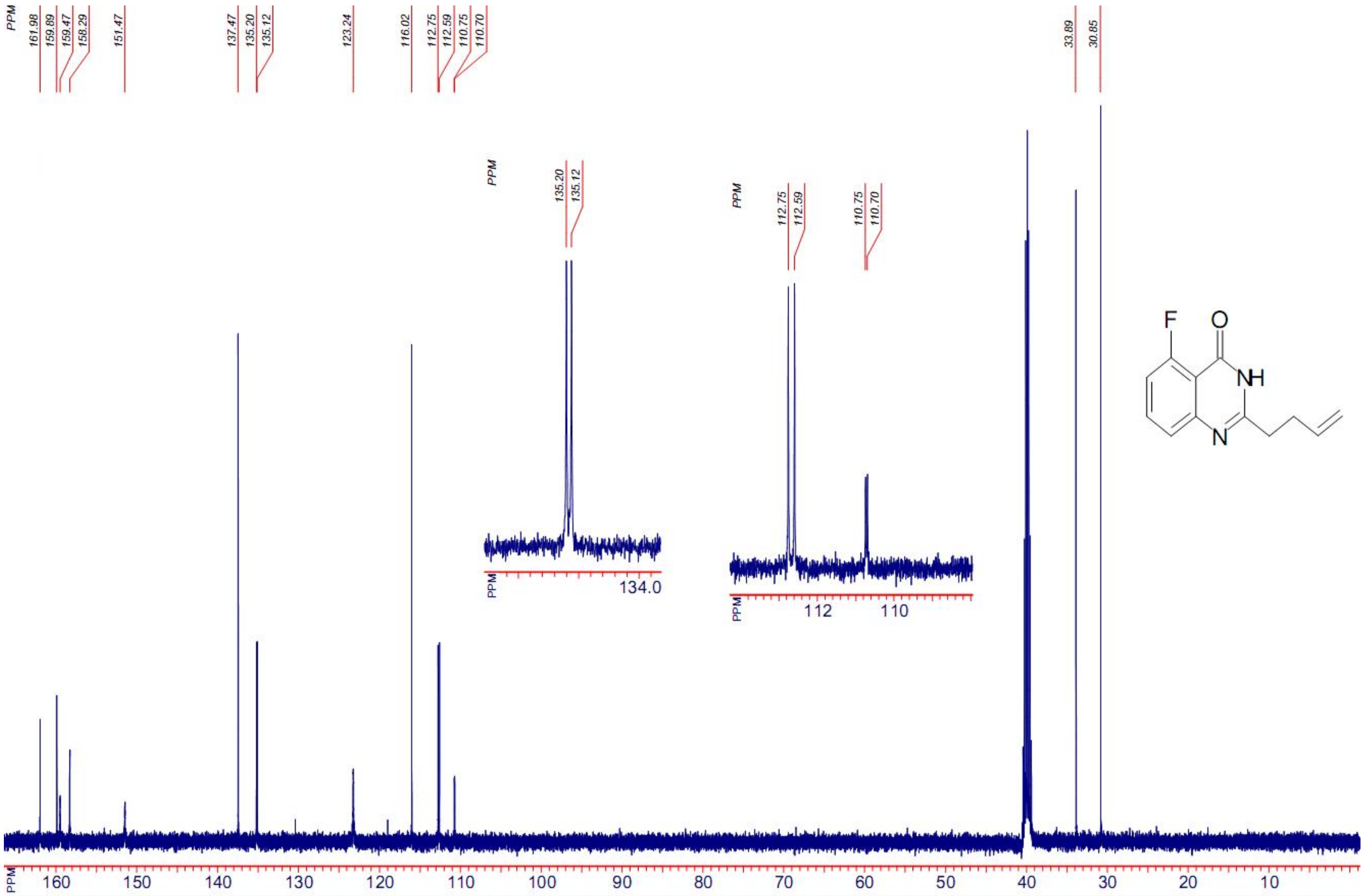

File name: PHVM1478_C13.fid	Operator:	SF: 125.6927 MHz	NSC: 0	PW: 3.27 usec, RG: 60	SI: 65536
Date: 06-Apr-2021	Solvent: dmso	SW: 31250 Hz	TE: 298 K	AQ: 1.02 sec, RD: 0.00 sec	13C (1H-decoupled)

Figure S33. **^{19}F NMR spectrum (188 MHz, DMSO-*d*6) of compound 7c.**

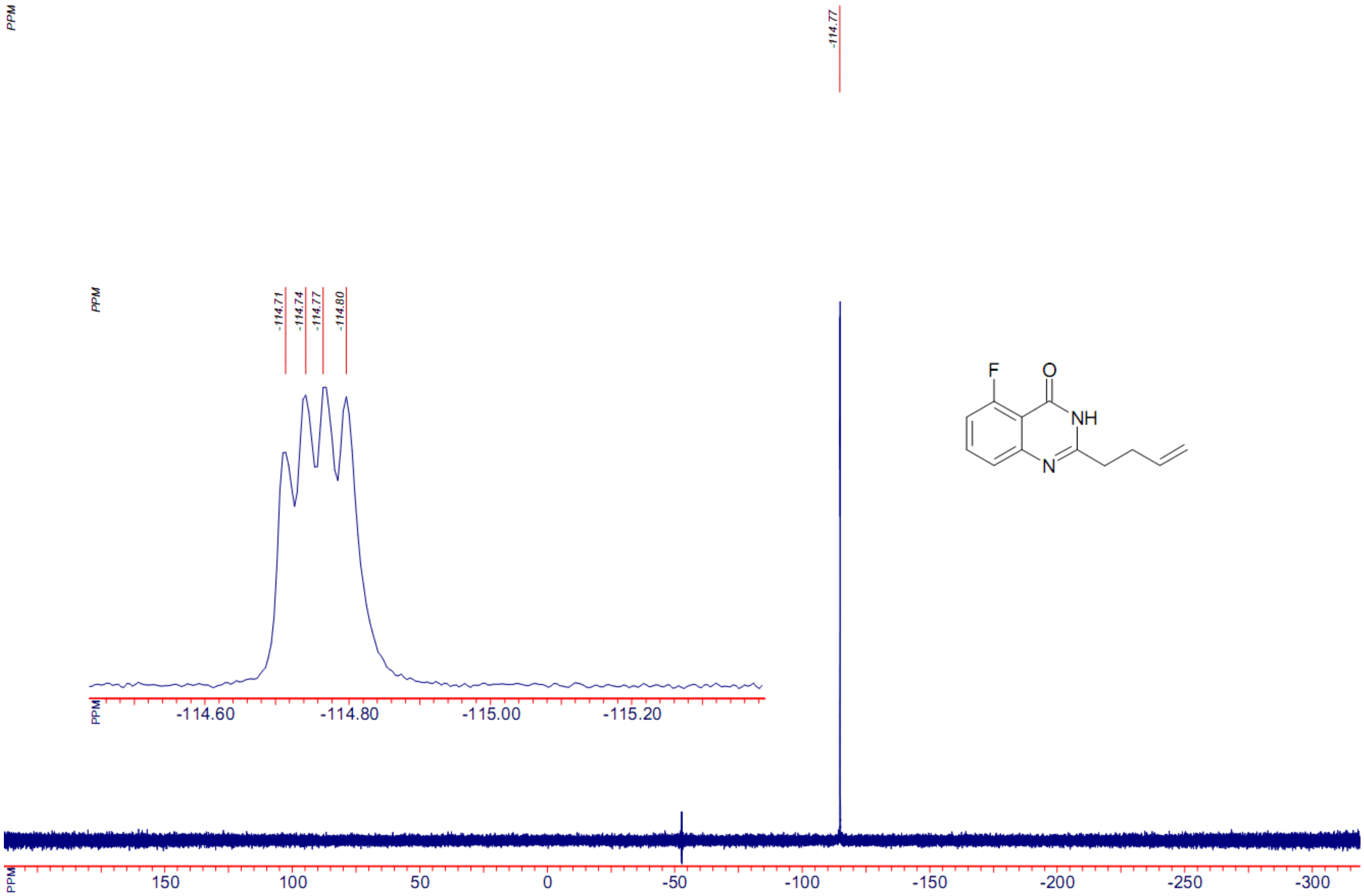

**File name: PHVM1478_v2_19F.fid**	Operator:	SF: 188.1362 MHz	NSC: 0	PW: 2.00 usec, RG: 0	SI: 131072
Date: 08-Jun-2021	Solvent: dmso	SW: 100000 Hz	TE: 298 K	AQ: 0.44 sec, RD: 0.00 sec	F19 PARAMETRES

Figure S34. ^{1}H NMR spectrum (400 MHz, DMSO-*d*6) of compound 7d.

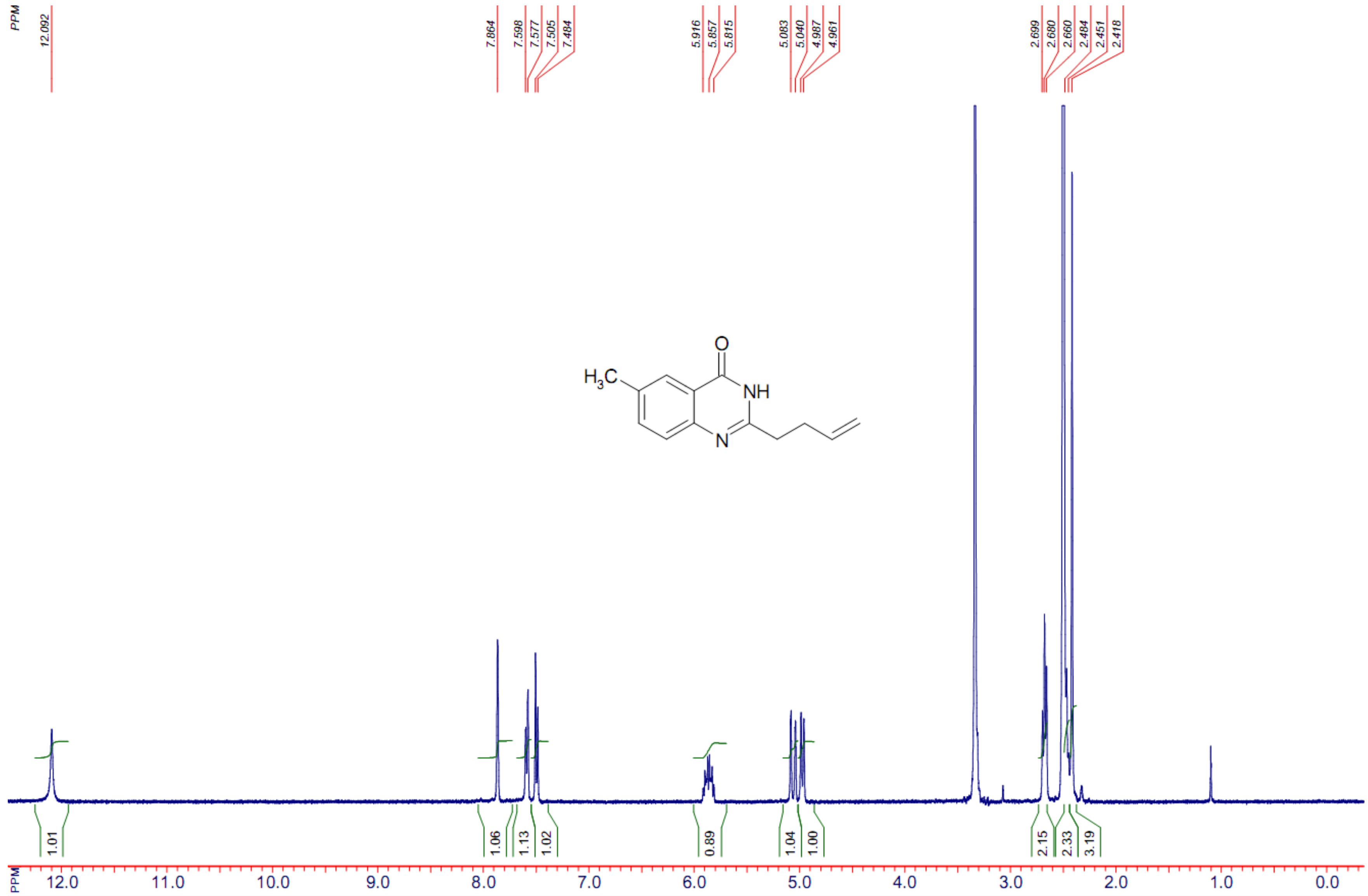

**File name: PHVM1522.fid**	Operator:	SF: 399.9753 MHz	NSC: 0	PW: 10.90 usec, RG: 24	SI: 32768
Date: 26-May-2021	Solvent: dmso	SW: 8000 Hz	TE: 298 K	AQ: 2.00 sec, RD: 0.00 sec	□

Figure S35. ^{13}C NMR spectrum (125 MHz, DMSO-d_6) of compound **7d**.

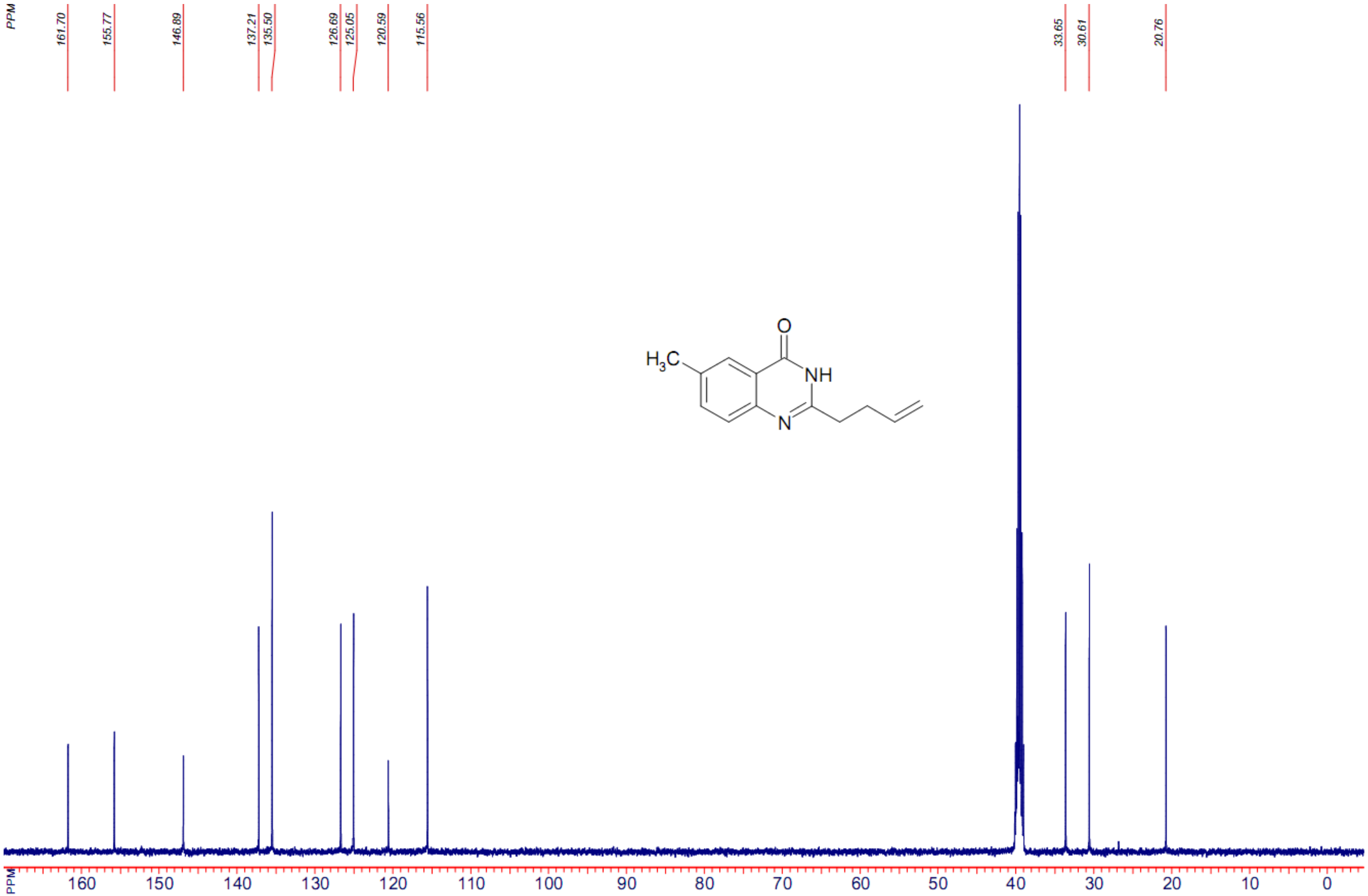

File name: PHvm1522_C13	Operator: root	SF: 125.6681 MHz	NSC: 916	PW: 0.00 usec, RG: 51200	SI: 131072
Date: 28-May-2021	Solvent: DMSO	SW: 32680 Hz	TE: 683 K	AQ: 0.78 sec, RD: 0.00 sec	

Figure S36. ^{1}H NMR spectrum (400 MHz, DMSO-*d*6) of compound 7e.

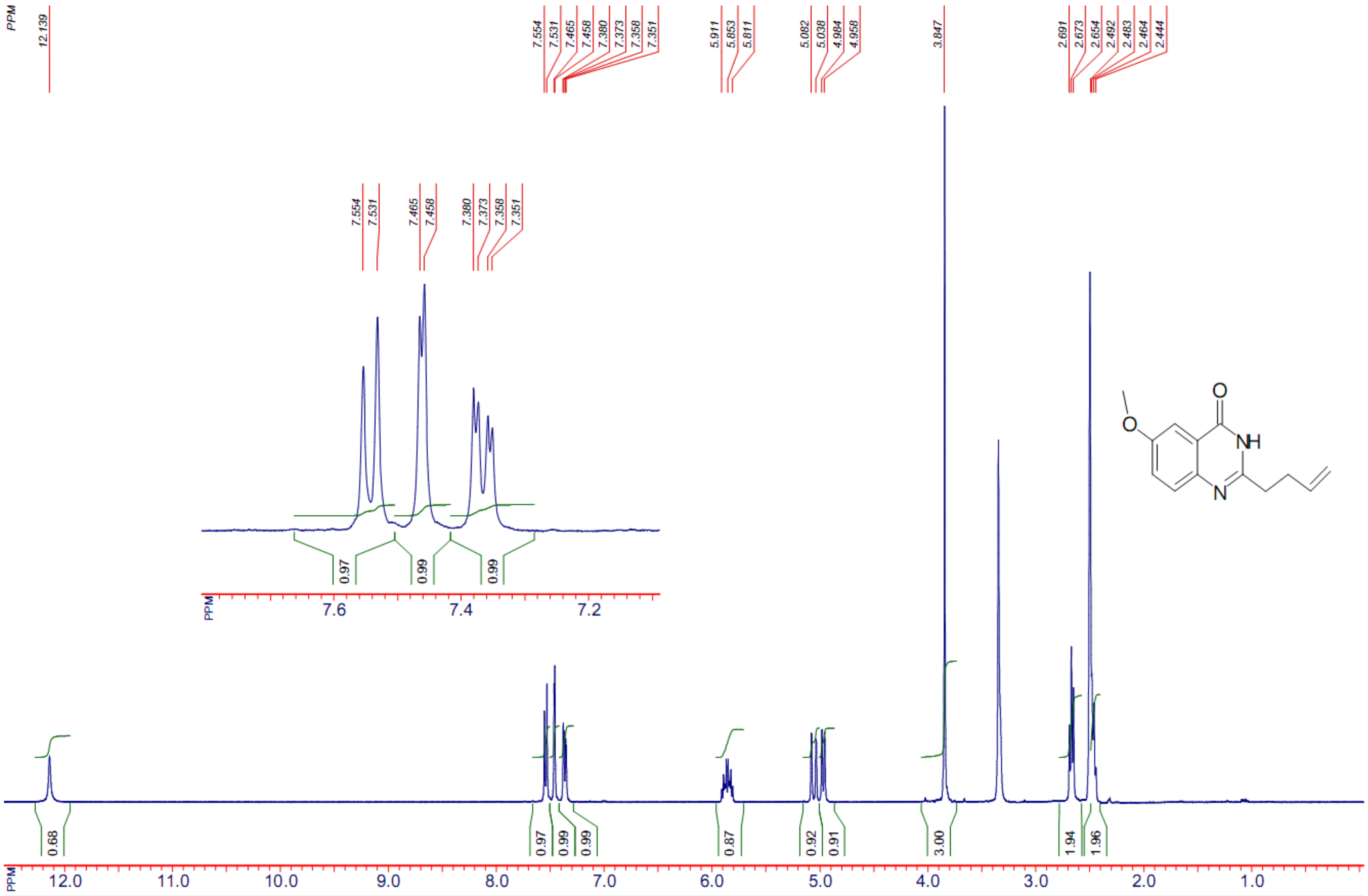

File name: PHVM1458.fid	Operator:	SF: 399.9755 MHz	NSC: 0	PW: 11.60 usec, RG: 20	SI: 32768
Date: 03-Mar-2021	Solvent: dmso	SW: 7599 Hz	TE: 294 K	AQ: 2.11 sec, RD: 0.00 sec	□

Figure S37. ^{13}C NMR spectrum (150 MHz, DMSO-d_6) of compound 7e

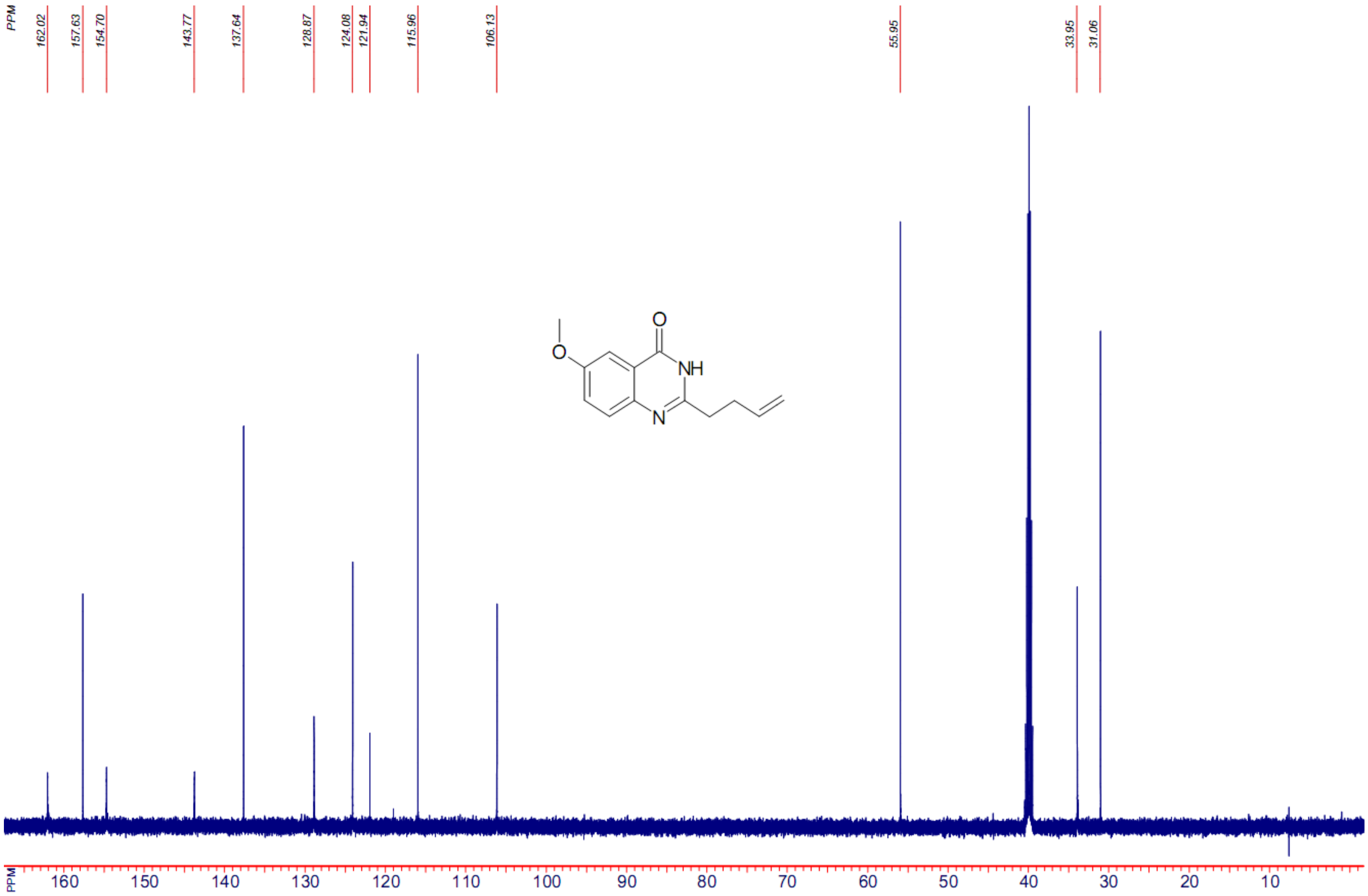

**File name: phvm1458_C13.fid**	Operator:	SF: 150.8303 MHz	NSC: 0	PW: 3.09 usec, RG: 60	SI: 131072
Date: 05-Mar-2021	Solvent: dmso	SW: 37879 Hz	TE: 298 K	AQ: 1.73 sec, RD: 0.00 sec	□

Figure S38. ^{1}H NMR spectrum (400 MHz, DMSO-*d*6) of compound 7f

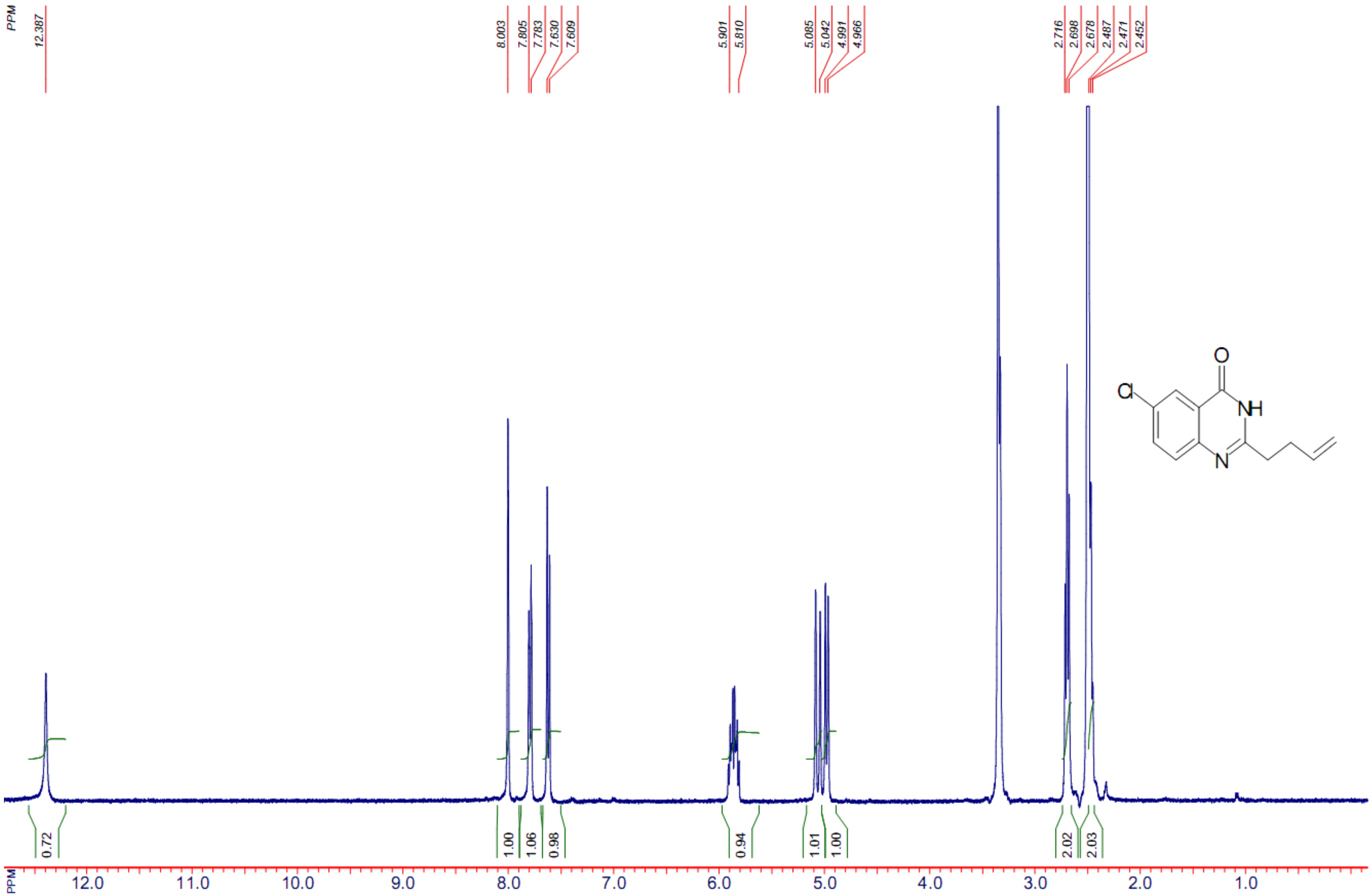

**File name: PHvm1454.fid**	Operator:	SF: 399.9755 MHz	NSC: 0	PW: 11.60 usec, RG: 24	SI: 32768
Date: 03-Mar-2021	Solvent: dmso	SW: 7599 Hz	TE: 294 K	AQ: 2.11 sec, RD: 0.00 sec	□

Figure S39. **^{13}C NMR spectrum (125 MHz, DMSO-*d*$_6$) of compound 7f**

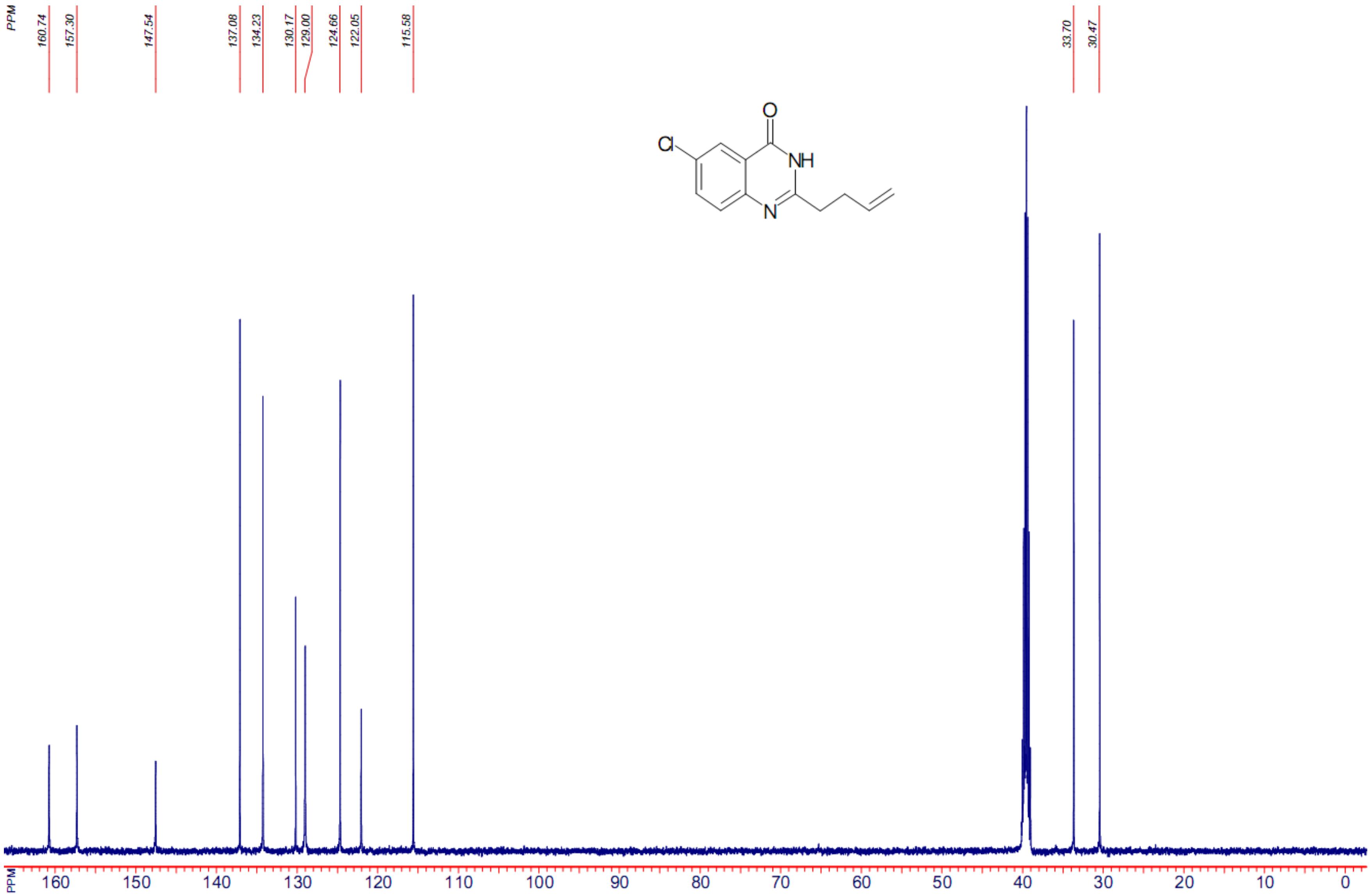

**File name: phvm8_C13**	Operator: root□	SF: 125.6681 MHz	NSC: 454	PW: 0.00 usec, RG: 51200	SI: 131072
Date: 20-Nov-2020	Solvent: DMSO	SW: 32680 Hz	TE: 683 K	AQ: 1.02 sec, RD: 0.00 sec	□

Figure S40. ^{1}H NMR spectrum (400 MHz, DMSO-*d*6) of compound 7g

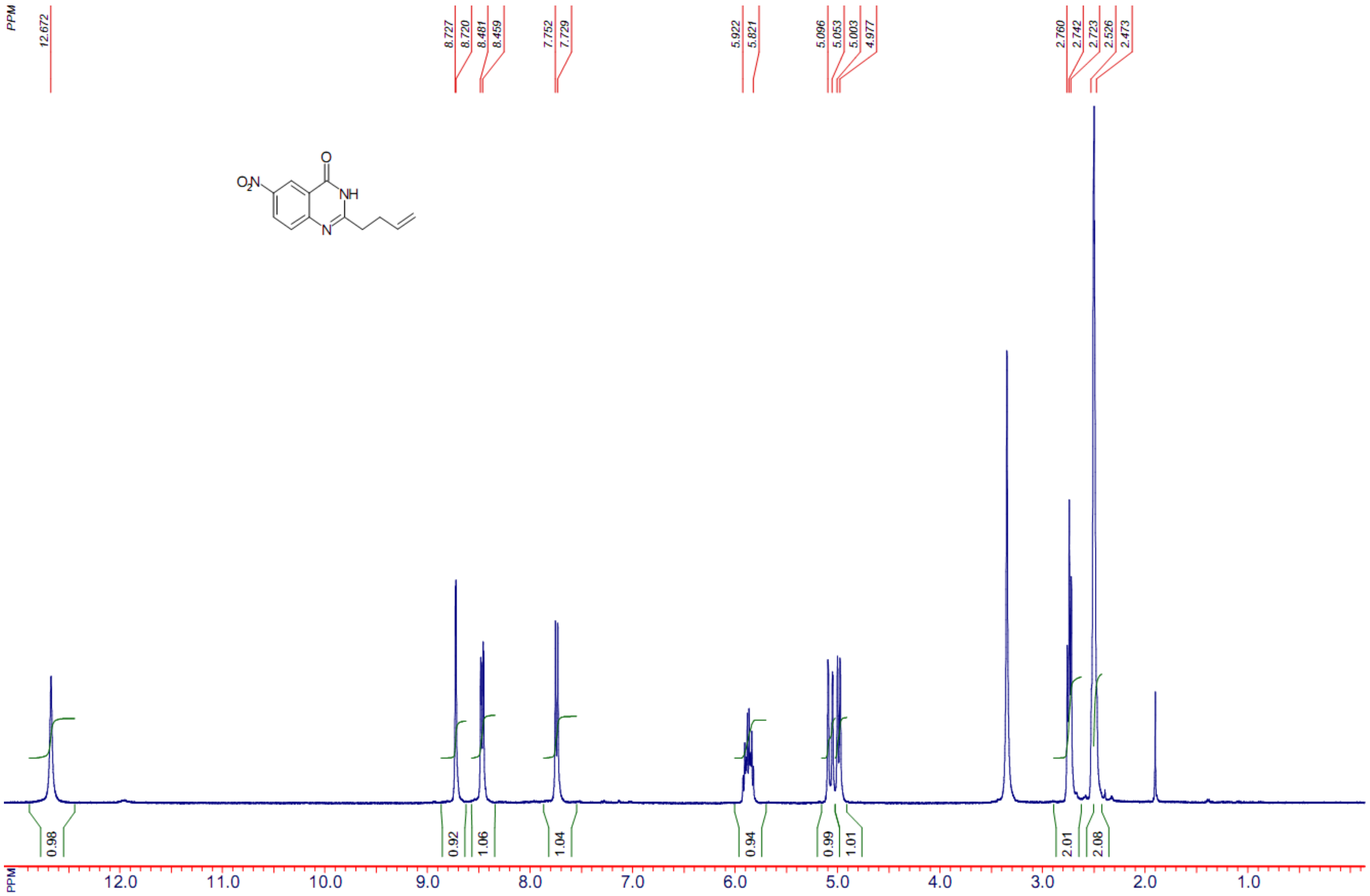

File name: PHvm1426.fid	Operator:	SF: 399.9755 MHz	NSC: 0	PW: 11.60 usec, RG: 30	SI: 32768
Date: 17-Dec-2020	Solvent: dmso	SW: 7599 Hz	TE: 294 K	AQ: 2.11 sec, RD: 0.00 sec	□

Figure S41. ^{13}C NMR spectrum (125 MHz, DMSO-d_6) of compound 7g**

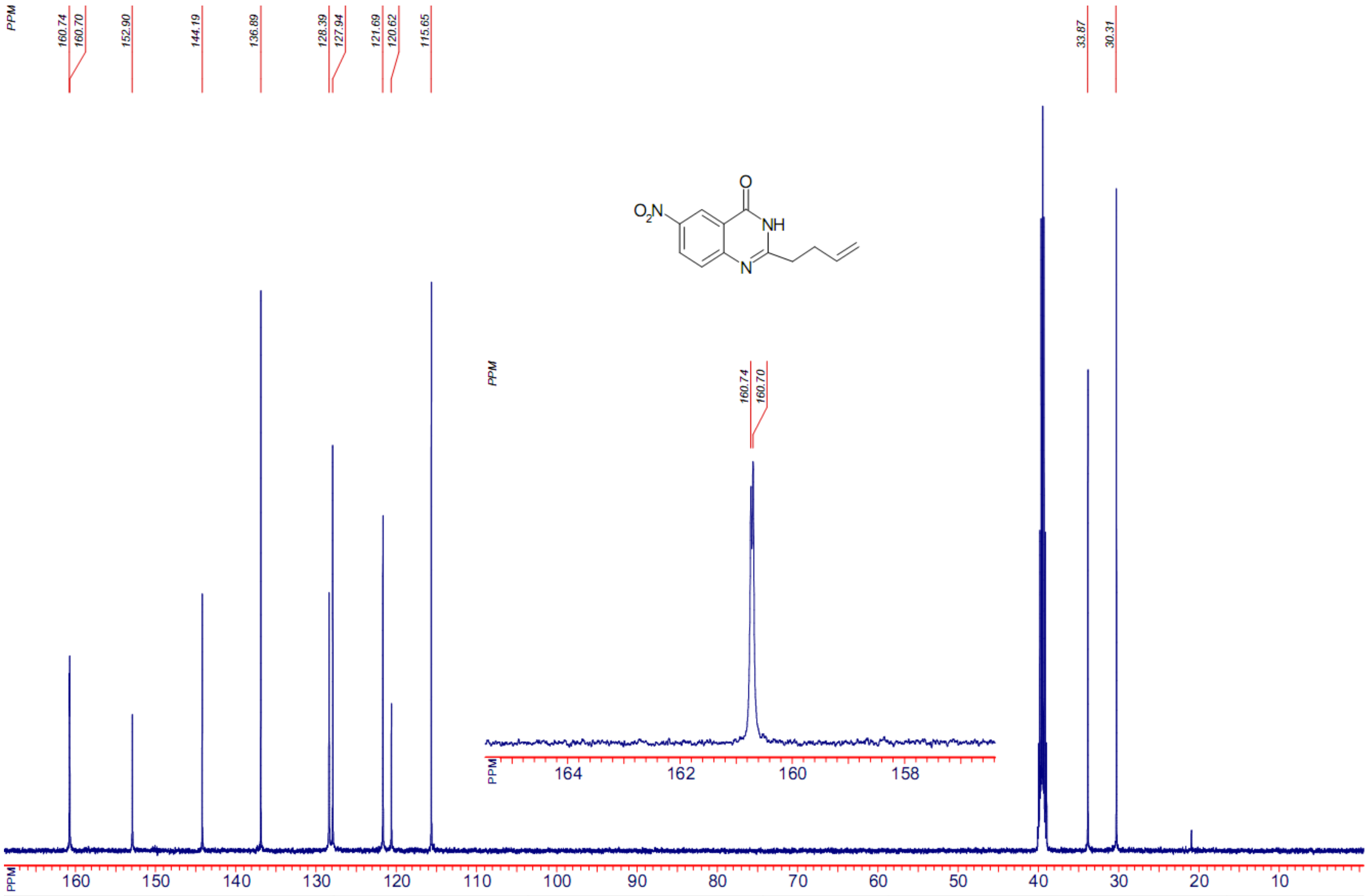

**File name: PHVM1426_C13**	Operator: root□	SF: 125.6681 MHz	NSC: 748	PW: 0.00 usec, RG: 51200	SI: 131072
Date: 18-Dec-2020	Solvent: DMSO	SW: 32680 Hz	TE: 683 K	AQ: 1.02 sec, RD: 0.00 sec	□

Figure S42. **^{1}H NMR spectrum (400 MHz, DMSO-d_6) of compound 7h**

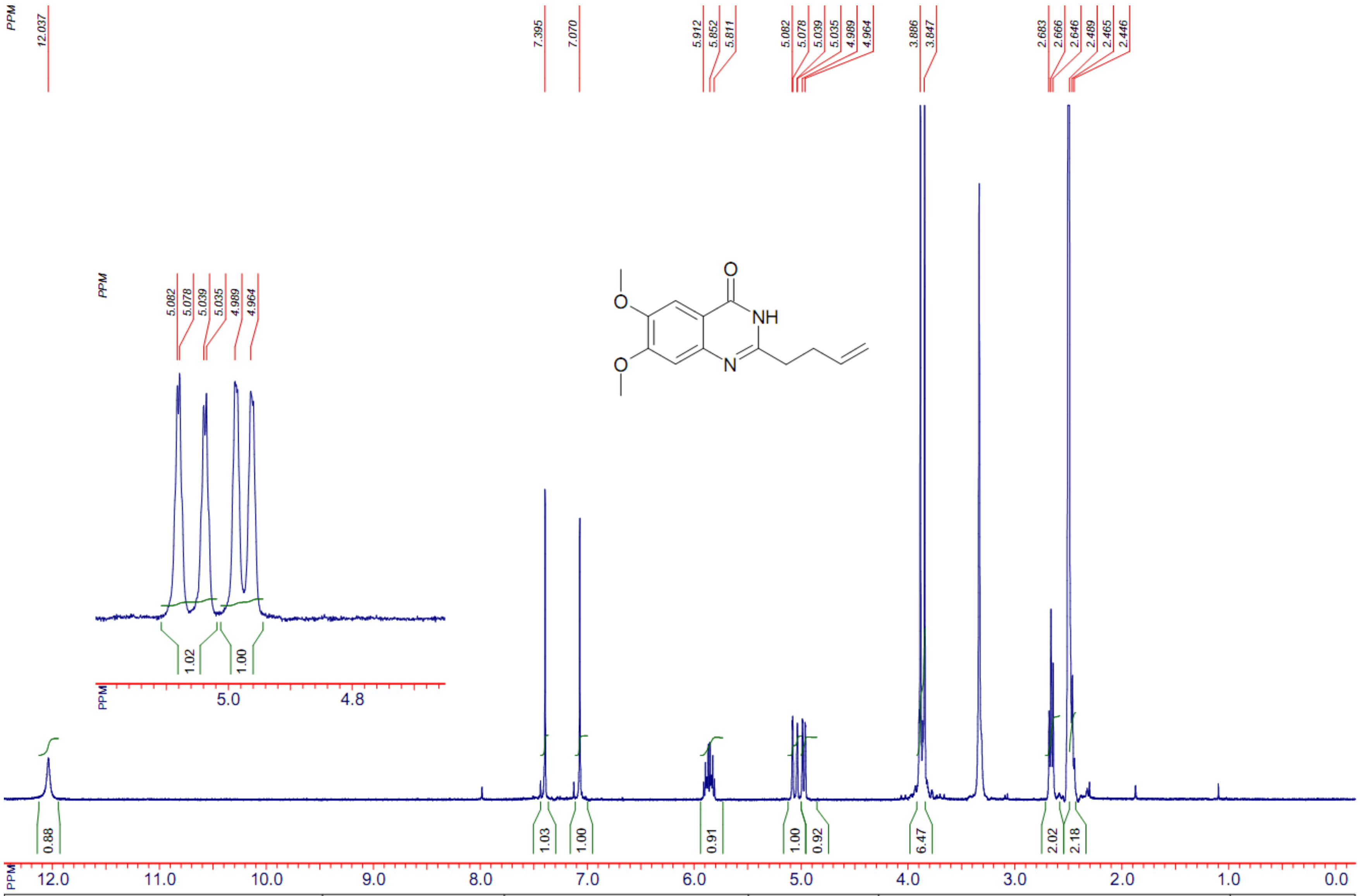

**File name: PHVM1451.fid**	Operator:	SF: 399.9753 MHz	NSC: 0	PW: 10.90 usec, RG: 24	SI: 32768
Date: 16-Apr-2021	Solvent: dmso	SW: 8000 Hz	TE: 298 K	AQ: 2.00 sec, RD: 0.00 sec	□

Figure S43. ^{13}C NMR spectrum (125 MHz, DMSO-d_6) of compound 7h

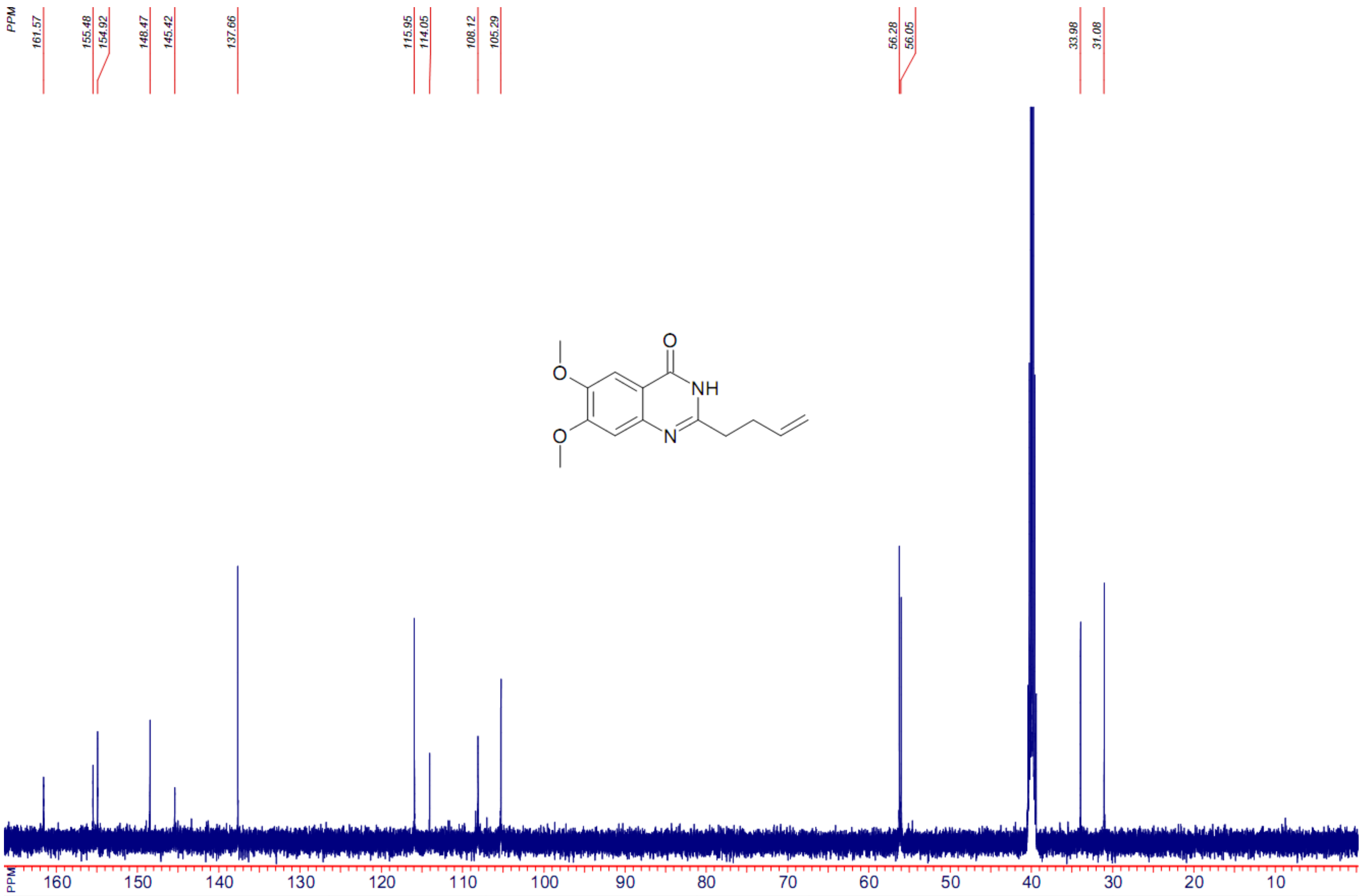

**File name: phvm1451_C13.fid**	Operator:	SF: 125.6927 MHz	NSC: 0	PW: 3.27 usec, RG: 60	SI: 65536
Date: 20-Apr-2021	Solvent: dmso	SW: 31250 Hz	TE: 298 K	AQ: 1.02 sec, RD: 0.00 sec	13C (1H-decoupled)

Figure S44. [1]**H NMR spectrum (400 MHz, DMSO-*d*6) of compound 7i**

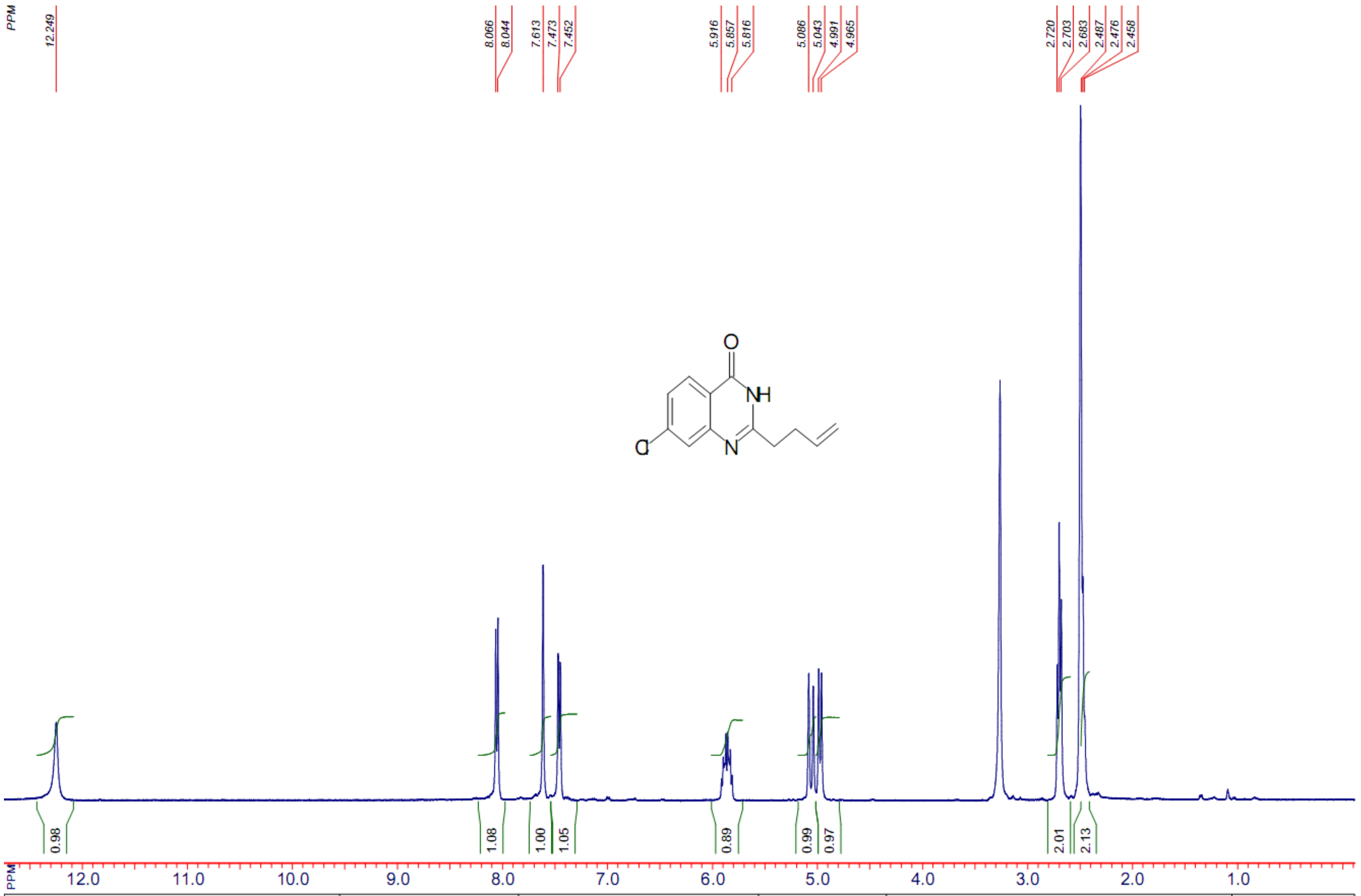

File name: PHvm1484-1.fid	Operator:	SF: 399.9753 MHz	NSC: 0	PW: 10.90 usec, RG: 24	SI: 32768
Date: 09-Apr-2021	Solvent: dmso	SW: 8000 Hz	TE: 298 K	AQ: 2.00 sec, RD: 0.00 sec	□

Figure S45. ^{13}C NMR spectrum (150 MHz, DMSO-d_6) of compound 7i

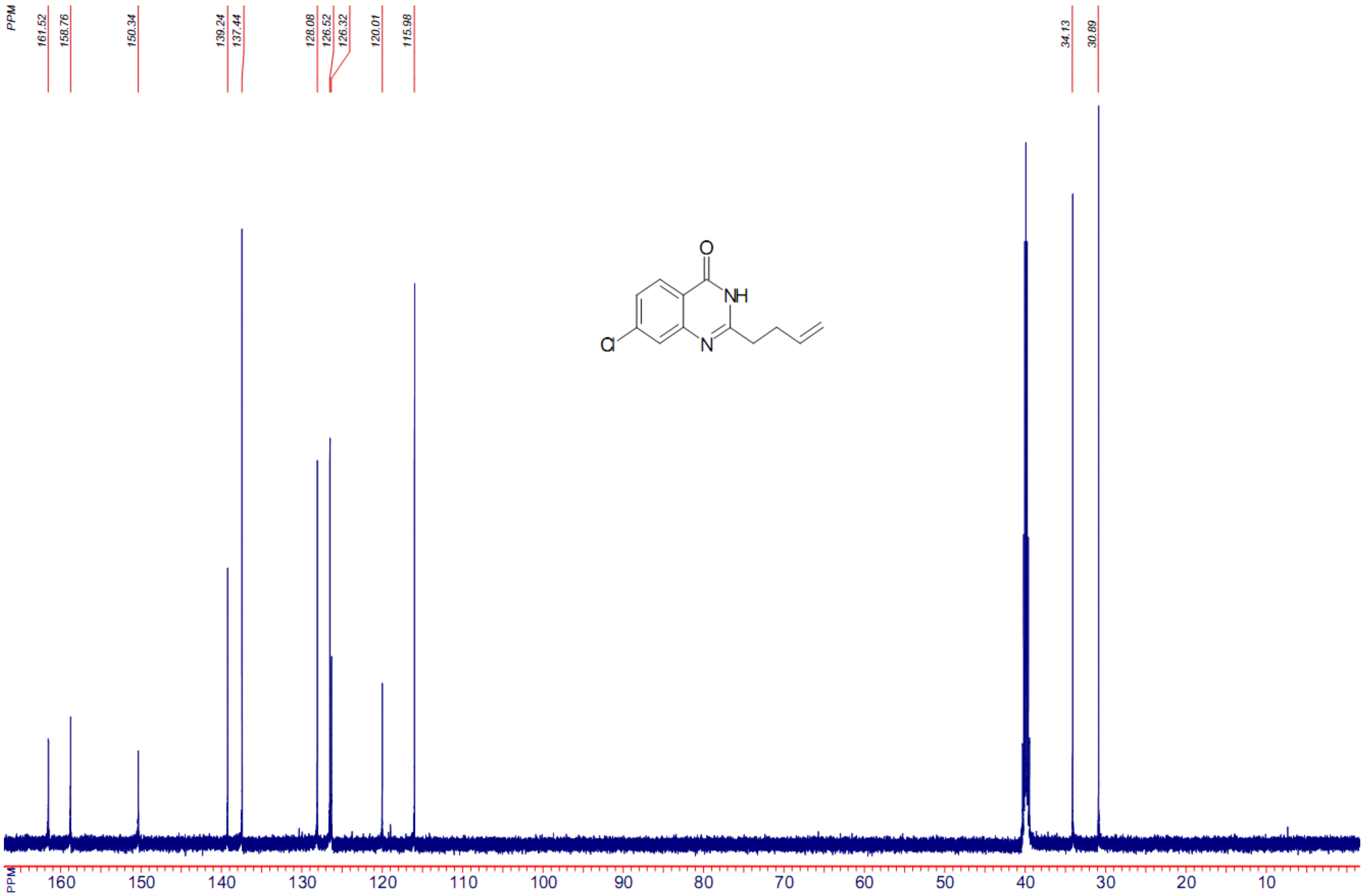

**File name: phvm1484_C13.fid**	Operator:	SF: 150.8305 MHz	NSC: 0	PW: 3.09 usec, RG: 60	SI: 131072
Date: 09-Apr-2021	Solvent: dmso	SW: 37879 Hz	TE: 298 K	AQ: 1.73 sec, RD: 0.00 sec	□

Figure S46. ^{1}H NMR spectrum (400 MHz, DMSO-d_6) of compound 7j

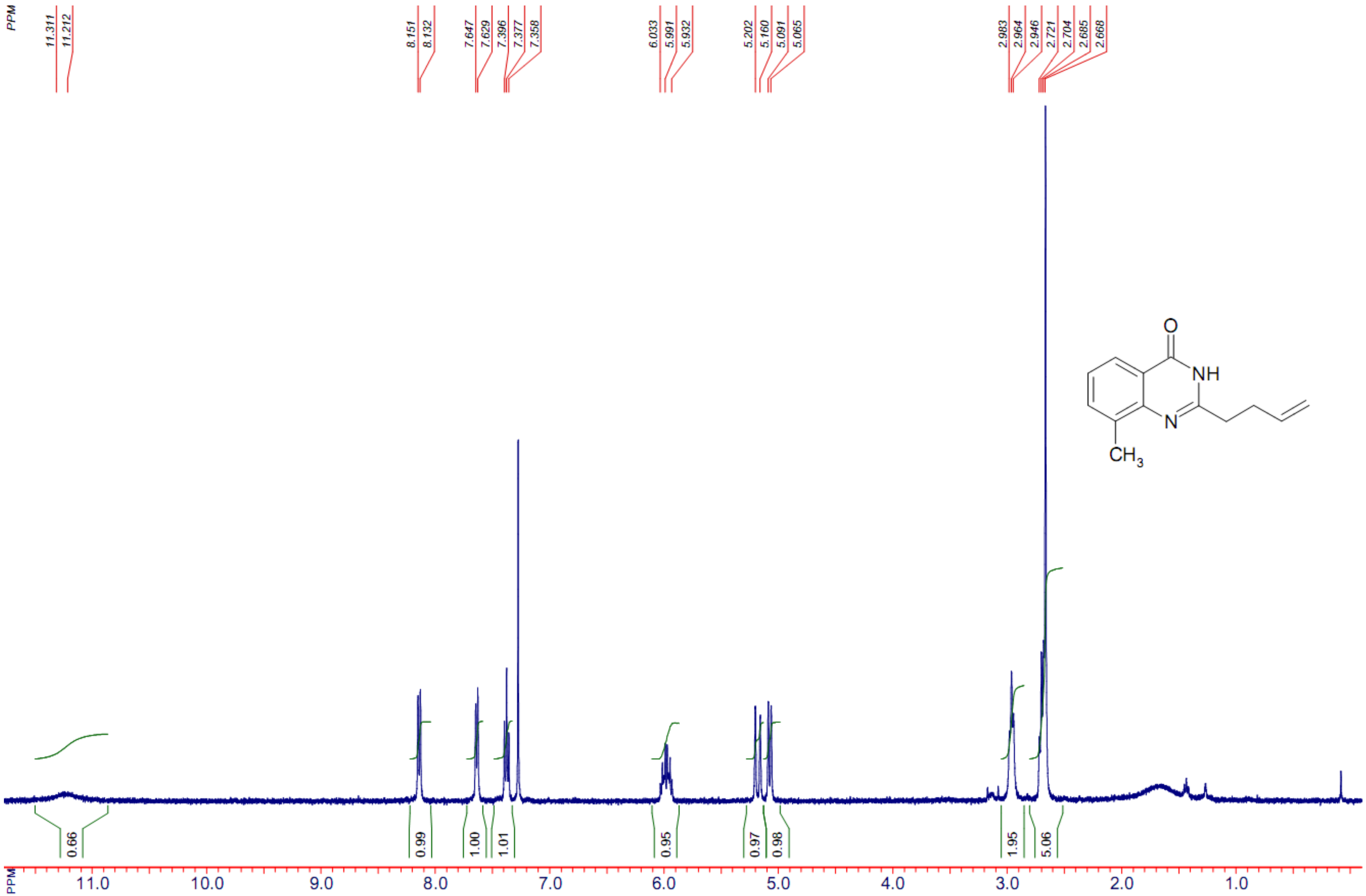

**File name: PHvm94-12.fid**	Operator:	SF: 399.9734 MHz	NSC: 0	PW: 10.90 usec, RG: 24	SI: 32768
Date: 21-May-2021	Solvent: cdcl3	SW: 8000 Hz	TE: 298 K	AQ: 2.00 sec, RD: 0.00 sec	□

Figure S47. ^{13}C **NMR spectrum (150 MHz, DMSO-*d*$_6$) of compound 7j**

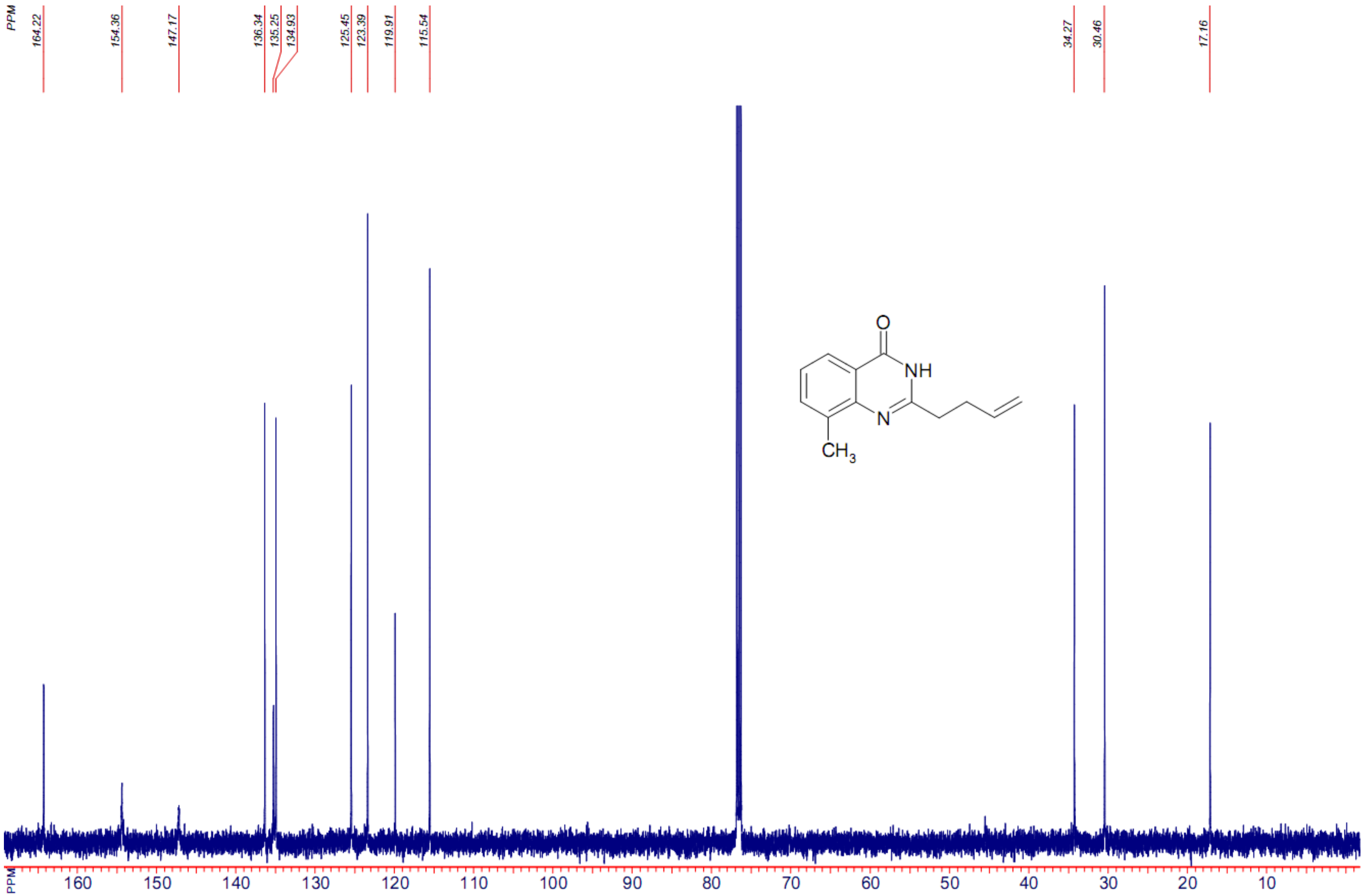

**File name: phvm94-12_C13**	Operator: root	SF: 125.6681 MHz	NSC: 400	PW: 0.00 usec, RG: 51200	SI: 131072
Date: 21-May-2021	Solvent: CDCl3	SW: 32680 Hz	TE: 683 K	AQ: 0.78 sec, RD: 0.00 sec	

Figure S48. ^{1}H NMR spectrum (400 MHz, DMSO-d_6) of compound 7k

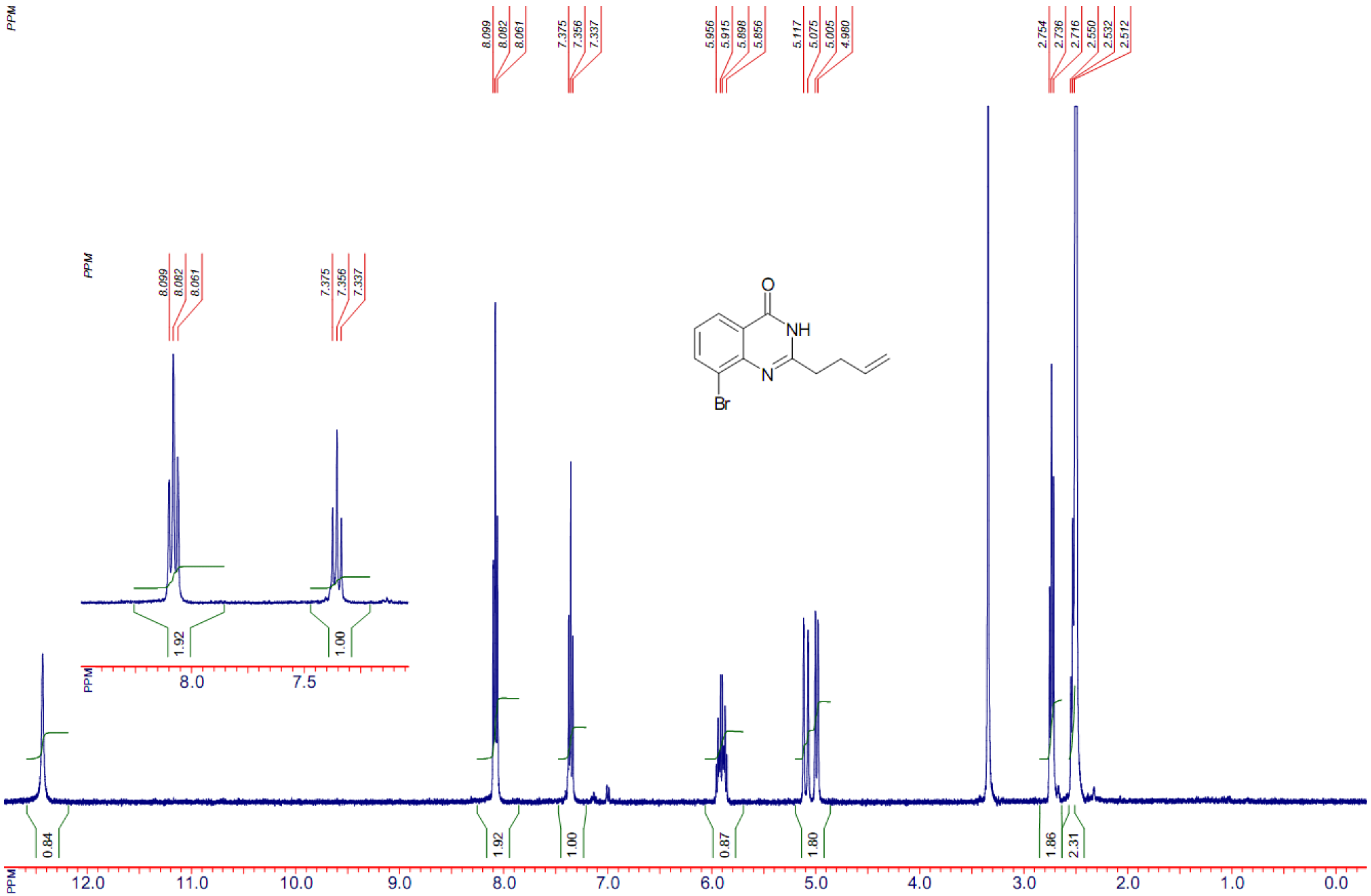

File name: PHVM1466.fid	Operator:	SF: 399.9753 MHz	NSC: 0	PW: 10.90 usec, RG: 20	SI: 32768
Date: 19-Mar-2021	Solvent: dmso	SW: 8000 Hz	TE: 298 K	AQ: 2.00 sec, RD: 0.00 sec	□

Figure S49. ^{13}C NMR spectrum (150 MHz, DMSO-d_6) of compound 7k

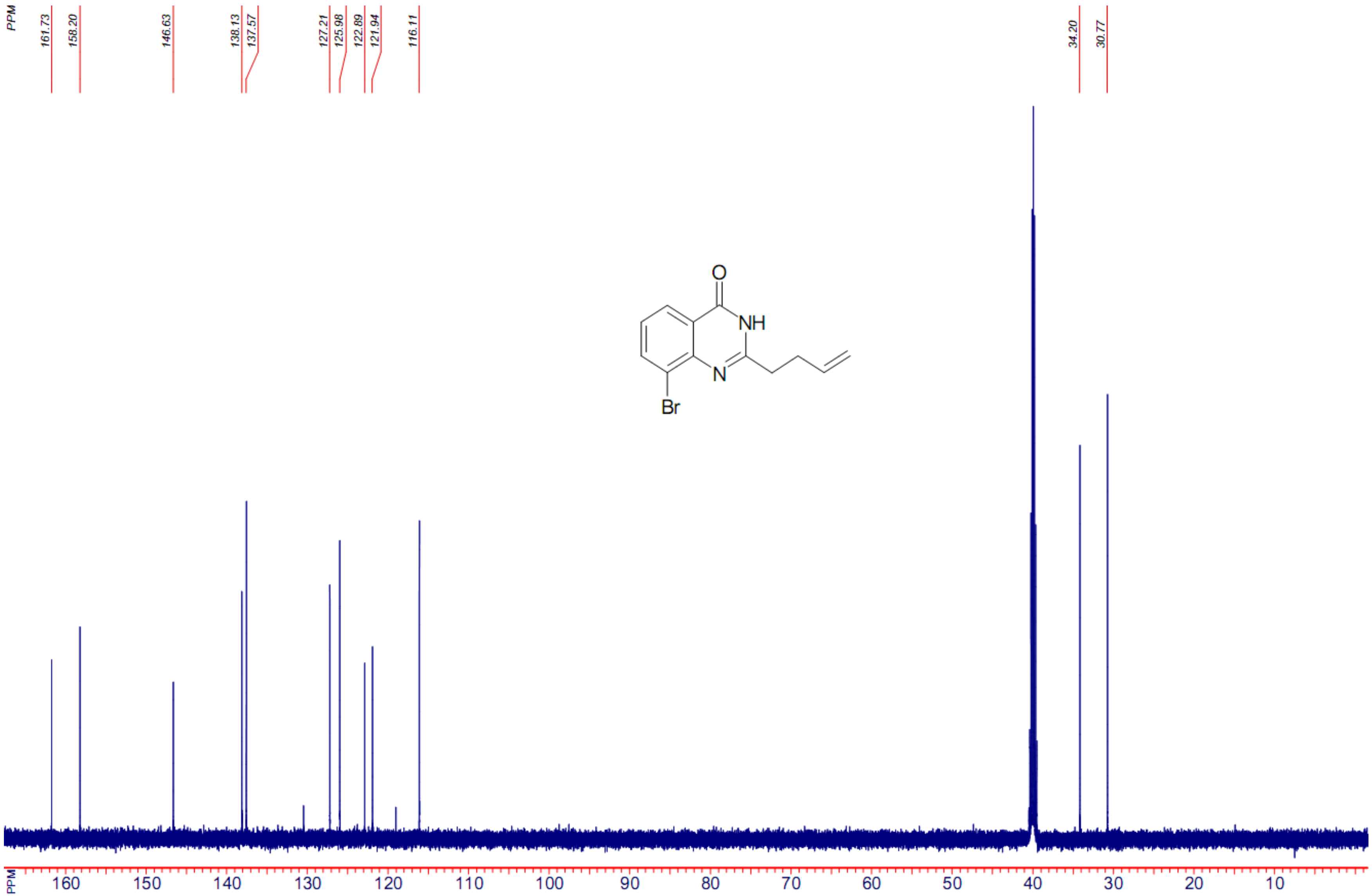

**File name: PHVM1466_C13.fid**	Operator:	SF: 150.8303 MHz	NSC: 0	PW: 3.09 usec, RG: 60	SI: 131072
Date: 23-Mar-2021	Solvent: dmso	SW: 37879 Hz	TE: 298 K	AQ: 1.73 sec, RD: 0.00 sec	□

Figure S50. ^{1}H NMR spectrum (400 MHz, DMSO-d_6) of compound 7l

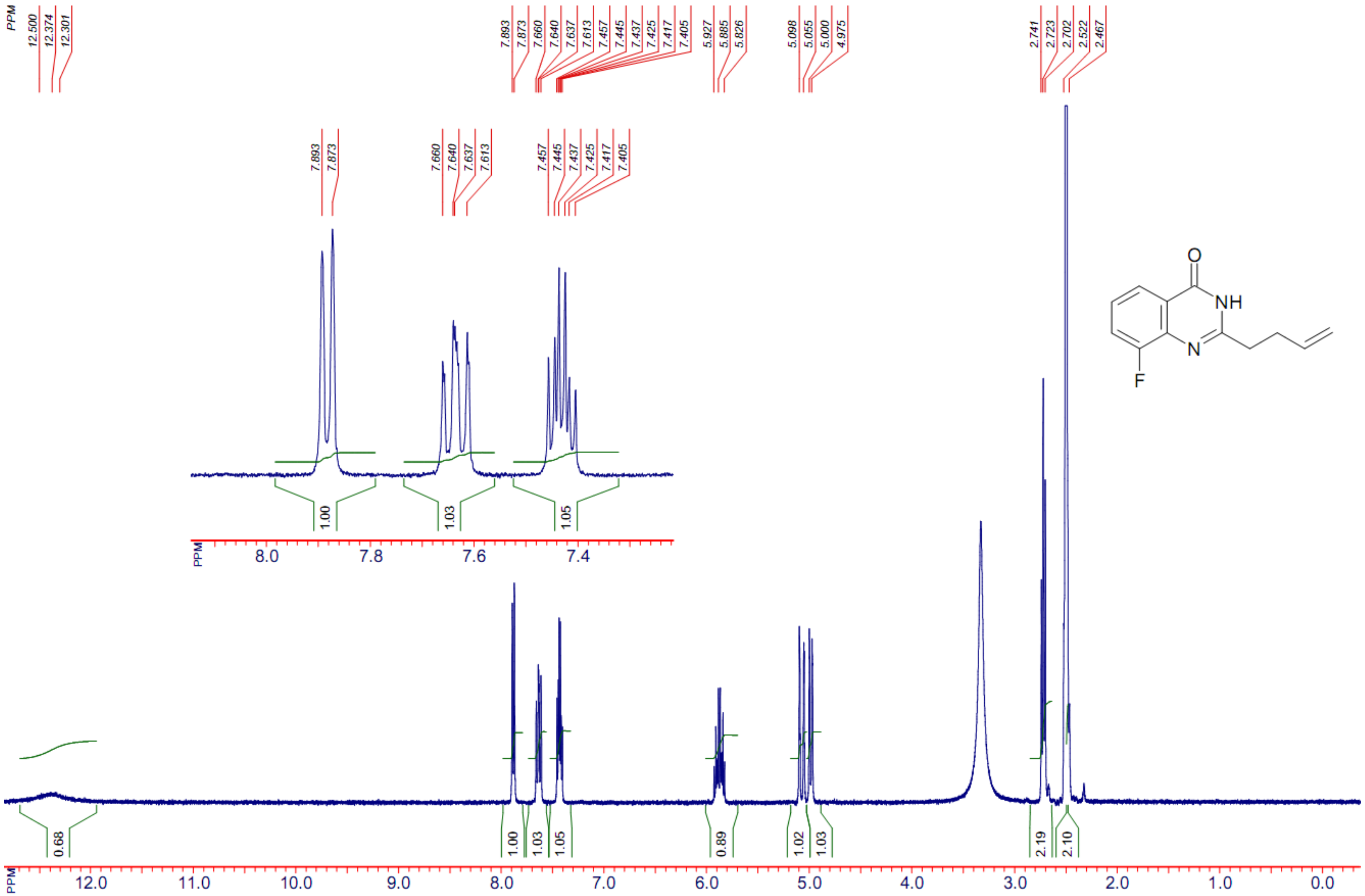

**File name: PHVM1450-3.fid**	Operator:	SF: 399.9753 MHz	NSC: 0	PW: 10.90 usec, RG: 24	SI: 32768
Date: 07-Jun-2021	Solvent: dmso	SW: 8000 Hz	TE: 298 K	AQ: 2.00 sec, RD: 0.00 sec	□

Figure S51. ^{19}F NMR spectrum (188 MHz, DMSO-d_6) of compound 7l

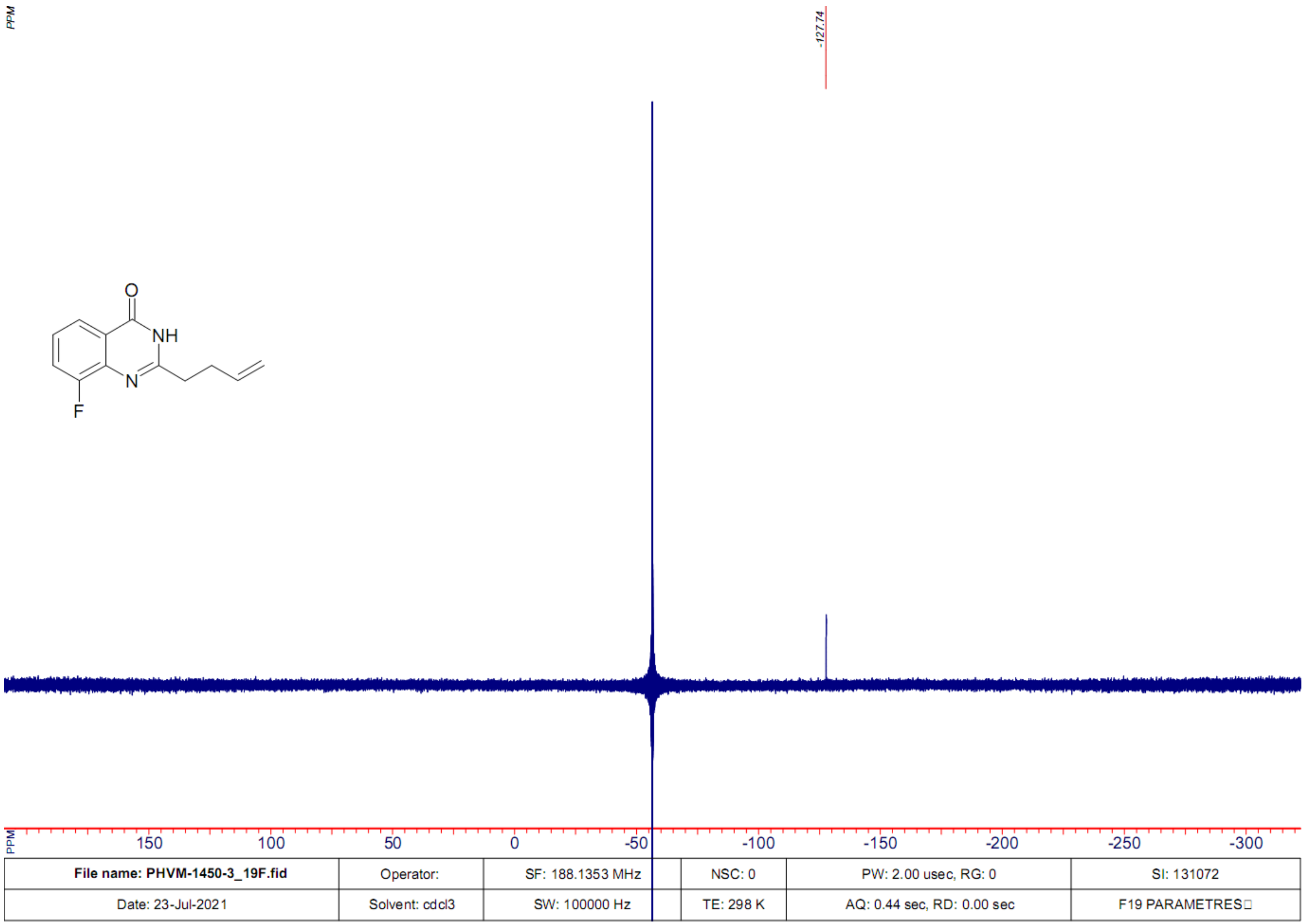

**File name: PHVM-1450-3_19F.fid**	Operator:	SF: 188.1353 MHz	NSC: 0	PW: 2.00 usec, RG: 0	SI: 131072
Date: 23-Jul-2021	Solvent: cdcl3	SW: 100000 Hz	TE: 298 K	AQ: 0.44 sec, RD: 0.00 sec	F19 PARAMETRES

Figure S52. **^{13}C NMR spectrum (150 MHz, DMSO-d_6) of compound 7l**

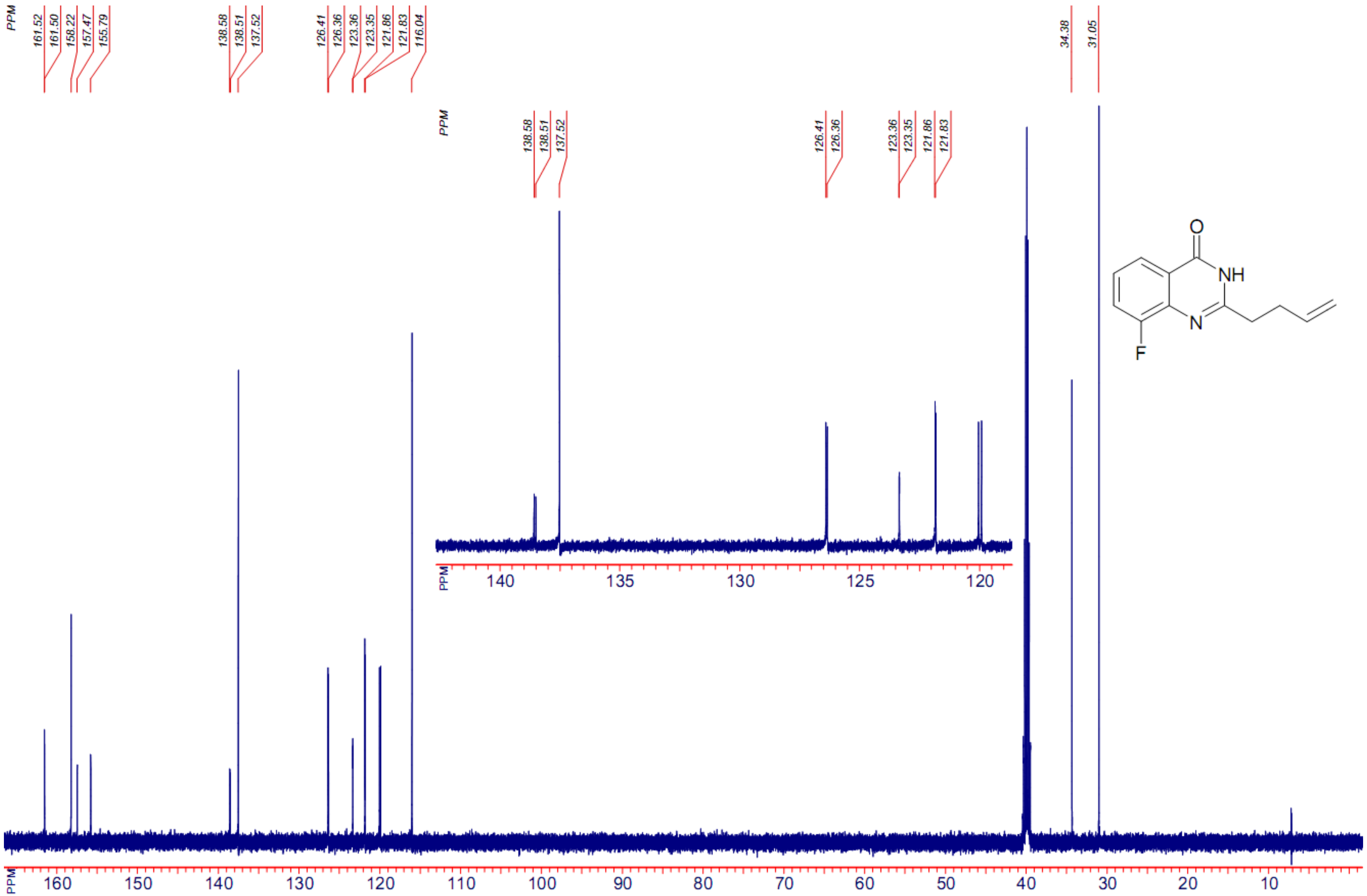

File name: phvm1450-3_C13.fid	Operator:	SF: 150.8305 MHz	NSC: 0	PW: 3.09 usec, RG: 60	SI: 131072
Date: 09-Jun-2021	Solvent: dmso	SW: 37879 Hz	TE: 298 K	AQ: 1.73 sec, RD: 0.00 sec	□

Figure S53. ^{1}H **NMR spectrum (400 MHz, DMSO-*d*$_6$) of compound 6a**

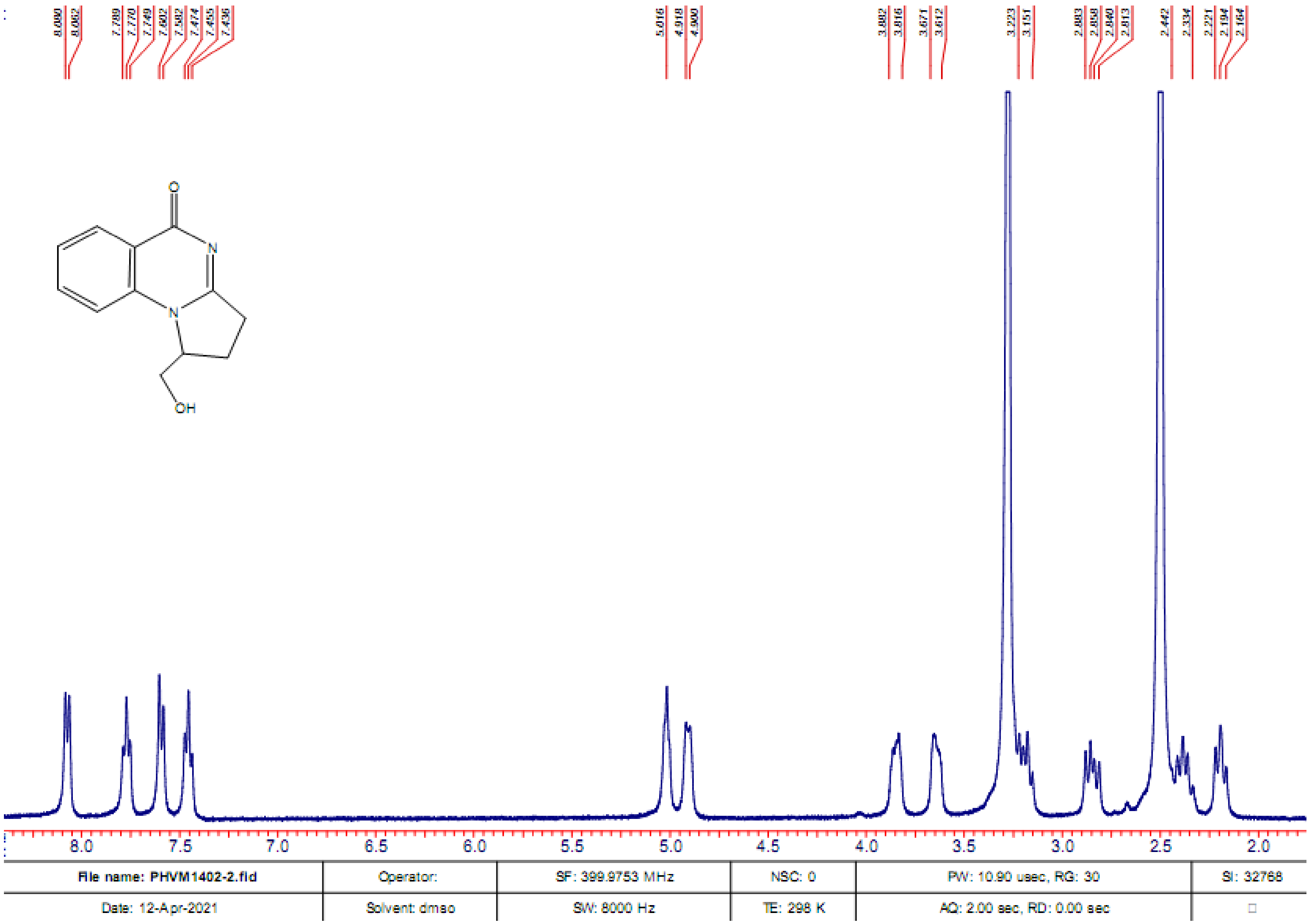

Figure S54. ^{13}C NMR spectrum (125 MHz, DMSO-d_6) of compound 6a

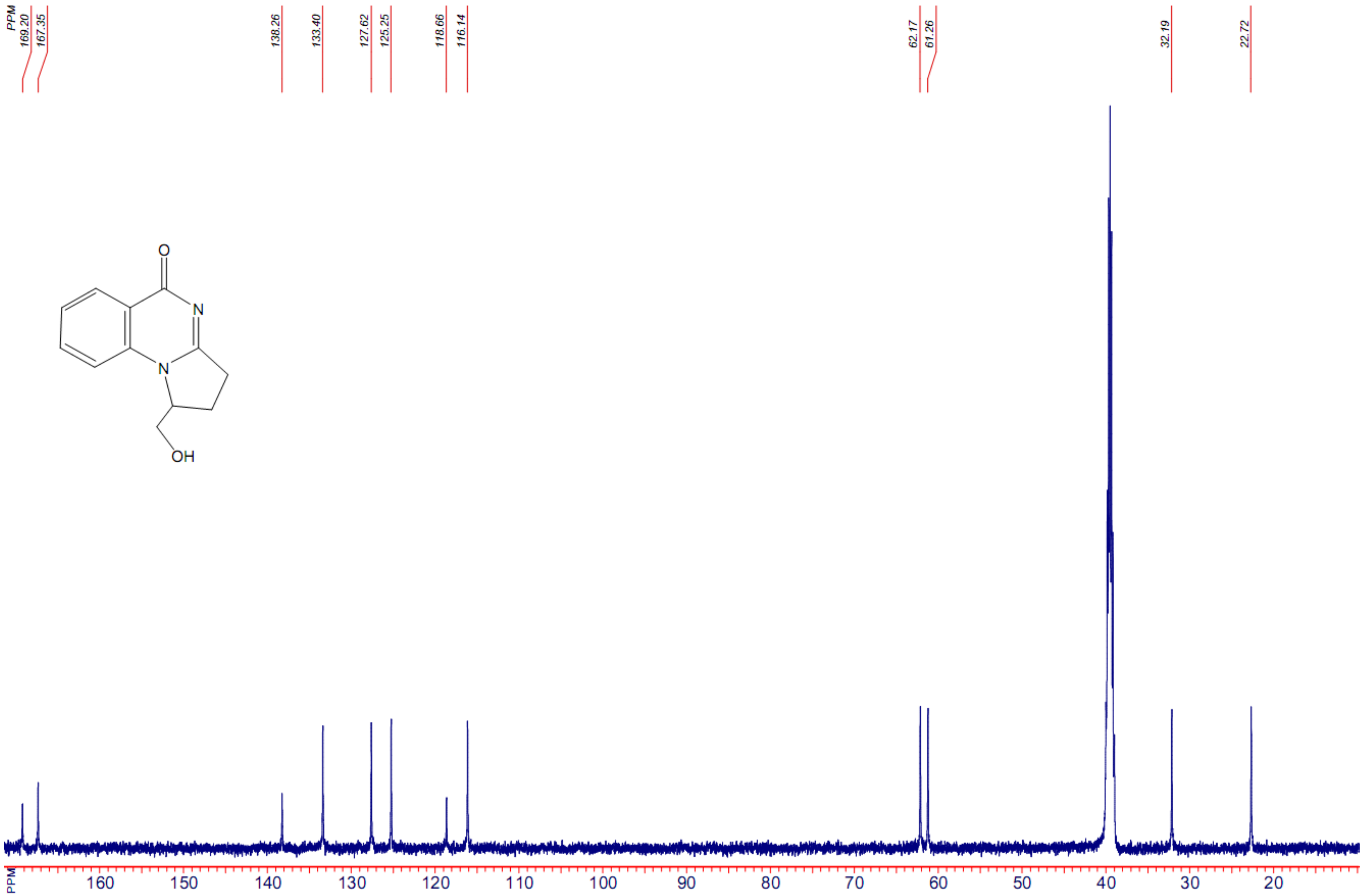

**File name: PHvm1402-2___13C**	Operator: root□	SF: 125.6681 MHz	NSC: 520	PW: 0.00 usec, RG: 51200	SI: 131072
Date: 14-Apr-2021	Solvent: DMSO	SW: 32680 Hz	TE: 683 K	AQ: 1.00 sec, RD: 0.00 sec	□

Figure S55. ^{1}**H NMR spectrum (400 MHz, DMSO-d_6) of compound 6b**

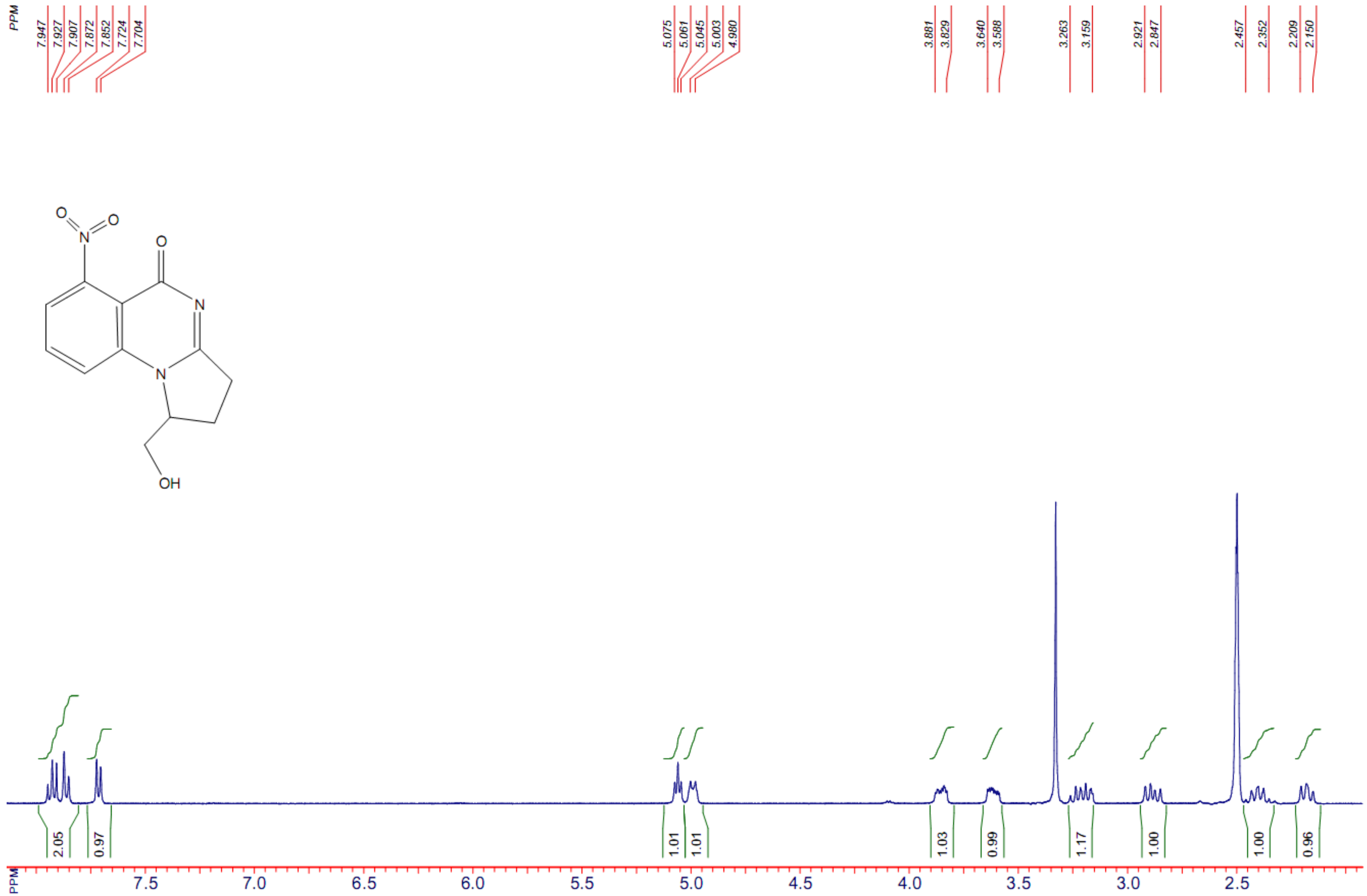

**File name: PHVM1429-21.fid**	Operator:	SF: 399.9753 MHz	NSC: 0	PW: 10.90 usec, RG: 24	SI: 32768
Date: 31-May-2021	Solvent: dmso	SW: 8000 Hz	TE: 298 K	AQ: 2.00 sec, RD: 0.00 sec	□

Figure S56. ^{13}C NMR spectrum (150 MHz, DMSO-d_6) of compound 6b

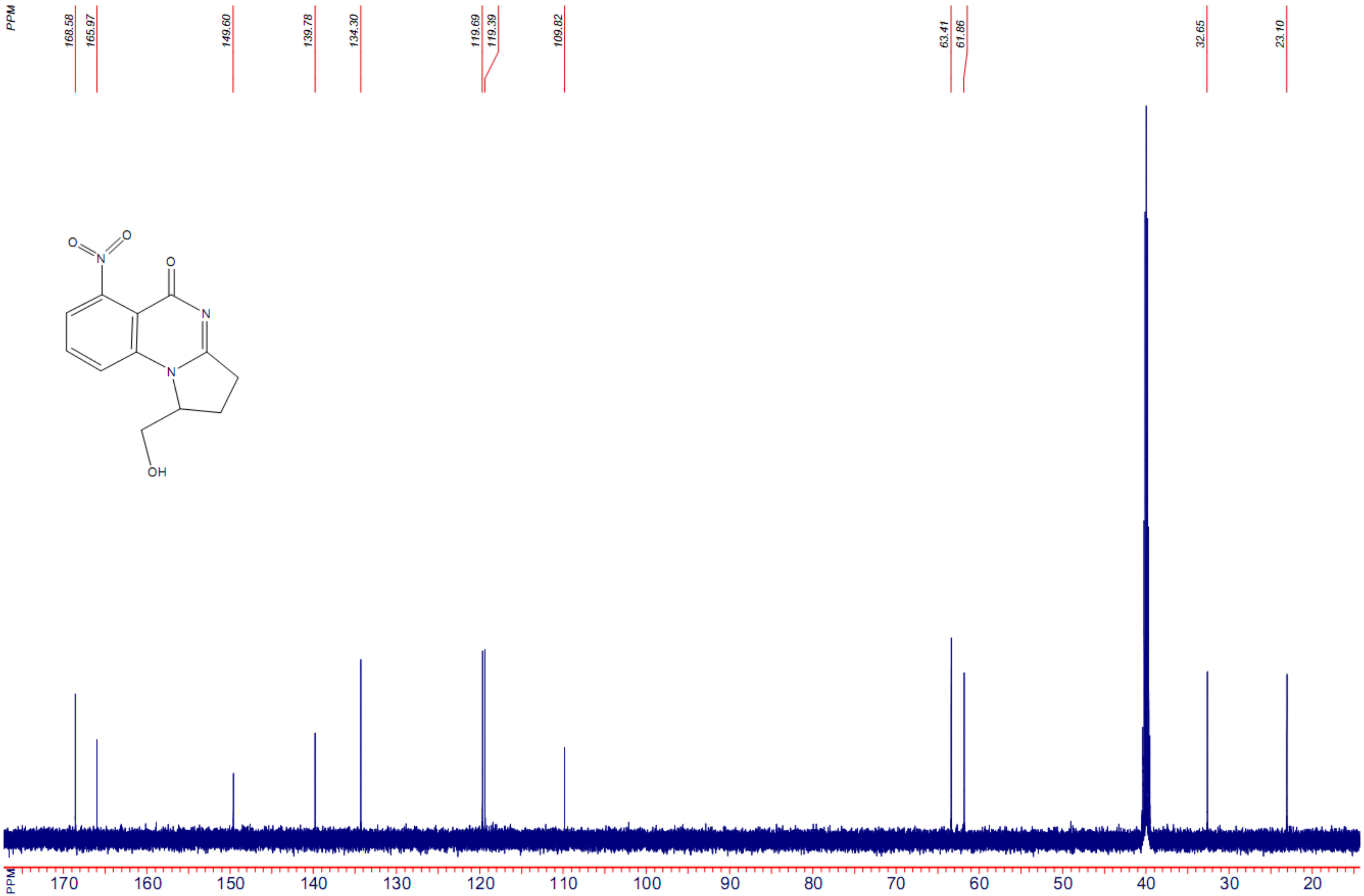

**File name: PHVM1429-21_C13.fid**	Operator:	SF: 150.8299 MHz	NSC: 0	PW: 3.09 usec, RG: 60	SI: 131072
Date: 01-Jun-2021	Solvent: dmso	SW: 37879 Hz	TE: 298 K	AQ: 1.73 sec, RD: 0.00 sec	□

Figure S57. ^{1}H NMR spectrum (400 MHz, DMSO-*d*$_6$) of compound 6c

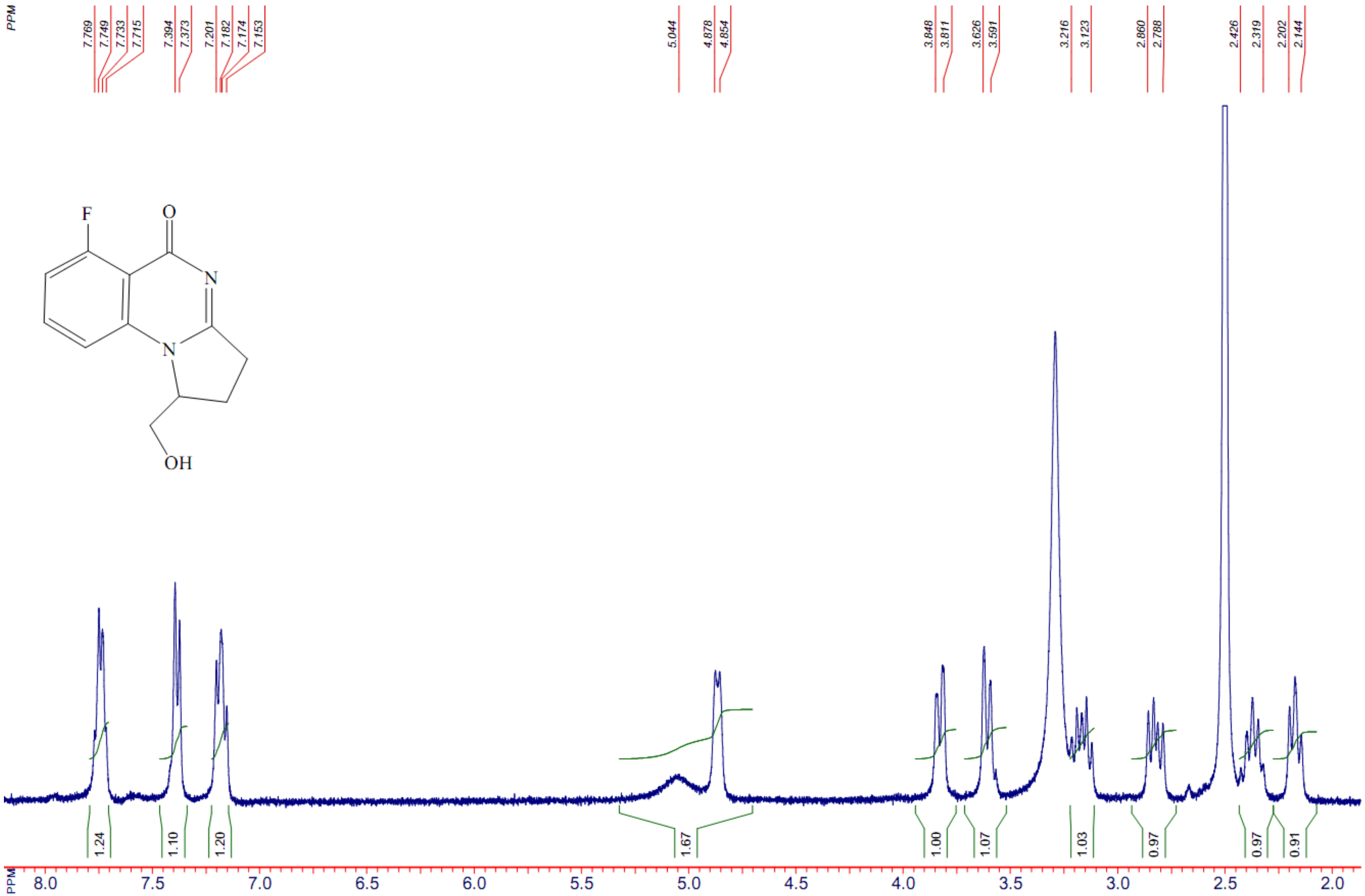

**File name: PHVM1417-1.fid**	Operator:	SF: 399.9753 MHz	NSC: 0	PW: 10.90 usec, RG: 24	SI: 32768
Date: 11-May-2021	Solvent: dmso	SW: 8000 Hz	TE: 298 K	AQ: 2.00 sec, RD: 0.00 sec	□

Figure S58. ^{19}F NMR spectrum (188 MHz, DMSO-*d*6) of compound 6c

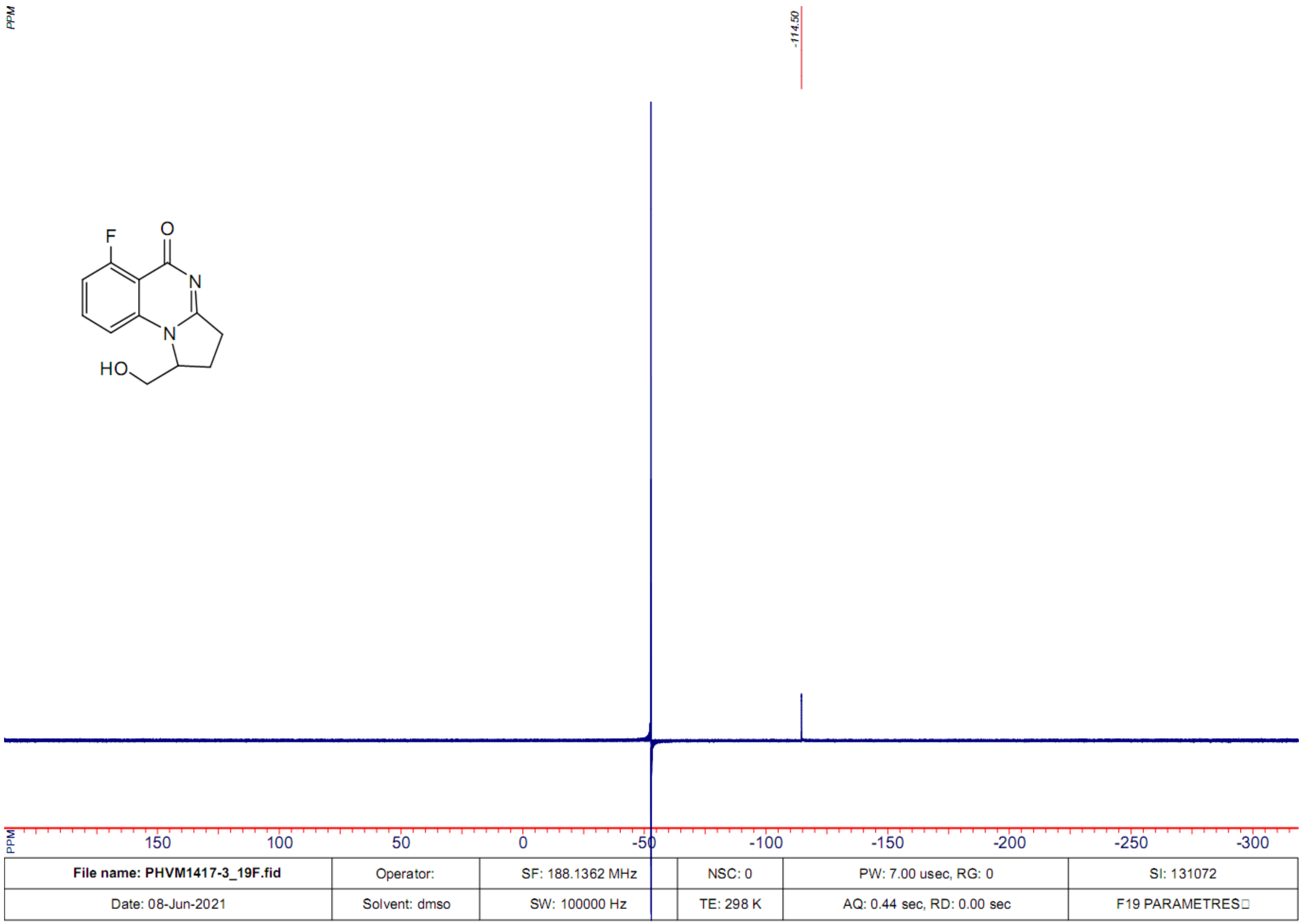

**File name: PHVM1417-3_19F.fid**	Operator:	SF: 188.1362 MHz	NSC: 0	PW: 7.00 usec, RG: 0	SI: 131072
Date: 08-Jun-2021	Solvent: dmso	SW: 100000 Hz	TE: 298 K	AQ: 0.44 sec, RD: 0.00 sec	F19 PARAMETRES□

Figure S59. ^{1}H NMR spectrum (400 MHz, DMSO-d_6) of compound 6d

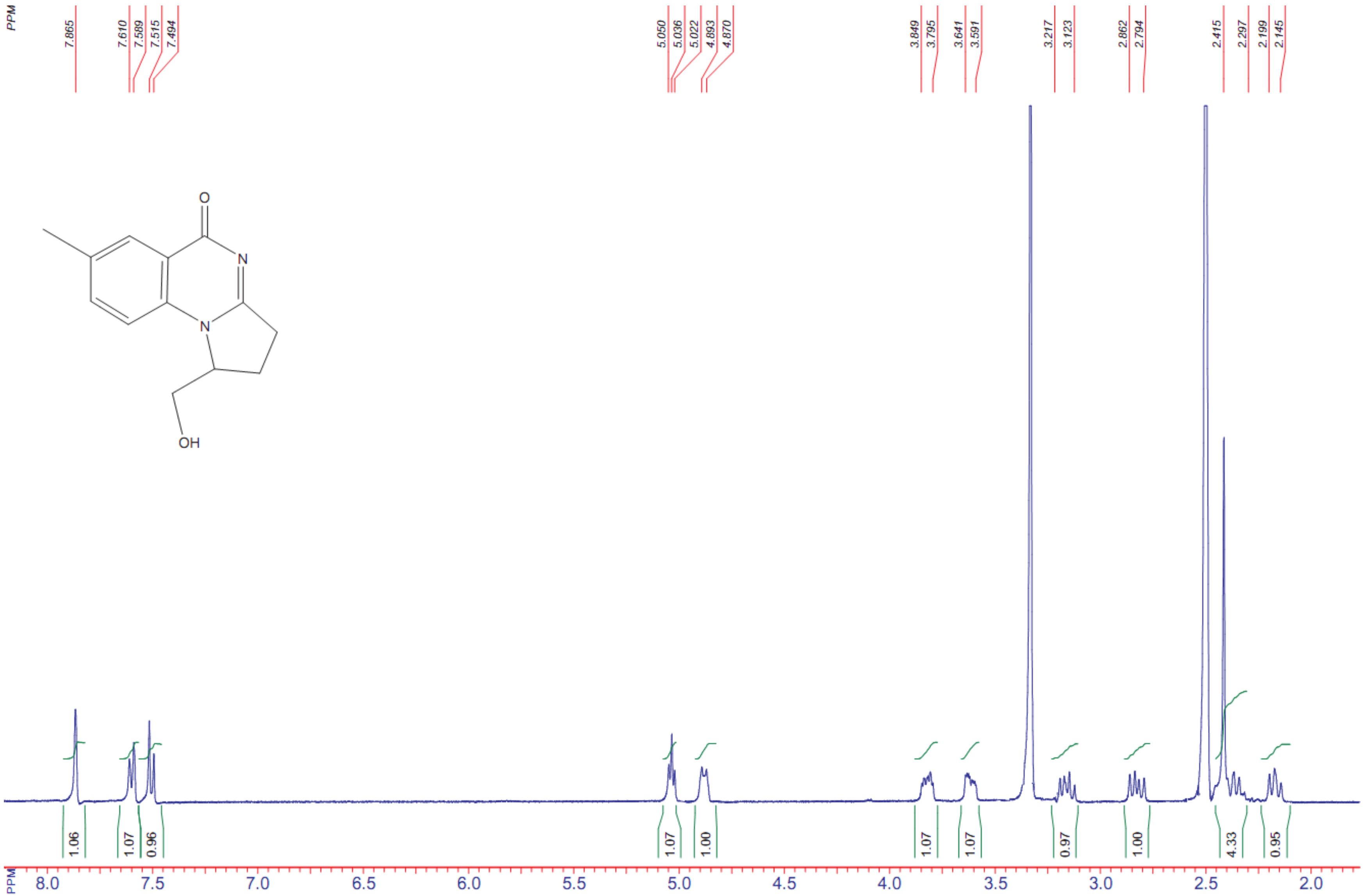

**File name: PHVM1444-2.fid**	Operator:	SF: 399.9753 MHz	NSC: 0	PW: 10.90 usec, RG: 24	SI: 32768
Date: 16-Jun-2021	Solvent: dmso	SW: 8000 Hz	TE: 298 K	AQ: 2.00 sec, RD: 0.00 sec	

Figure S60. ^{13}C NMR spectrum (150 MHz, DMSO-d_6) of compound 6d

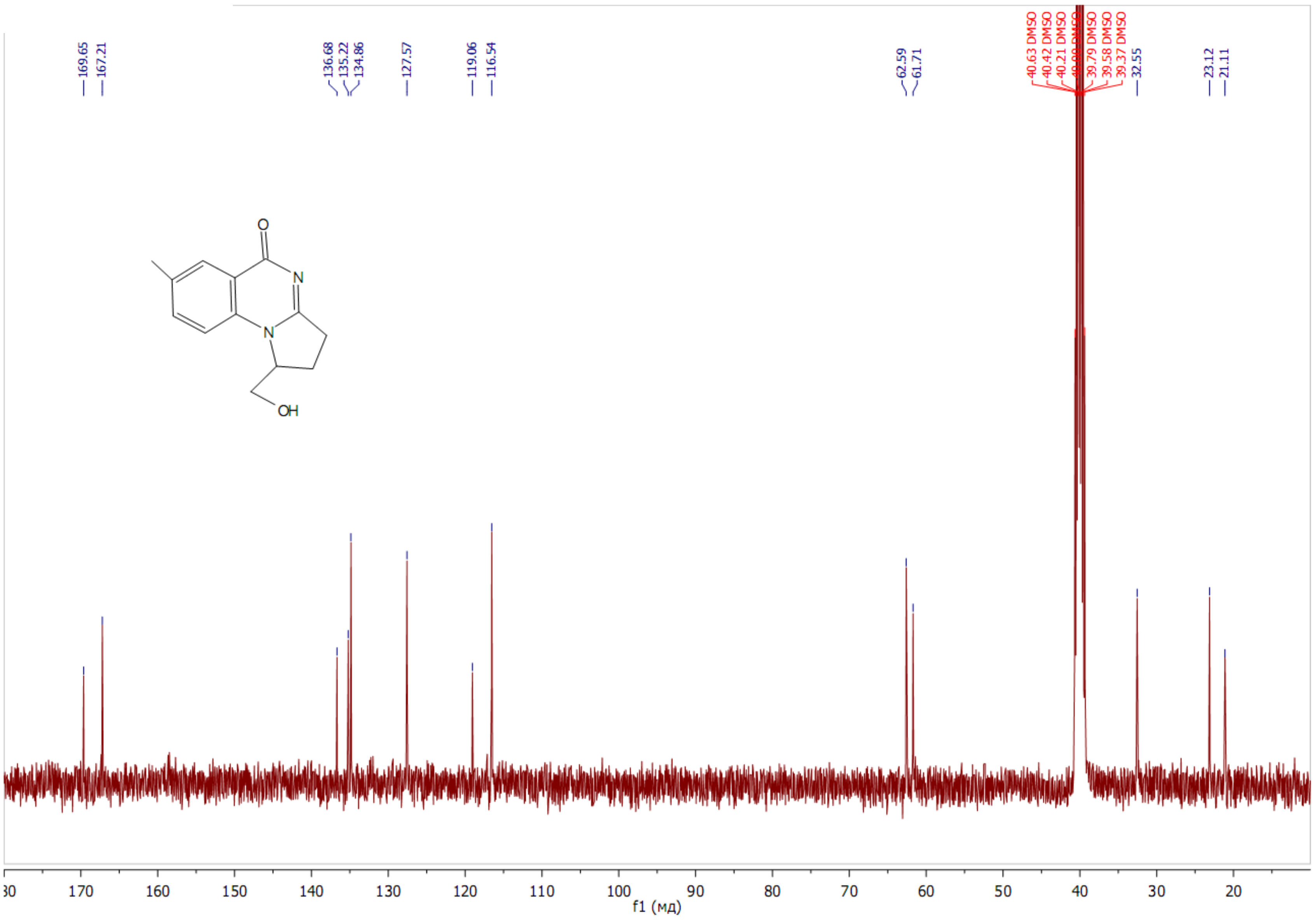

Figure S61. ^{1}H NMR spectrum (400 MHz, DMSO-d_6) of compound 6e

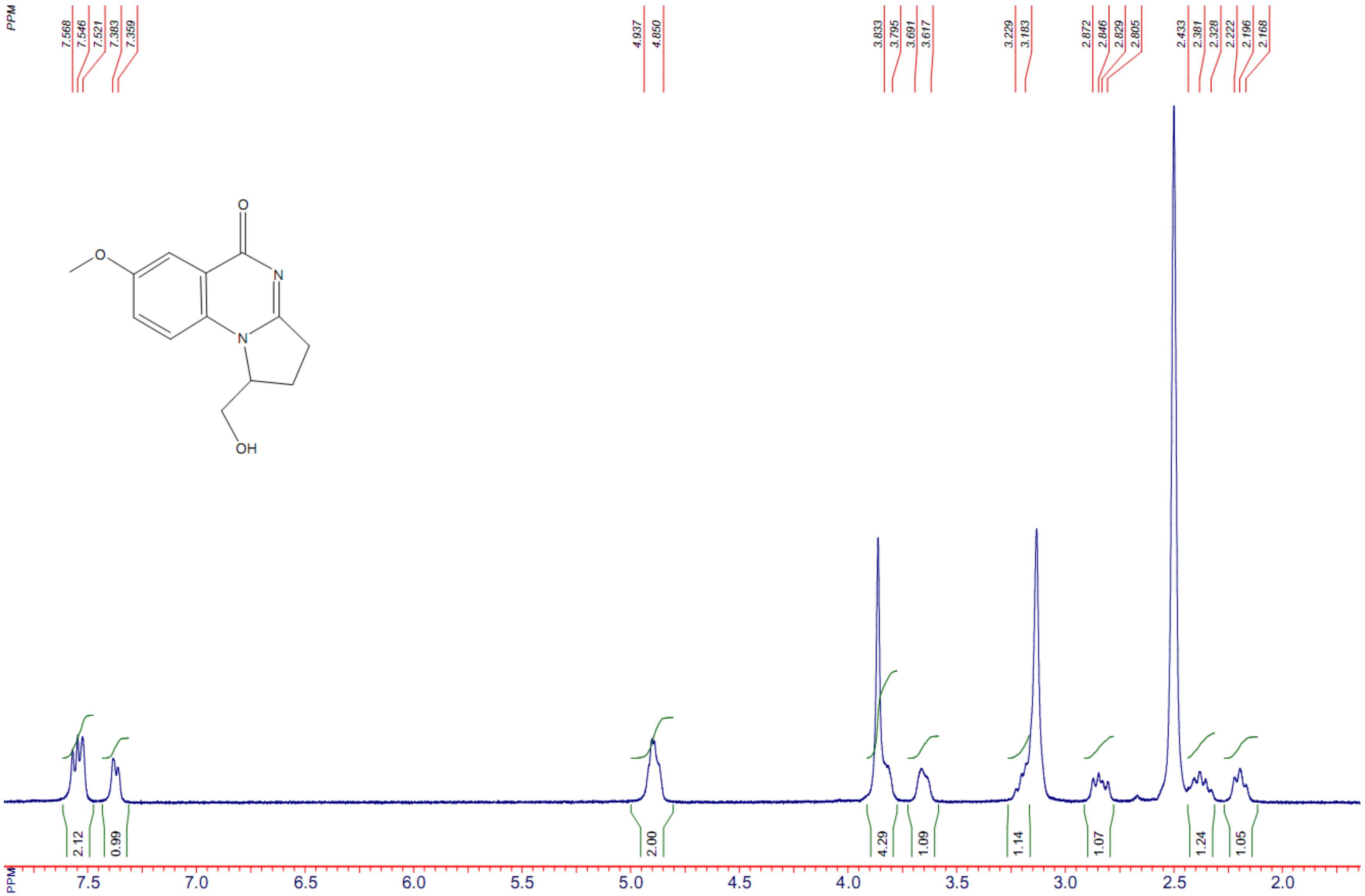

**File name: PHvm1403-11.fid**	Operator:	SF: 399.9753 MHz	NSC: 0	PW: 10.90 usec, RG: 24	SI: 32768
Date: 26-Apr-2021	Solvent: dmso	SW: 8000 Hz	TE: 298 K	AQ: 2.00 sec, RD: 0.00 sec	□

Figure S62. ^{13}C NMR spectrum (125 MHz, DMSO-d_6) of compound 6e

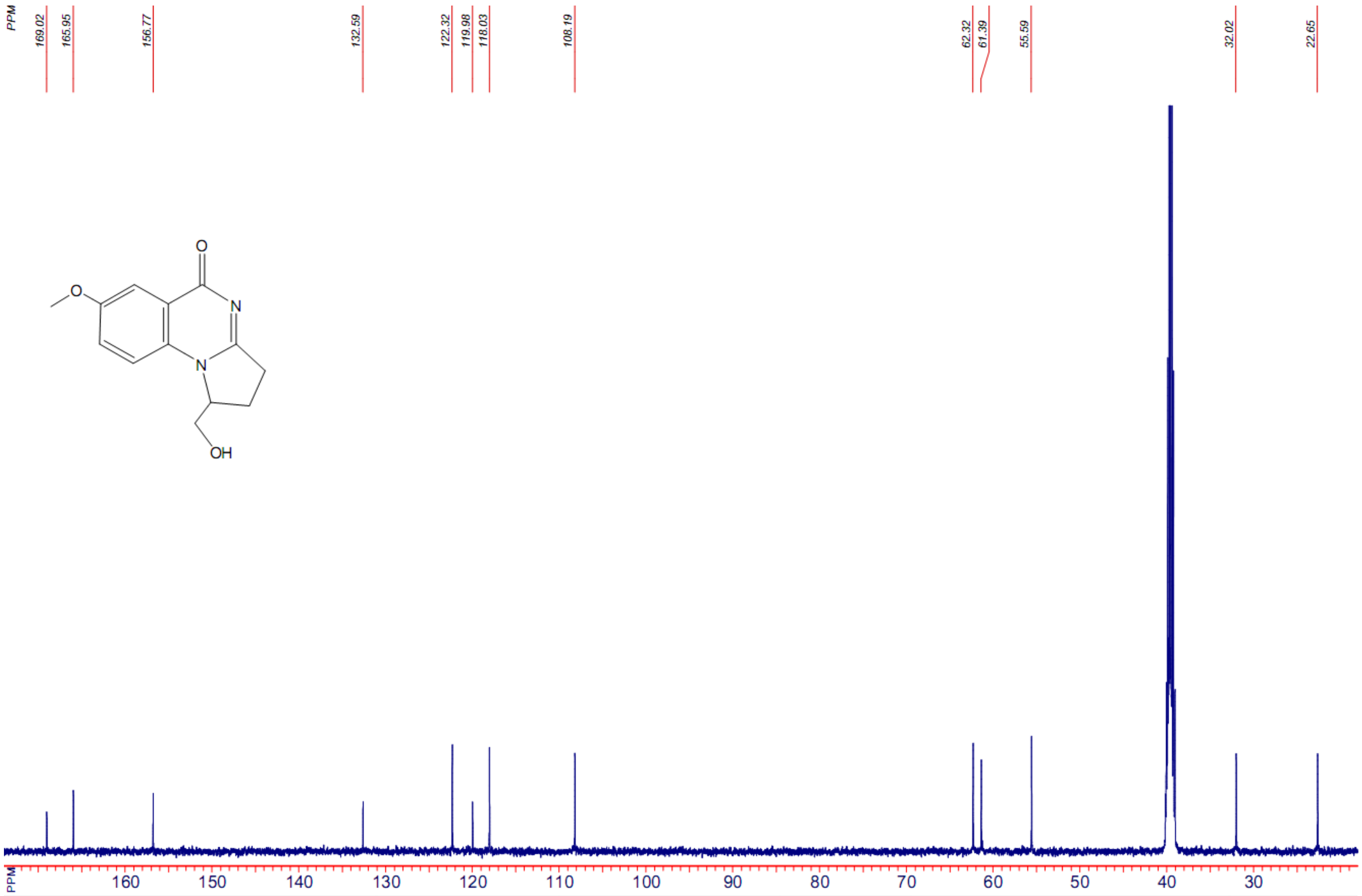

**File name: PHvm1403-11___13C**	Operator: root□	SF: 125.6681 MHz	NSC: 1344	PW: 0.00 usec, RG: 51200	SI: 131072
Date: 30-Apr-2021	Solvent: DMSO	SW: 32680 Hz	TE: 683 K	AQ: 1.00 sec, RD: 0.00 sec	□

Figure S63. ^{1}H NMR spectrum (400 MHz, DMSO-*d*6) of compound 6f

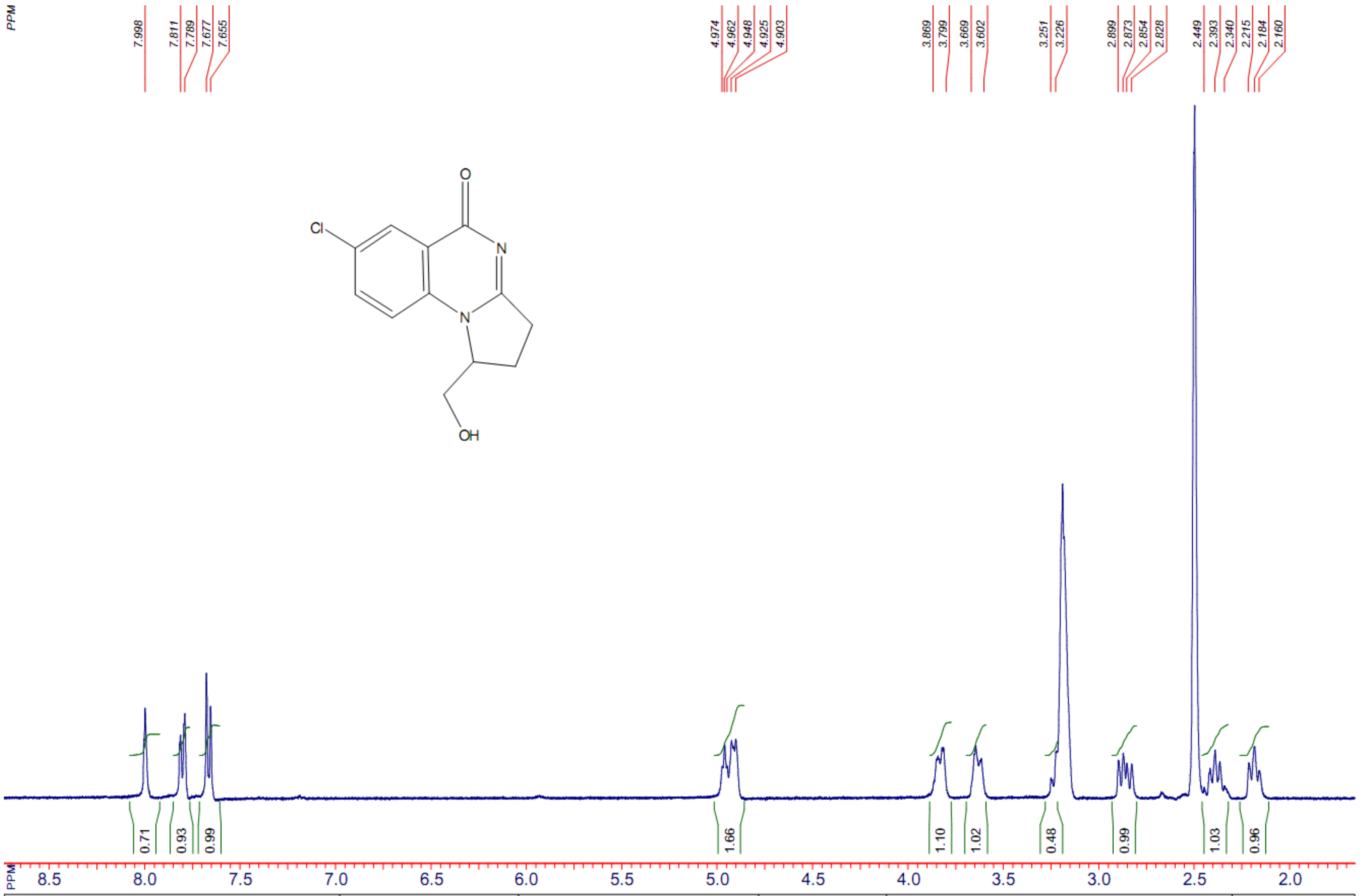

**File name: PHvm1366-1.fid**	Operator:	SF: 399.9755 MHz	NSC: 0	PW: 11.60 usec, RG: 24	SI: 32768
Date: 01-Mar-2021	Solvent: dmso	SW: 7599 Hz	TE: 294 K	AQ: 2.11 sec, RD: 0.00 sec	□

Figure S64. ^{13}C NMR spectrum (125 MHz, DMSO-d_6) of compound 6f

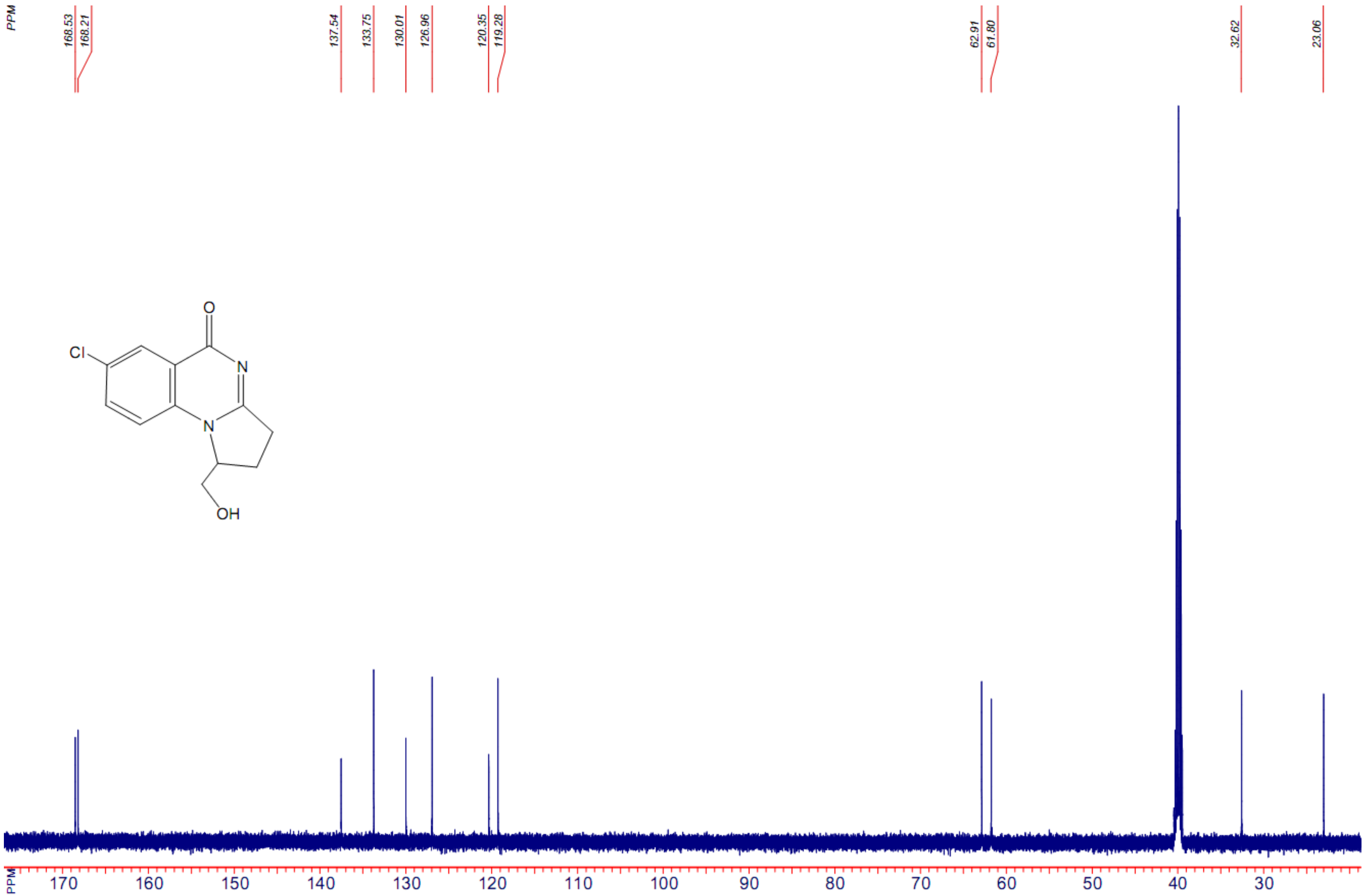

**File name: PHvm1366-1_C13.fid**	Operator:	SF: 150.8305 MHz	NSC: 0	PW: 3.09 usec, RG: 60	SI: 131072
Date: 18-Apr-2021	Solvent: dmso	SW: 37879 Hz	TE: 298 K	AQ: 1.73 sec, RD: 0.00 sec	□

Figure S65. ^{1}H NMR spectrum (400 MHz, DMSO-d_6) of compound 6g

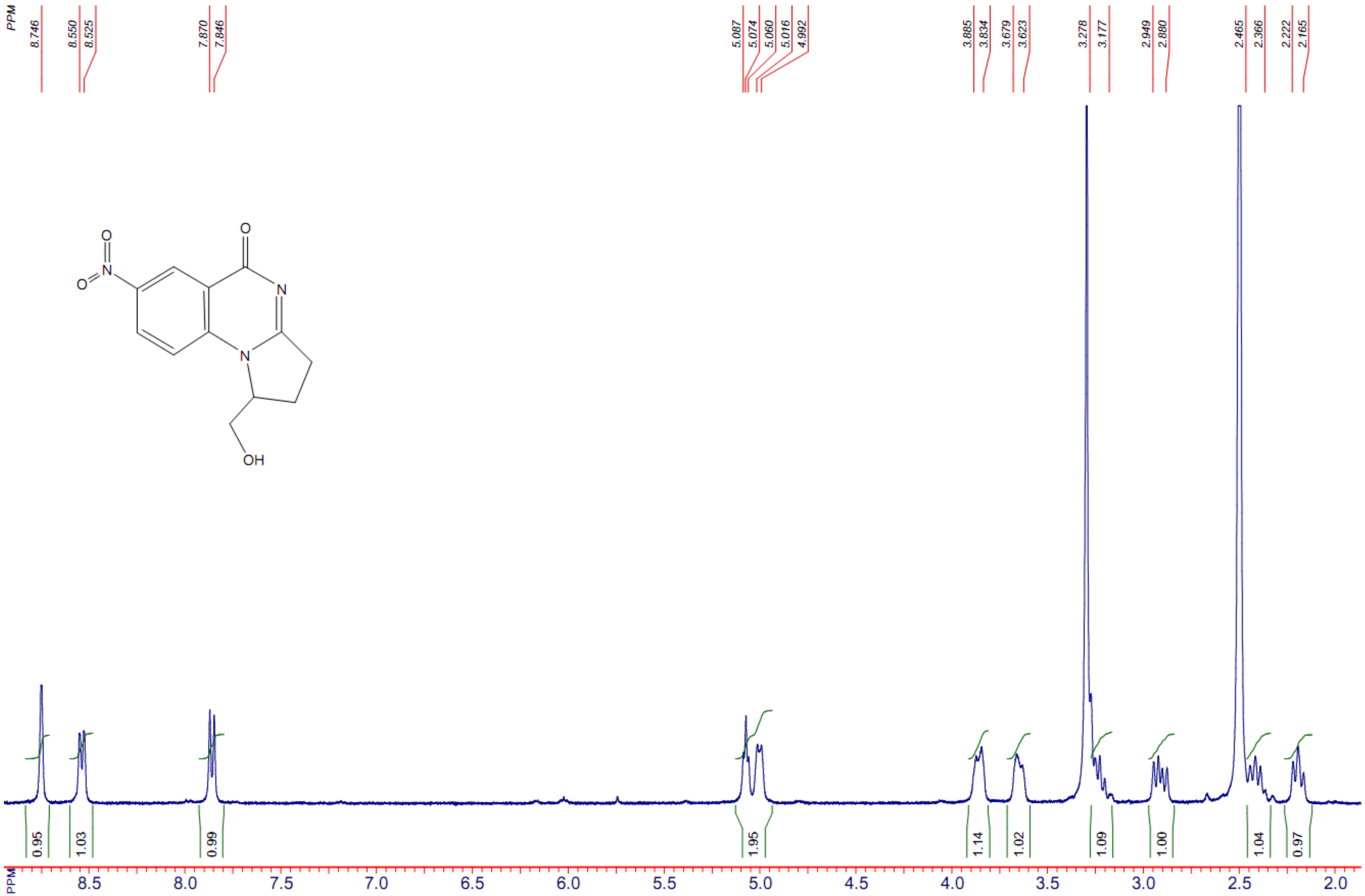

**File name: PHvm1367-2.fid**	Operator:	SF: 399.9753 MHz	NSC: 0	PW: 10.90 usec, RG: 24	SI: 32768
Date: 16-Mar-2021	Solvent: dmso	SW: 8000 Hz	TE: 298 K	AQ: 2.00 sec, RD: 0.00 sec	□

Figure S66. ^{13}C NMR spectrum (150 MHz, DMSO-d_6) of compound 6g

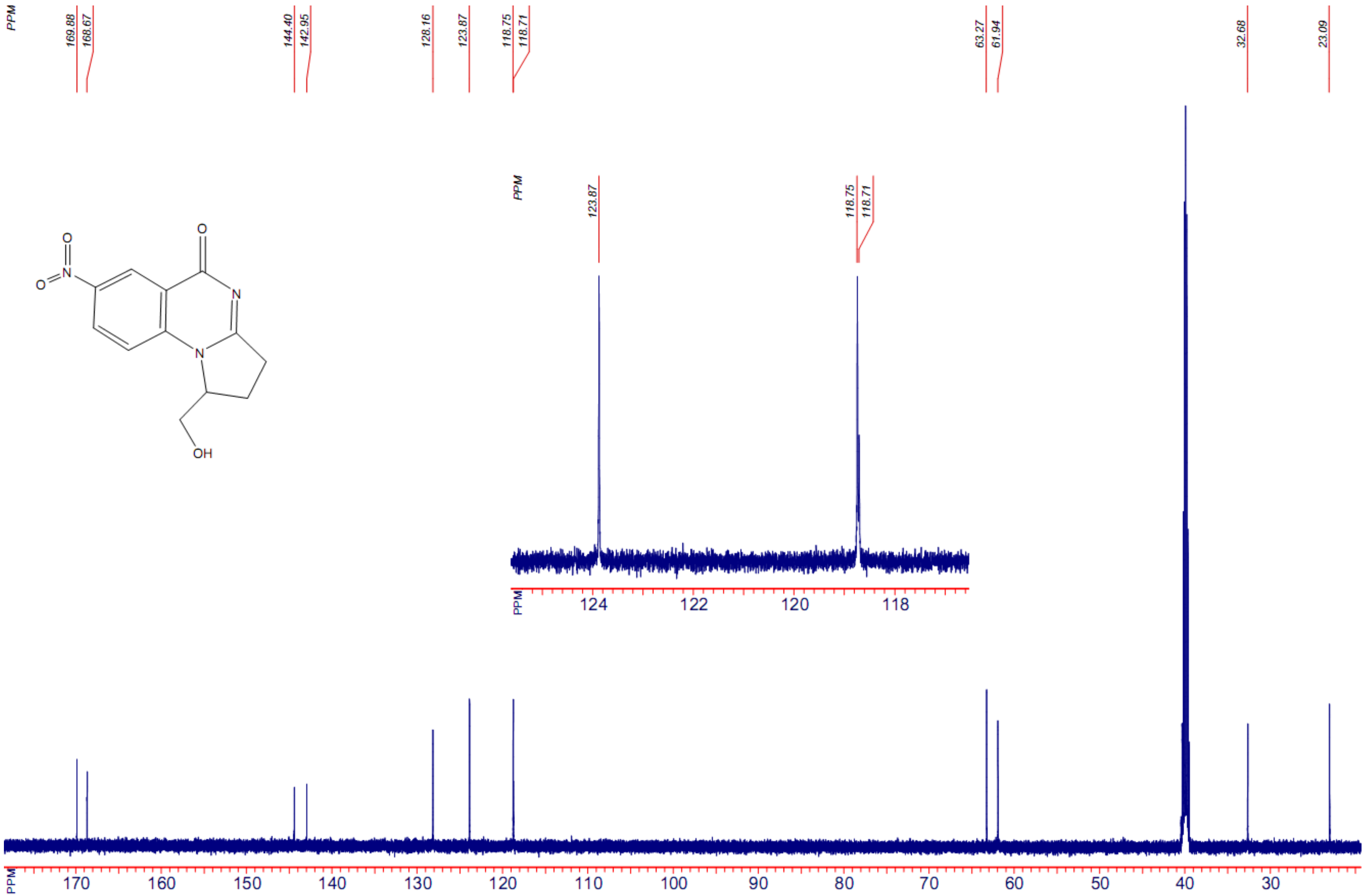

File name: PHvm1367-2_C13.fid	Operator:	SF: 150.8305 MHz	NSC: 0	PW: 3.09 usec, RG: 60	SI: 131072
Date: 18-Apr-2021	Solvent: dmso	SW: 37879 Hz	TE: 298 K	AQ: 1.73 sec, RD: 0.00 sec	□

Figure S67. ^{1}H NMR spectrum (400 MHz, DMSO-d_6) of compound 6h

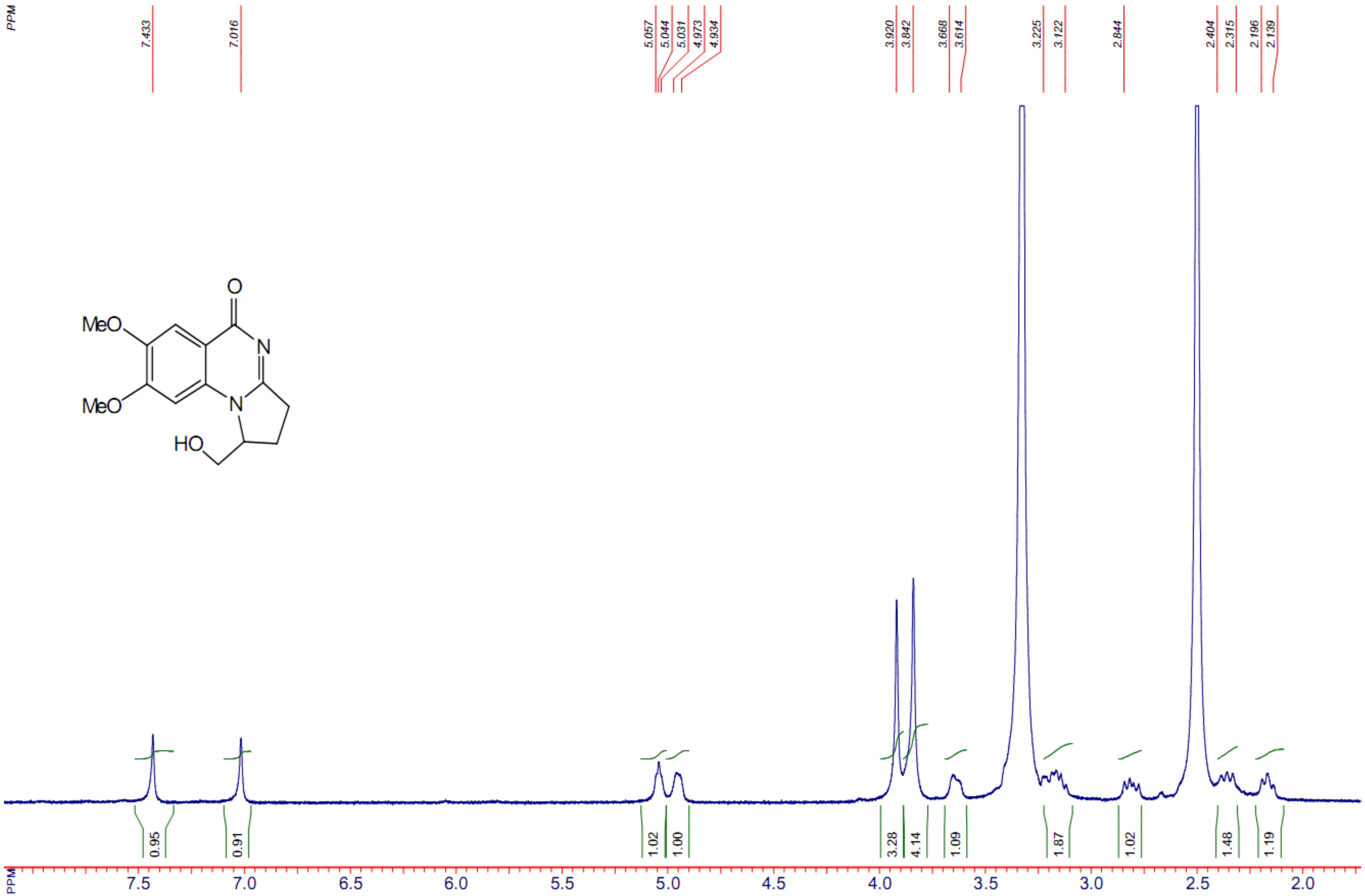

**File name: PHvm1459.fid**	Operator:	SF: 399.9753 MHz	NSC: 0	PW: 10.90 usec, RG: 24	SI: 32768
Date: 23-Jun-2021	Solvent: dmso	SW: 8000 Hz	TE: 298 K	AQ: 2.00 sec, RD: 0.00 sec	□

Figure S68. ^{13}C NMR spectrum (125 MHz, DMSO-d_6) of compound 6h

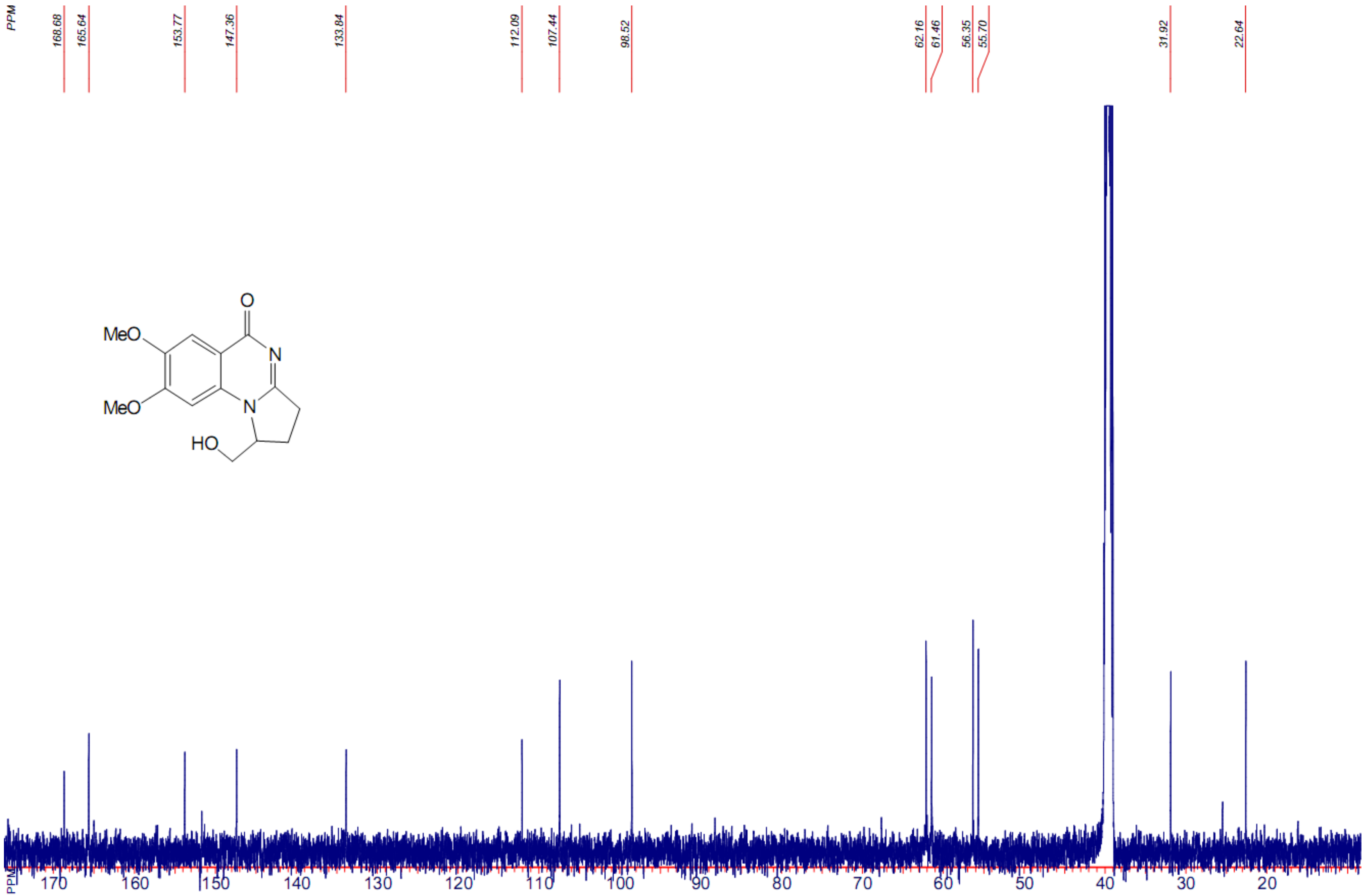

File name: PHvm1427___13C	Operator: root	SF: 125.6681 MHz	NSC: 1273	PW: 0.00 usec, RG: 51200	SI: 131072
Date: 25-May-2021	Solvent: DMSO	SW: 32680 Hz	TE: 683 K	AQ: 1.00 sec, RD: 0.00 sec	

Figure S69. ^{1}H NMR spectrum (400 MHz, DMSO-d_6) of compound 6i

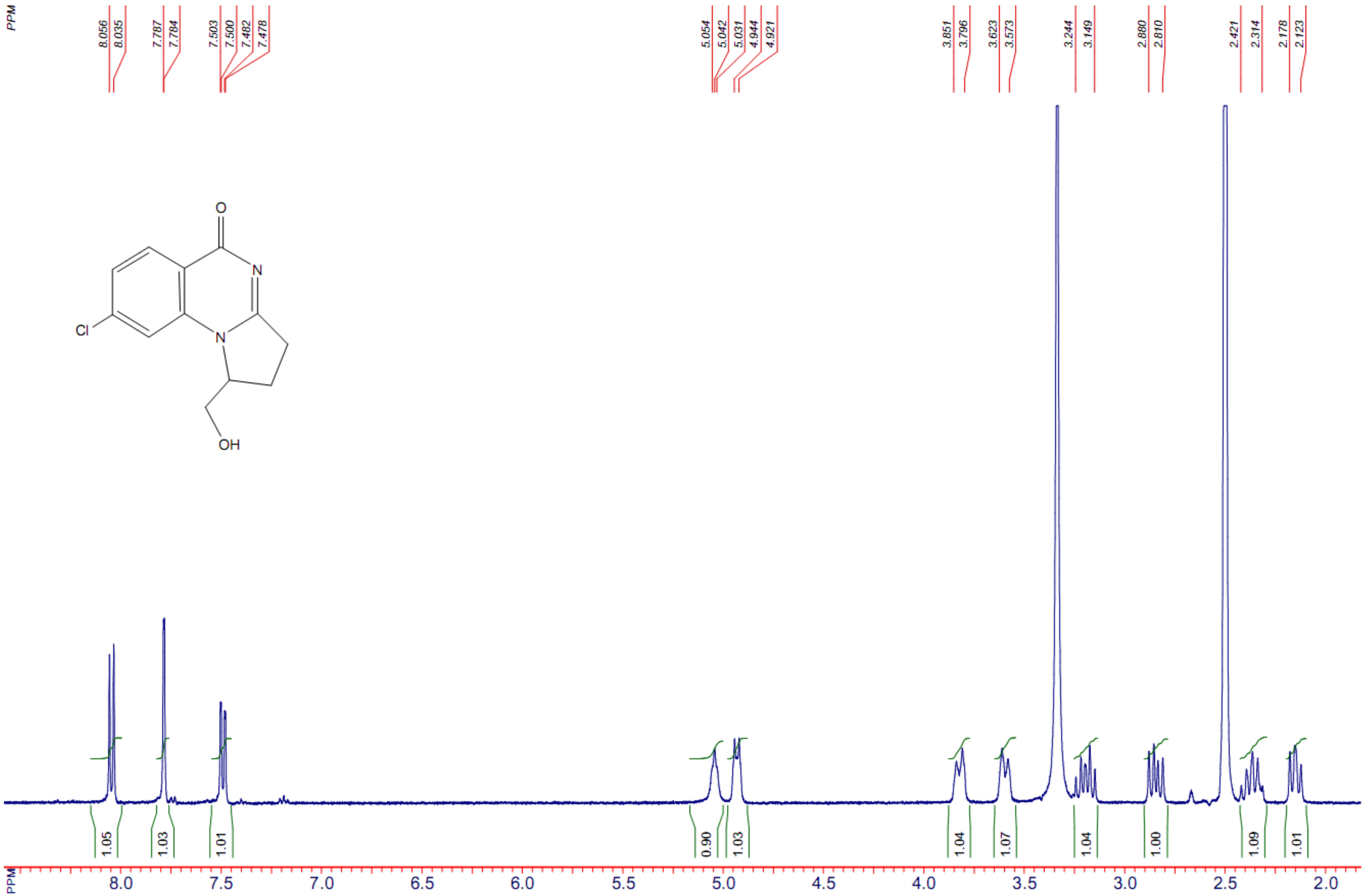

**File name: PHvm1426-6.fid**	Operator:	SF: 399.9753 MHz	NSC: 0	PW: 10.90 usec, RG: 24	SI: 32768
Date: 13-May-2021	Solvent: dmso	SW: 8000 Hz	TE: 298 K	AQ: 2.00 sec, RD: 0.00 sec	□

Figure S70. ^{13}C NMR spectrum (125 MHz, DMSO-d_6) of compound **6i**

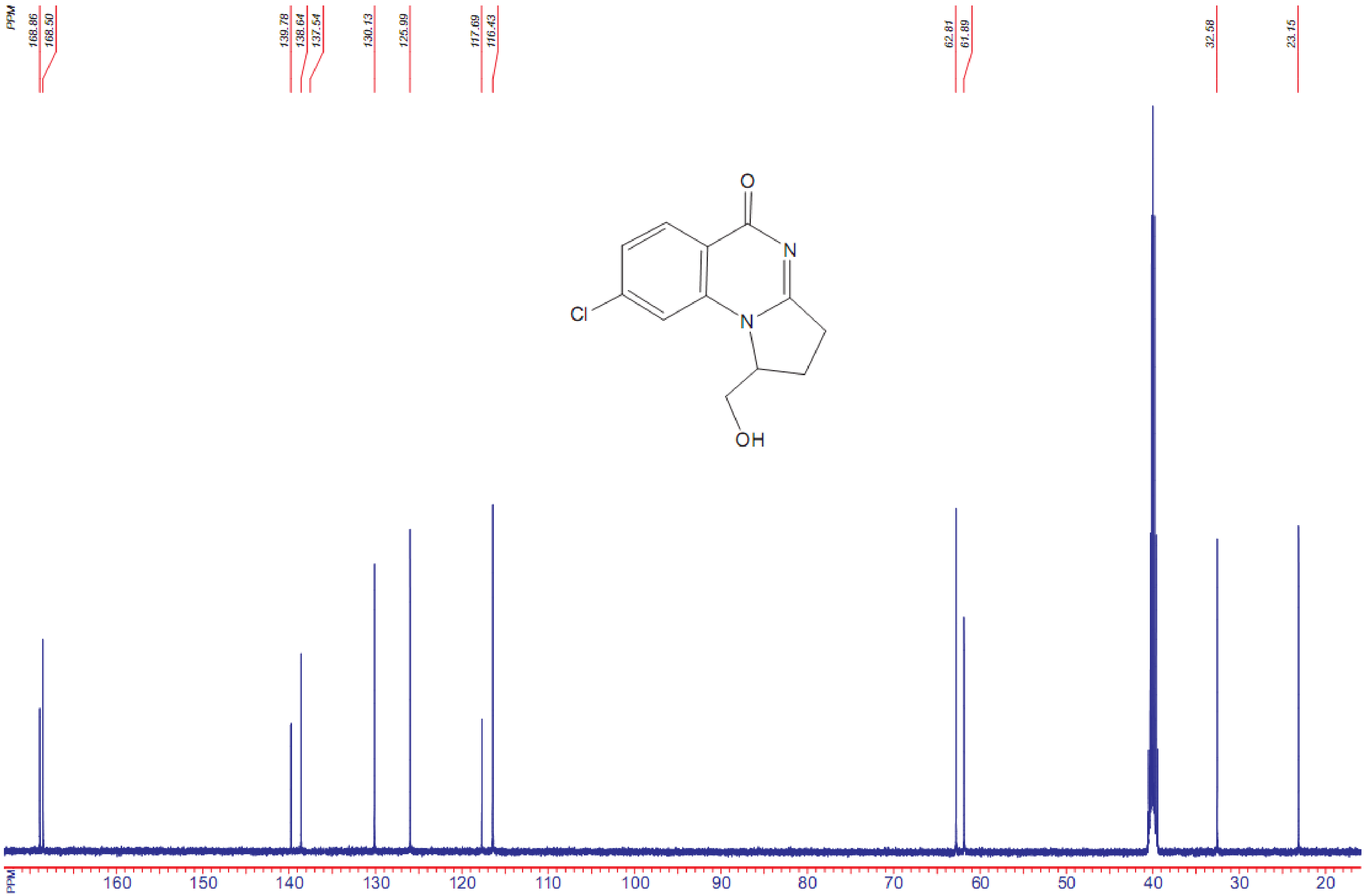

**File name: PHVM1426-6_C13.fid**	Operator:	SF: 125.6927 MHz	NSC: 0	PW: 3.27 usec, RG: 60	SI: 65536
Date: 15-May-2021	Solvent: dmso	SW: 31250 Hz	TE: 298 K	AQ: 1.02 sec, RD: 0.00 sec	13C (1H-decoupled)

Figure S71. ^{1}H NMR spectrum (400 MHz, DMSO-d_6) of compound 6j

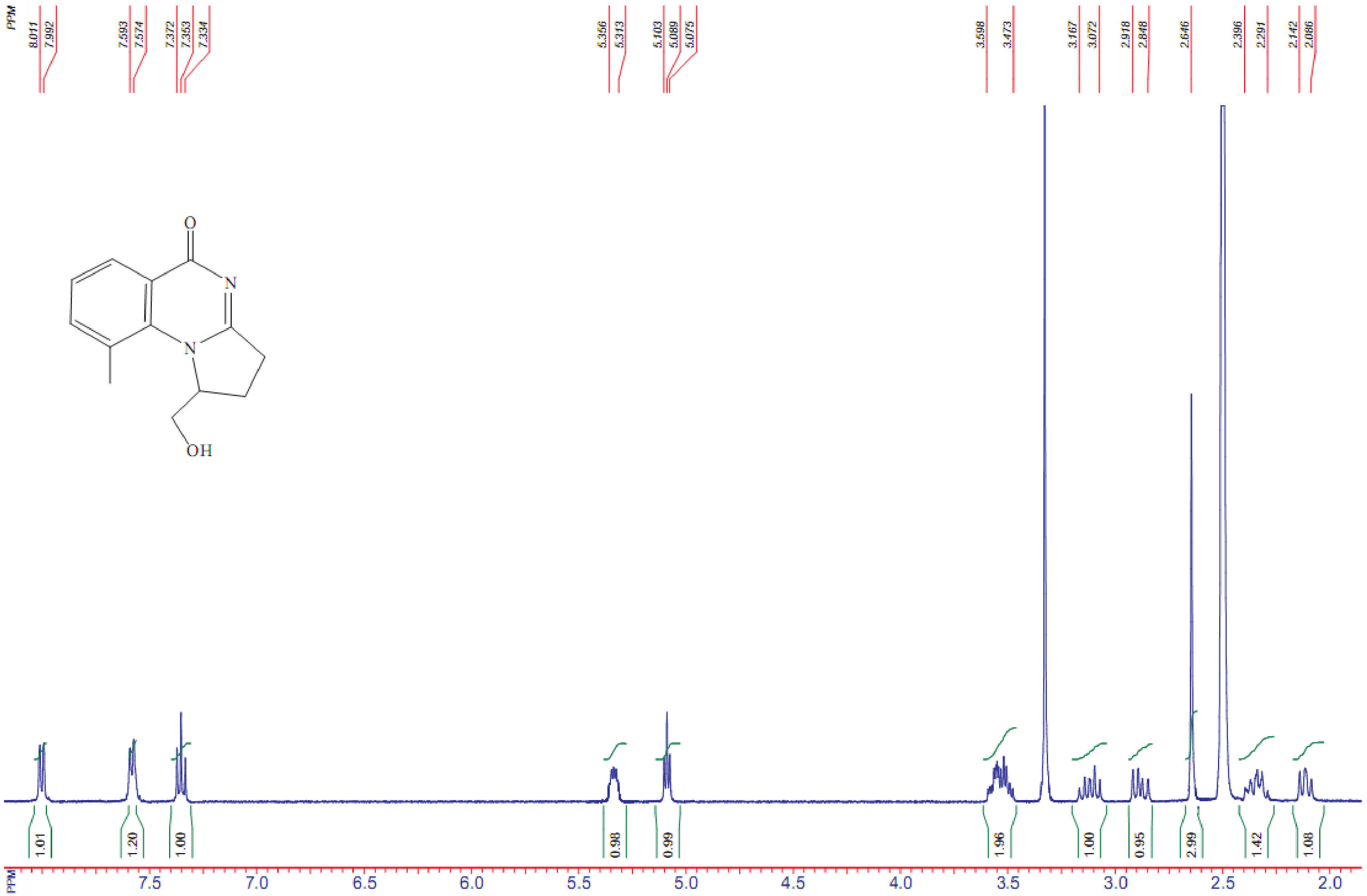

**File name: PHVM1442-21.fid**	Operator:	SF: 399.9753 MHz	NSC: 0	PW: 10.90 usec, RG: 24	SI: 32768
Date: 07-Jun-2021	Solvent: dmso	SW: 8000 Hz	TE: 298 K	AQ: 2.00 sec, RD: 0.00 sec	

Figure S72. ^{13}C NMR spectrum (125 MHz, DMSO-d_6) of compound 6j

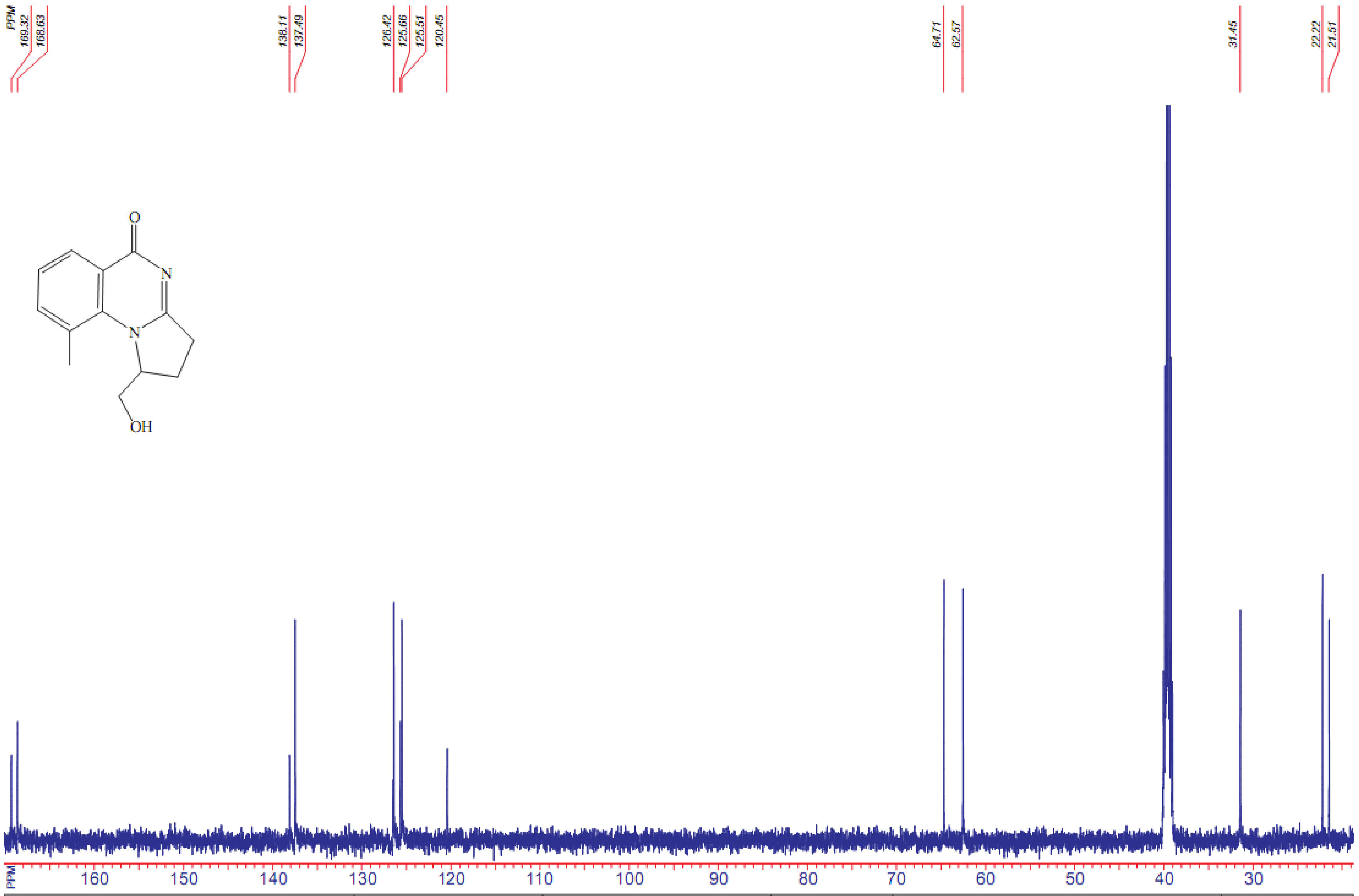

**File name: phvm1442-21_C13**	Operator: root	SF: 125.6681 MHz	NSC: 225	PW: 0.00 usec, RG: 51200	SI: 131072
Date: 08-Jun-2021	Solvent: DMSO	SW: 32680 Hz	TE: 683 K	AQ: 0.78 sec, RD: 0.00 sec	

Figure S73. ^{1}H **NMR spectrum (400 MHz, DMSO-*d*$_6$) of compound 6k**

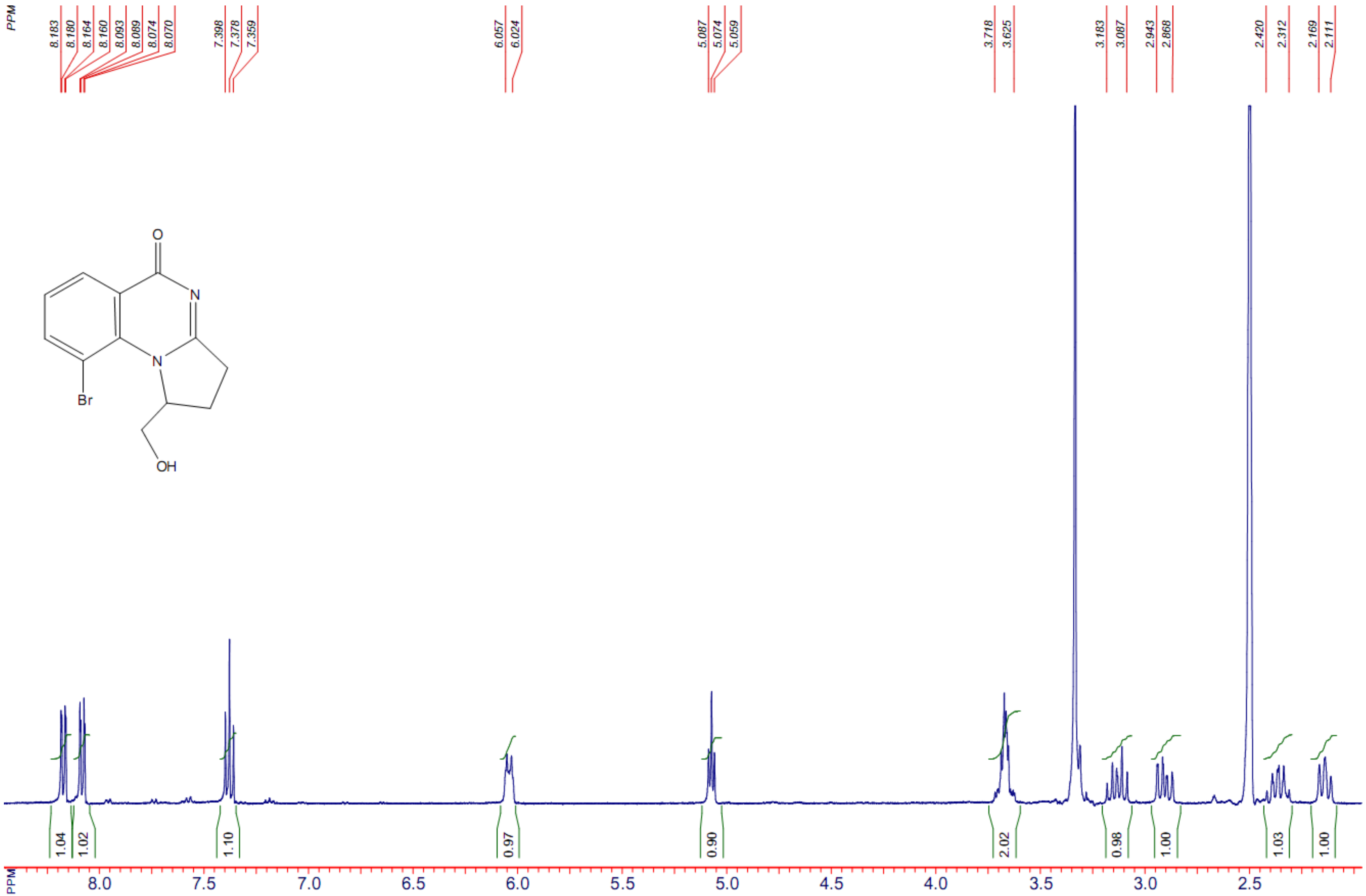

File name: PHvm1418-2.fid	Operator:	SF: 399.9753 MHz	NSC: 0	PW: 10.90 usec, RG: 24	SI: 32768
Date: 14-May-2021	Solvent: dmso	SW: 8000 Hz	TE: 298 K	AQ: 2.00 sec, RD: 0.00 sec	□

Figure S74. ^{13}C NMR spectrum (100 MHz, DMSO-d_6) of compound 6k

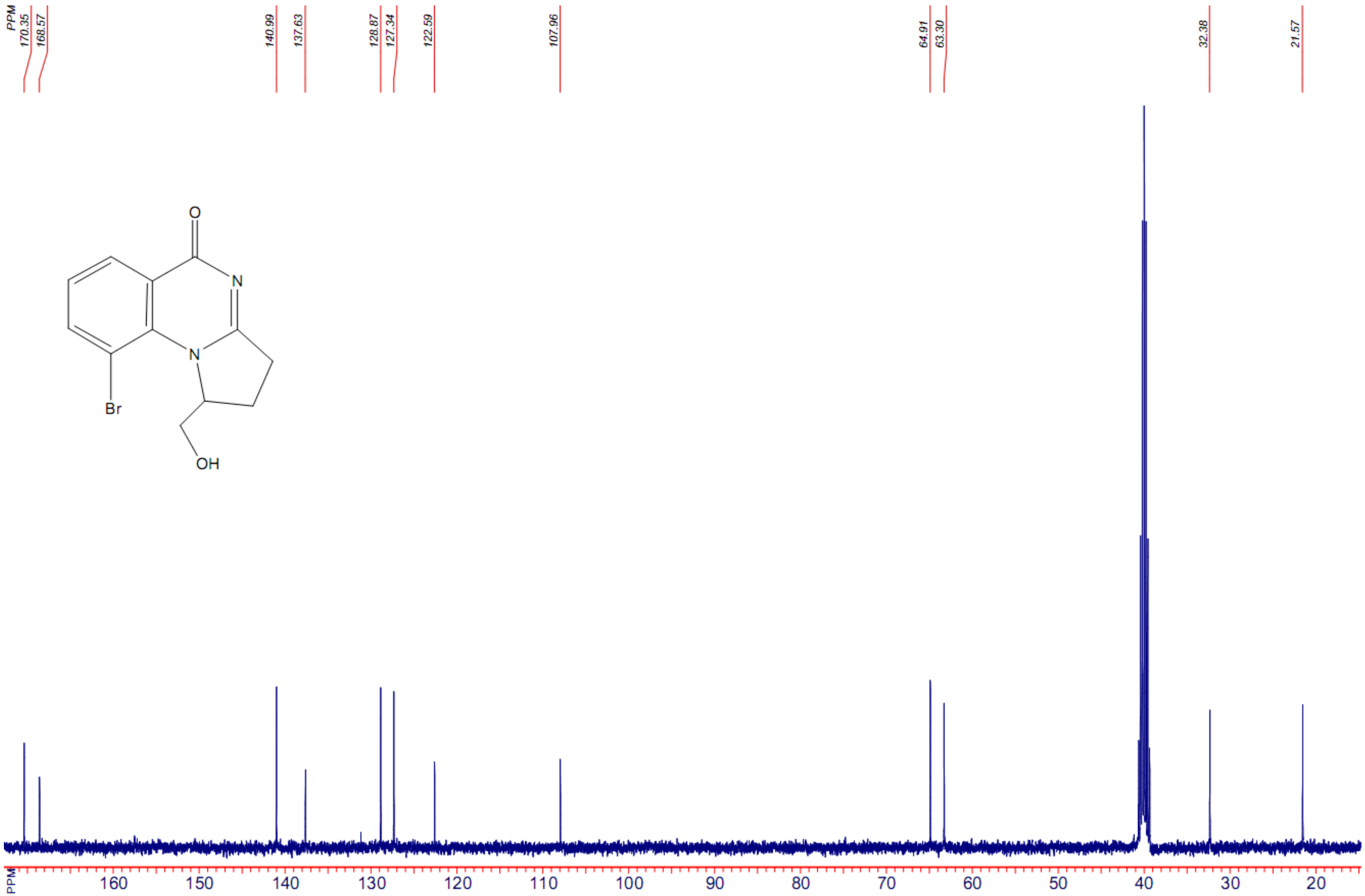

**File name: PHvm1418-2_C13**	Operator: nmr□	SF: 100.6128 MHz	NSC: 318	PW: 0.00 usec, RG: 1820	SI: 65536
Date: 21-May-2021	Solvent: DMSO	SW: 24038 Hz	TE: 298 K	AQ: 1.06 sec, RD: 0.00 sec	Parameter file, TOPSPIN□□Version 2.1□

Figure S75. **^{1}H NMR spectrum (400 MHz, DMSO-*d*6) of compound 6l**

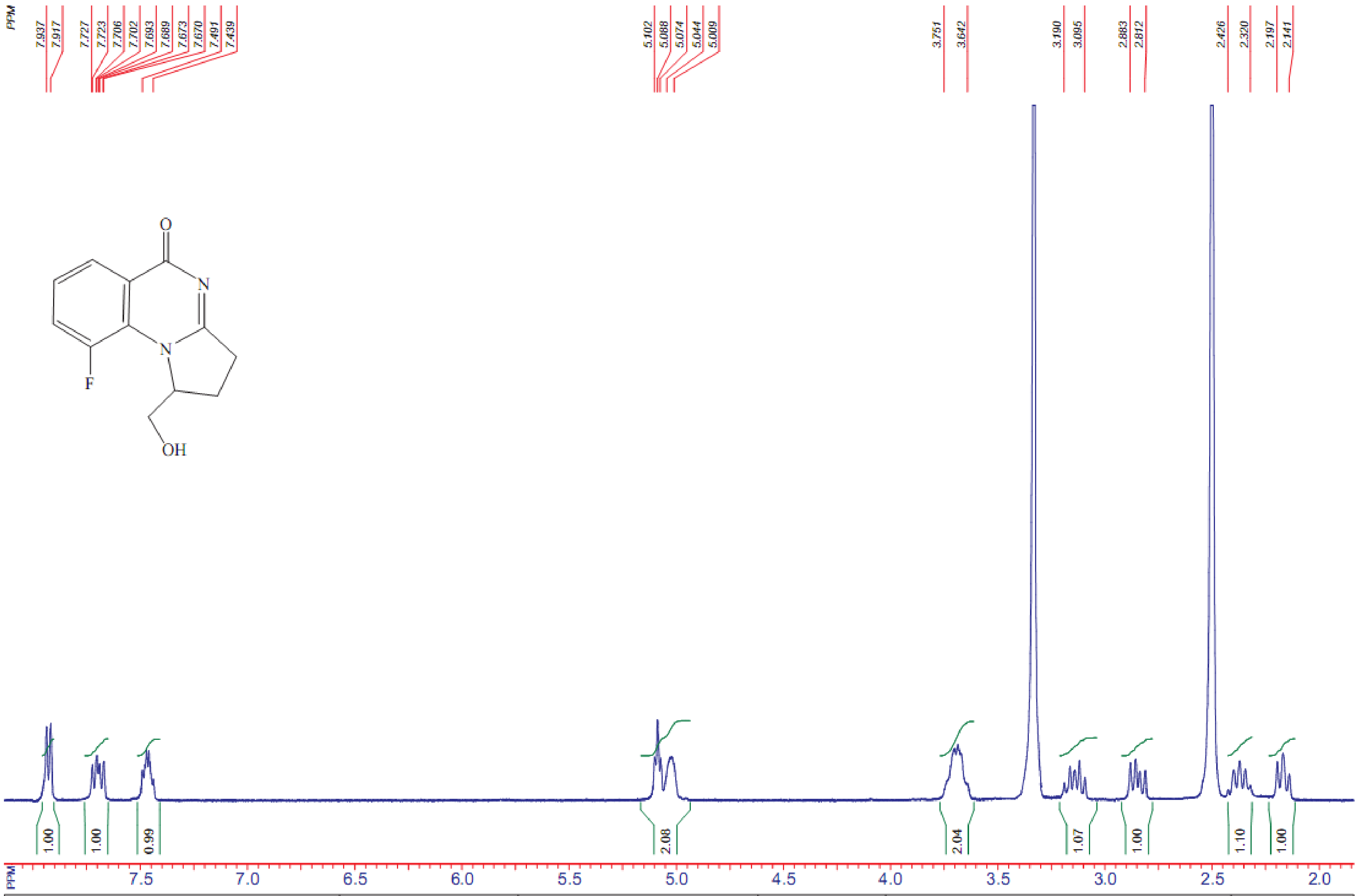

File name: PHvm1455-2.fid	Operator:	SF: 399.9753 MHz	NSC: 0	PW: 10.90 usec, RG: 24	SI: 32768
Date: 18-Jun-2021	Solvent: dmso	SW: 8000 Hz	TE: 298 K	AQ: 2.00 sec, RD: 0.00 sec	

Figure S76. ^{19}F NMR spectrum (188 MHz, DMSO-d_6) of compound 6l

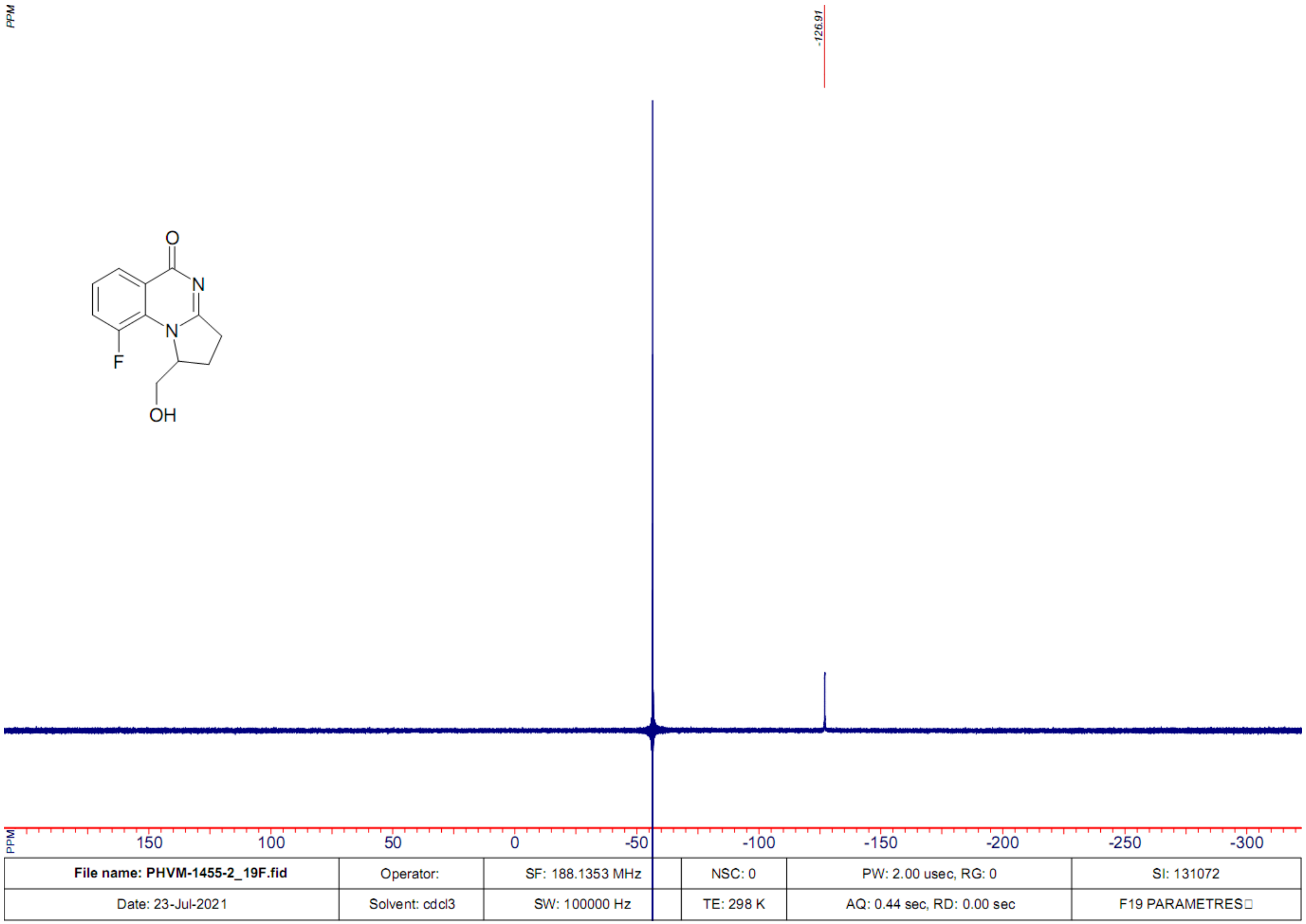

**File name: PHVM-1455-2_19F.fid**	Operator:	SF: 188.1353 MHz		NSC: 0	PW: 2.00 usec, RG: 0	SI: 131072
Date: 23-Jul-2021	Solvent: cdcl3	SW: 100000 Hz		TE: 298 K	AQ: 0.44 sec, RD: 0.00 sec	F19 PARAMETRES□

Figure S77. ^{13}C NMR spectrum (100 MHz, DMSO-d_6) of compound 6l

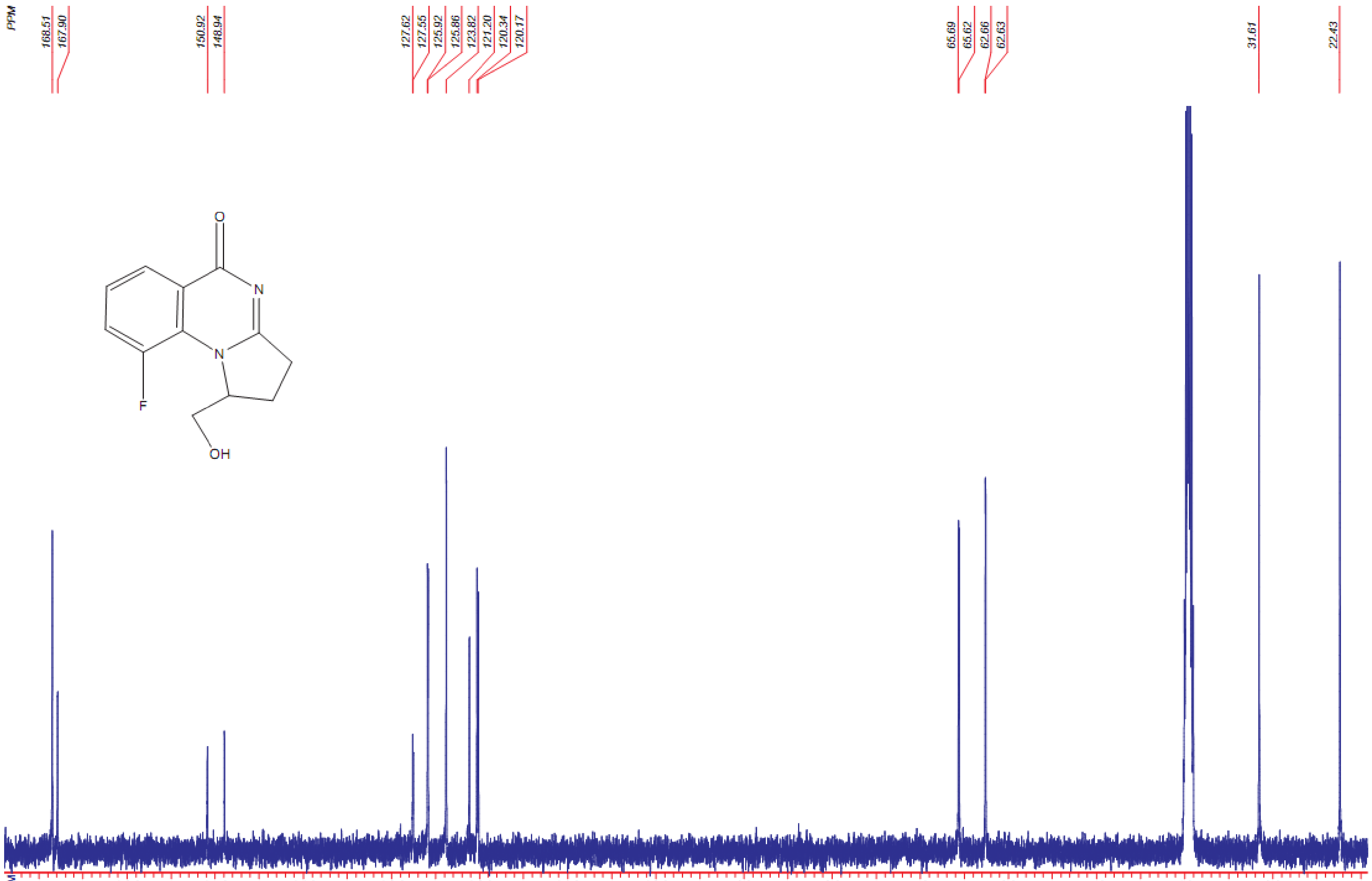

File name: PHVM1455-2_C13	Operator: root	SF: 125.6681 MHz	NSC: 396	PW: 0.00 usec, RG: 51200	SI: 131072
Date: 18-Jun-2021	Solvent: DMSO	SW: 32680 Hz	TE: 683 K	AQ: 0.78 sec, RD: 0.00 sec	
